# Responsive Organoselenium Dendritic Polymers: From Monodisperse Dendrimers to Self-Assembled Micelles for Advanced Therapeutic Applications

**DOI:** 10.1021/jacs.5c00811

**Published:** 2025-05-23

**Authors:** Natalia Sanz del Olmo, Jorge San Jacinto García, Yikai Yin, Ying Zhao, Moustapha Hassan, Michael Malkoch

**Affiliations:** † Department of Fibre and Polymer Technology, KTH Royal Institute of Technology, Teknikringen 56–68, 100 44 Stockholm, Sweden; ‡ Department of Laboratory Medicine, Experimental Cancer Medicine (ECM), Karolinska Institute, Center of Allogeneic Stem Cell Transplantation (CAST), Karolinska University Hospital Huddinge, 14186 Stockholm, Sweden

## Abstract

Selenium (Se) is a highly biologically active element, and its organic derivatives have attracted growing interest for their promising chemotherapeutic potential, largely due to their redox-modulating activity, which selectively affects cancer cells with high levels of reactive oxygen species (ROS). However, their high reactivity and susceptibility to spontaneous degradation limit their biomedical application. To harness their potential in the realm of nanomedicine, we present a new generation of therapeutically promising polymers that combine Se with 2,2-bis­(methylol)­propionic acid (bis-MPA)-based dendritic polymers, chosen for their high chemical versatility, low toxicity, and excellent biodegradability. Most examples in the literature about dendritic polymers feature dormant dendritic skeletons with active functional groups expressed only on their periphery, which severely limits their functional scope. In this work, monodisperse dendrimers and linear–dendritic (LD) polymers up to the third generation were developed, with the latter capable of self-assembling into dendritic micelles (∼20 nm). These systems feature Se at the dendritic core or peripheral branches in the form of monoselenide or diselenide bridges. Selenium incorporation demonstrated excellent compatibility with two key polyester synthetic approaches: anhydride chemistry and fluoride-promoted esterification (FPE). Both monoselenide and diselenide linkages introduced degradability and dynamic behavior in dendrimers and dendritic micelles. However, their biological activities differed significantly. Diselenide-containing dendrimers exhibited great anticancer potential against breast cancer cell lines, with IC_50_ values in the micromolar range. Among these, first-generation Se dendrimers stood out due to their promising selectivity toward cancer cells. In contrast, dendritic polymers incorporating monoselenides retained the high biocompatibility characteristics of bis-MPA dendritic constructs.

## Graphical abstract

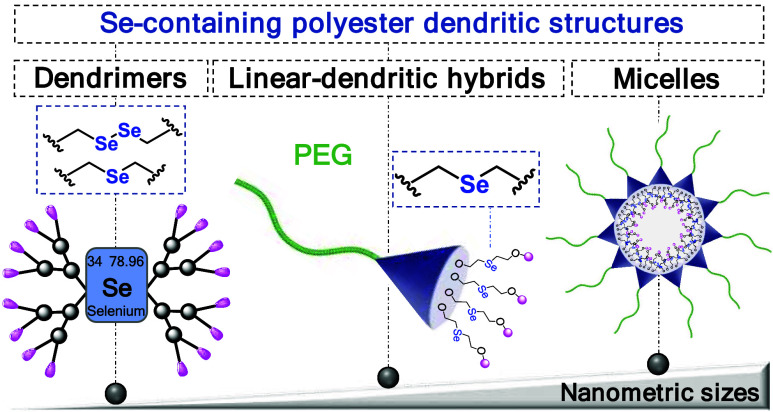

## Introduction

Dendrimers are the only monodisperse polymers that can be synthesized with a high degree of structural perfection as a consequence of taking advanced organic chemistry to a macromolecular level. From a structural perspective, dendrimers are highly branched molecules with high densities of peripheral functional groups. Their high structural accuracy results in a structure–property consistency, which is a particularly attractive quality in the field of nanomedicine. Within the extensive family of dendritic systems classified by nature, polyester and, more specifically, 2,2-bis­(methylol)­propionic acid (bis-MPA)-based dendrimers have gained considerable attention within biomedical research. Bis-MPA dendrimers meet all essential therapeutic requirements, including biocompatibility, biodegradability, and the absence of inflammatory cytokine induction during testing in primary human macrophages.^1^ The chemistry surrounding bis-MPA dendrimers is extremely versatile and has resulted in a wide variety of dendritic scaffolds, difficult to find in other dendritic families, ranging from monodisperse dendrons or dendrimers to polydisperse hyperbranched polymers and various linear–dendritic (LD) configurations containing poly­(ethylene glycol) (PEG).^2^

One interesting application of dendritic polymers is their use as drug carriers in nanomedicine. Unfortunately, the dominant number of dendritic polymers reported in the literature contains a dormant interior, with only their periphery utilized for modifications with therapeutic cargoes. To date, limited research has focused on the design of heterofunctional polyester dendrimers, where the functionalities are not only located at the periphery but also along the dendritic skeleton. One of these strategies involves the synthesis of dendrimers from AB_2_C monomers with orthogonal functionalities where A (COOH) and B (OH) functionalities were used for dendrimer growth, while the C (bromide, alkene, azide, etc.) function remained dormant throughout the growth steps.^3–5^ With the demand for more sophisticated scaffolds, smart molecules with the capability of degrading upon demand have been in the spotlight. In this context, Gillies et al. developed fully photodegradable bis-MPA dendrimers containing *o*-nitrobenzyl esters.^6^ Additionally, our group developed a highly sophisticated strategy in which the internal functionalities are embedded into the dendritic skeleton. With that purpose in mind, our group took advantage of the properties provided by sulfur (S) and introduced this element as part of internally queued disulfide bridges into polyester dendrimers. This new generation of self-immolative dendrimers represents the state of the art and led to flawless macromolecular templates that selectively rupture into a set of monomeric mercaptans, inducing a significant increase of reactive oxygen species (ROS) in human lung carcinoma A549 cells.^7^

The importance of S has been acknowledged since its discovery. This element is one of the most abundant elements in the human body, widely incorporated into proteins and vitamins as well as other essential metabolites and cofactors like lipoic acid.^8^ The role of S in the body is of such importance that a thiol imbalance has been shown to associate with multiple medical disorders including Alzheimer’s, HIV, and cancer.^9^ In the same chalcogen group of the periodic table as S is Selenium (Se), which is an essential trace element for the human body, even though its biomedical role has been controversial for several years. Se was discovered 40 years later than S by Jöns Jacob Berzelius^10^ and it was considered highly toxic initially. It was not until 1957 when Klaus Schwartz and Calvin Foltz recognized this element as key for preventing lesions in the liver, blood vessels, and muscles in rats and chickens.^11^ Since then, the potential of Se in biological processes has been widely investigated and there has been a growing interest in the application of Se in different areas.^12^ As a trace element, it is vital to maintain various functions of the body, playing a key role in Se-dependent enzymes’ function to keep a redox balance in cells and prevent severe diseases.^13,14^ Encouraged by the outstanding biological activity of this element, organic selenium chemistry has advanced rapidly and multiple examples of organoselenium compounds have been developed with promising anticancer activities.^15^ One of the promising organoselenium compounds is ethaselen that reached phase 1c of clinical trials (NCT02166242) for treating patients with non-small-cell lung cancer.^16^

In search of reduced toxicity from Se-based treatments, nanomedicine has shown that Se in various nanoforms can display a superior antioxidant and antitumor activity with lower toxicity.^17,18^ The concept to apply Se in the polymer-based therapeutics field was first reported in 2010 by Xu et al. in which Se polymers were described as promising biomaterials for controlled drug release.^19^ In addition to the potent anticancer properties of Se-based compounds, its large atomic radius and low electronegativity compared to S endow Se-based polymers with dynamicity and responsiveness to several stimuli. As a result, they display favorable biological properties upon exposure to the physiological environment. Over the past decade, there has been a rapid increase in the number of polymers containing Se reported in the literature through different novel synthetic methods.^20,21^ The challenges in the development of Se-containing polymers with structural and functional diversity include the lack of safe monomers, the absence of efficient synthetic approaches, and the poor stability of Se-involving covalent bonds. Additionally, the challenge becomes more striking when it refers to its incorporation into dendritic polymers where few examples are reported for hyperbranched dendritic polymers^22,23^ or dendrimers based on Fréchet or Poly­(lysine) dendritic skeletons.^24,25^ To date, there are no examples in the literature where Se has been incorporated into polyester dendrimers.

Seeking new horizons of sophistication, we propose a new generation of dendrimers, where Se can be incorporated into bis-MPA dendritic scaffolds. Polyester dendrimers have been successfully synthesized with Se located at the dendritic core or at the dendritic branches, leaving the periphery available for further modifications. The stability of the synthesized dendrimers has been explored through MALDI-ToF at relevant physiological pHs as well as in the presence of glutathione (GSH) in order to mimic extracellular and intracellular environments. The integration of Se has been expanded to amphiphilic linear–dendritic (LD) bis-MPA polymers which have been synthesized containing Se in the dendritic branches as suitable candidates for the formation of micellar dynamic structures with a controlled disassembly where Se plays a crucial role. The biological outcome of dendrimers as well as LDs containing Se has been validated through cytotoxicity experiments against breast cancer cells and noncancer cell lines. In order to gain a better understanding of the biological performance of dendrimers, peripheral postfunctionalization has been carried out as a proof of concept with PEG functionalities as well as positive charges.

## Results and Discussion

### Se-Containing Bis-MPA Dendritic Prodrugs with a Biologically Active Skeleton

Starting from elemental Se as a powder, monodisperse bis-MPA dendrimers have been synthesized and fully characterized, with Se moieties either at the dendritic core or embedded into the dendritic branches (Figure 1). Inspired by previous works related to bis-MPA dendrimers with internal disulfide bridges,^7,26^ our first goal was to take advantage of the properties provided by Se and develop a new generation of bis-MPA dendrimers where S is substituted by this unique element (Figure 1, left). The path to obtaining diselenide-functionalized dendrimers started with the synthesis of 2-hydroxyethyl diselenide from elemental Se following a protocol adapted from the literature.^27,28^ For this reaction, hydrazine hydrate was selected as a reducing agent in the presence of KOH using water as solvent, followed by an overnight reaction with bromoethanol dissolved in THF. As reported in the literature, one of the main challenges related to the synthesis of Se-based compounds is the obtention of high-purity derivatives.^29^ With the use of strong reducing agents, these reactions lead to the formation of complex mixtures of polyselenides (R–Se*_n_*–R), the abundance of which often varies between different batches (Figure S1).^13^ While organic polysulfides (R–S*_n_*–R) are well-documented species,^30^ polyselenides are much less extensively studied in the literature due to their higher reactivity compared to their sulfur counterparts, which make them more prone to oxidation and decomposition. To ensure high purity of the diselenide derivative and to isolate it from monoselenide or polyselenide species formed during the reaction process, we introduced silica chromatography as an additional purification step.

**Figure 1:**
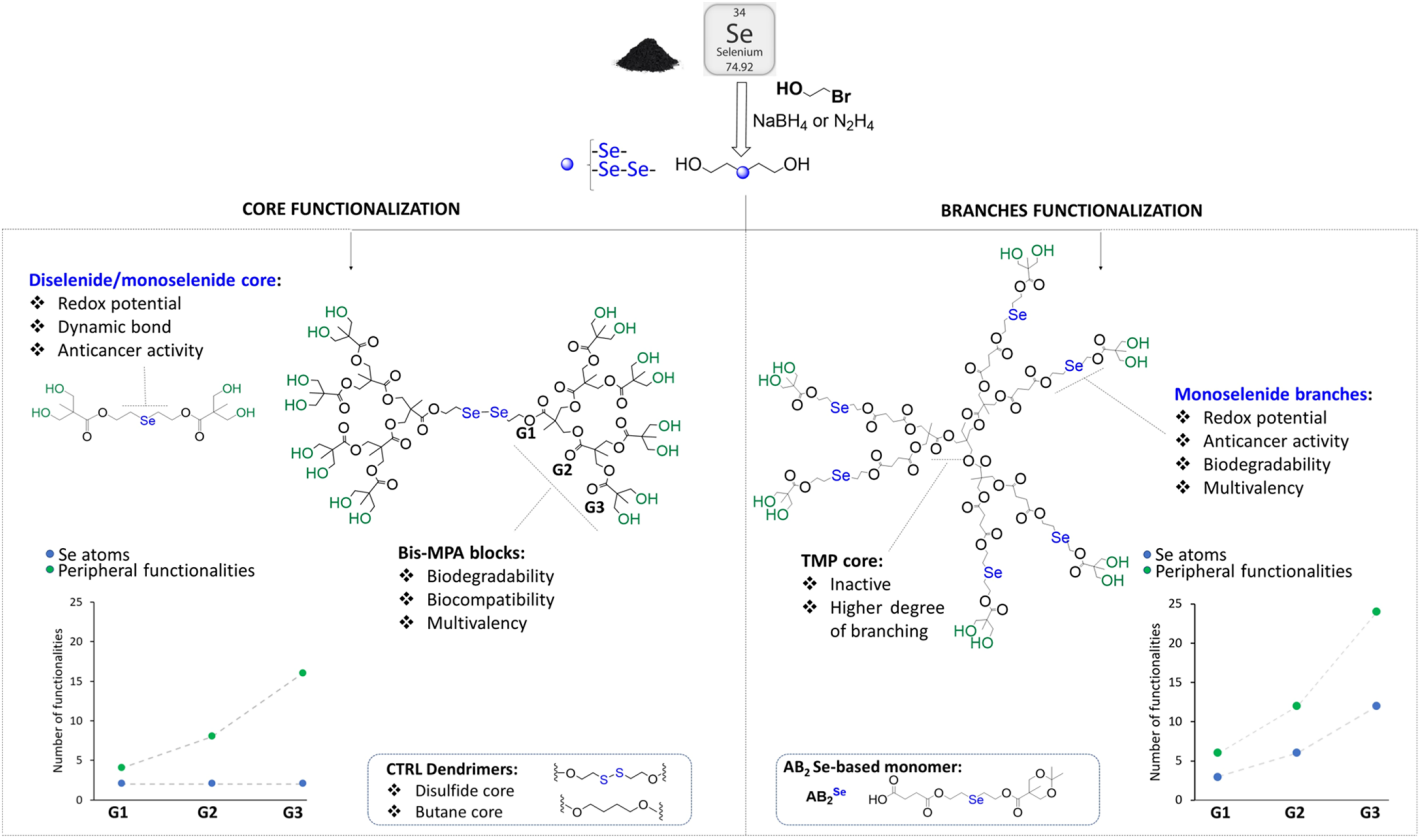
Selenium (Se)-containing dendritic compounds synthesized in this work where Se has been included at the core of the dendritic polymer (left) or at the dendritic branches (right).

The presence of hydroxyl functionalities in the synthesized diselenide monomer allowed for the dendritic growth through anhydride-based esterification reactions using acetonide-protected bis-MPA as a building block. For these reactions, bis-MPA anhydride was formed with *N*,*N*′-dicyclohexylcarbodiimide (DCC) as the dehydration agent and reacted *in situ* with the diselenide core in the presence of pyridine as a base and DMAP as a catalyst. Subsequent steps of deprotection with DOWEX and dendritic growth allowed for the obtention of a family of water-soluble dendrimers from first to third generation (Se_2_-G*n*-(OH)*_m_*, where *n* = 1–3 and *m* = 4–16), for which the number of Se functionalities remained constant, whereas the number of peripheral hydroxyl functionalities increased up to 16 for the highest generation (Figure 1, left). As control molecules for the degradation studies as well as for the preliminary biomedical evaluation, dendrimers containing disulfide bridges at the core (S_2_-G*n*-(OH)*_m_*, where *n* = 1–3 and *m* = 4–16)^26^ as well as dendrimers with a hydrocarbon chain derived from butanol as the core (But-G*n*-(OH)*_m_*, where *n* = 1–3 and *m* = 4–16), have been synthesized up to third generation following the same synthetic rational.

On the other hand, the obtention of dendrimers with diselenide functionalities at the dendritic branches required of the synthesis of an asymmetric diselenide (AB_2_^Se–Se^) monomer (Figure 1, right) analogous to that previously described for the synthesis of bis-MPA dendrimers with queued disulfide bridges.^7^ However, the isolation of this AB_2_^Se–Se^ monomer was prevented due to a fast visible light-driven diselenide-based metathesis^31^ where the asymmetric diselenide monomer (A–Se–Se–B) after silica chromatography coexisted with a mixture of two symmetric diselenides (A–Se–Se–A and B–Se–Se–B) (Figure S3).

In addition to the presence of diselenide bridges as a source of dynamicity and anticancer potential, Se in the form of monoselenide has also attracted wide attention. Even though monoselenides are much less reported in the literature as antioxidant or anticancer agents compared to diselenides, these compounds have shown a strong potential as antitumoral compounds with antiproliferative, cytotoxic, and apoptotic activity.^32^ The inclusion of monoselenides into dendritic skeletons has also been explored and required the synthesis of 2-hydroxyethyl selenide. Following adapted protocols to the ones published in the literature,^33^ this monomer was obtained from elemental Se using sodium borohydride as a reducing agent. From this derivative, an asymmetric monomer based on selenide (AB_2_^Se^) was successfully achieved following a three-step synthetic strategy. The obtained monomer contained a carboxylic acid group (A) as well as acetonide-protected diols (B_2_), similar to the traditional bis-MPA, but in an extended configuration including Se as monoselenide between the functionalities (Figure 1, right). Hydroxyl-functionalized bis-MPA dendrimers^34^ were finally decorated with the monoselenide monomer using fluoride-promoted esterification (FPE) chemistry, generating dendritic scaffolds where the number of both Se atoms as well as peripheral functionalities increased with the dendritic generation. The highest dendritic generation achieved was the third-generation acetonide-protected (TMP-G*n*-(Se)*_m_* (Ac)*_m_*) (Figure 1, right). Finally, from the synthesis of the asymmetric Se monomer (AB_2_^Se^), the first-generation dendrimer with monoselenide functionalities at the dendritic core (Se-G1-(Ac)_2_) was also isolated and deprotected with DOWEX (Se-G1-(OH)_4_) to evaluate the impact of the mono/diselenide on the biomedical performance.

All synthesized compounds were fully characterized through NMR spectroscopy (^1^H, ^13^C, and ^77^Se) as well as MALDI-ToF and SEC (Figure 2). ^77^Se NMR confirmed the stability of the mono/diselenides with respect to oxidation/reduction reactions due to the presence of a single peak at either 281 ppm (Figure 2A) or 121 ppm for the hydroxyl-functionalized diselenide and monoselenide-based dendrimers, respectively. In spite of the basic conditions required for the dendritic growth and the acidic DOWEX involved in the deprotection steps, Se-based covalent bonds remained stable, showcasing the compatibility of two of the most robust synthetic approaches involved in the synthesis of polyester dendrimers: anhydride and FPE, with the presence of internal Se moieties. All dendrimers from both families had low polydispersity values (∼1), as confirmed by SEC analysis (Figure 2B). The MALDI-ToF spectra of the diselenide derivatives, collected in both linear and reflector modes, showed peaks attributed to the diselenide dendrimers and an additional peak corresponding to the selenol derived from the breaking of the diselenide bridge (Figure S35). This phenomenon was not observed for the analogous disulfide dendrimers previously reported^26^ and was a result of the weaker strength of the Se–Se bond (172 kJ mol^–1^) compared to the S–S bond (240 kJ mol^–1^) and C–C bond (346 kJ mol^–1^).^35^ For the first- and second-generation monoselenide dendrimers, MALDI-ToF showed only one peak attributed to the dendrimer with no signs of degradation (Figure 2C). For the third-generation monoselenide acetonide-protected dendrimer, a good MALDI-ToF spectrum could not be obtained due to the incompatibility shown with the methods used.

**Figure 2:**
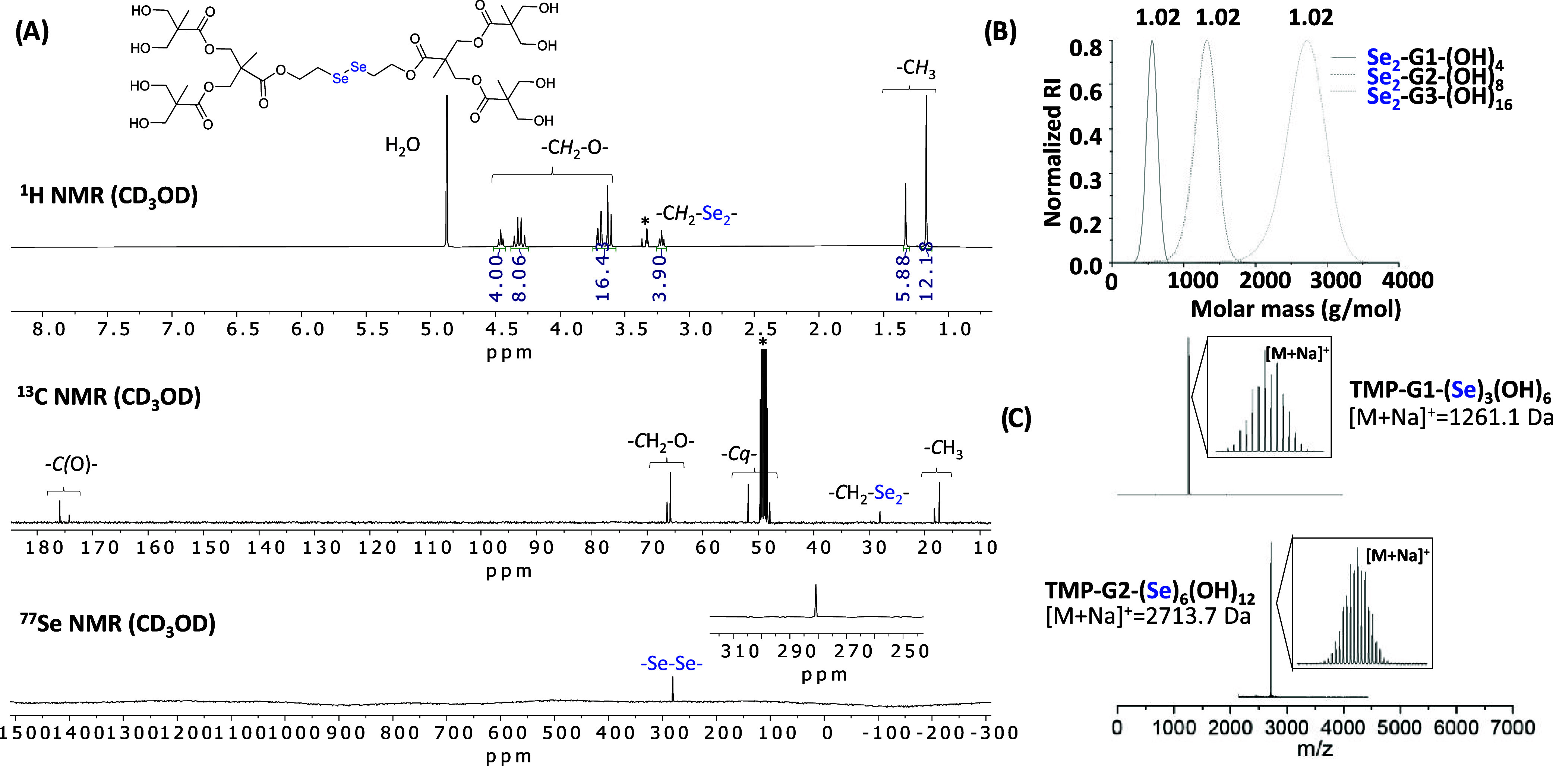
(A) Stacked ^1^H, ^13^C, and ^77^Se NMR spectra of the second-generation diselenide derivative (Se_2_-G2-(OH)_8_) in CD_3_OD (*). (B) SEC overlay of the three generations of diselenide-based dendrimers (Se_2_-G1-(OH)_4_, Se_2_-G2-(OH)_8_, and Se_2_-G3-(OH)_16_) indicating on top of the curves the polydispersity of each sample. (C) Stacked MALDI-ToF spectra of the first- and second-generation dendrimers containing monoselenide functionalities in the dendritic branches (TMP-G1-(Se)_3_-(OH)_6_ and TMP-G2-(Se)_6_-(OH)_12_).

Collectively, these results are promising for the polymer scientific community, where hydroxylated polymers could be functionalized with Se moieties using both anhydride chemistry and FPE.

### Degradation Evaluation under Physiological Conditions

To place Se-based dendrimers in the context of biomedical applications, it is crucial to determine the stability of the dendritic nanocarrier under physiological conditions.

From a stability perspective, we identified intriguing behavior in Se dendrimers. Despite the presumably more stable configuration of monoselenide dendrimers, hydrolytic degradation driven by the presence of succinic moieties was observed. This degradation increased with dendritic generation after prolonged storage at −20 °C (Figure S42), suggesting the need for their use within a short period after preparation. The higher long-term stability observed in the family of diselenide dendrimers led to their selection for further degradation experiments and biological assays.

In this context, both redox and pH responsiveness were evaluated for the third-generation diselenide dendrimer (Se_2_-G3-(OH)_16_) as a proof of concept. These experiments were performed in parallel with control dendrimers of third-generation with disulfide (S_2_-G3-(OH)_16_) as well as hydrophobic chain (But-G3-(OH)_16_) as cores.

#### Redox Responsiveness

In tumor microenvironments, the concentration of glutathione (GSH, 10 mM) is approximately 3 orders of magnitude higher than that of the extracellular environment (∼10 μM) serving as an essential trigger when targeting selectivity in cancer treatment.^36^ The responsive character to GSH of the third-generation dendrimers with dynamic bonds at the dendritic core (Se_2_-G3-(OH)_16_ and S_2_-G3-(OH)_16_) as well as a control dendrimer (But-G3-(OH)_16_) was evaluated at physiological pH values of 7.4 and 37 °C.

As supported by MALDI-ToF experiments, the presence of S or Se plays a crucial role in the stability (Figure 3A). Under intracellular conditions, while the molecular peak for the S-based dendrimers can be observed up to 30 min (red star), for the Se counterpart, in less than 15 min, complete rupture of the dendritic system was observed. Additionally, it was possible to observe a notable difference with regard to the presence of the thiol/selenol as a reduced product (green star). Although for the disulfide derivative the peak of the thiol can be observed even after 1 h of incubation, for the diselenide, it can only be observed at time zero, and afterward, just the peak attributed to the dendron without Se can be identified (purple star). These results agree with the lower-energy bond attributed to diselenides compared to disulfides. In contrast, when evaluating extracellular conditions, due to the low concentration of GSH, the structural integrity of both derivatives remained nearly intact, as supported by MALDI-ToF and ^77^Se NMR.

**Figure 3:**
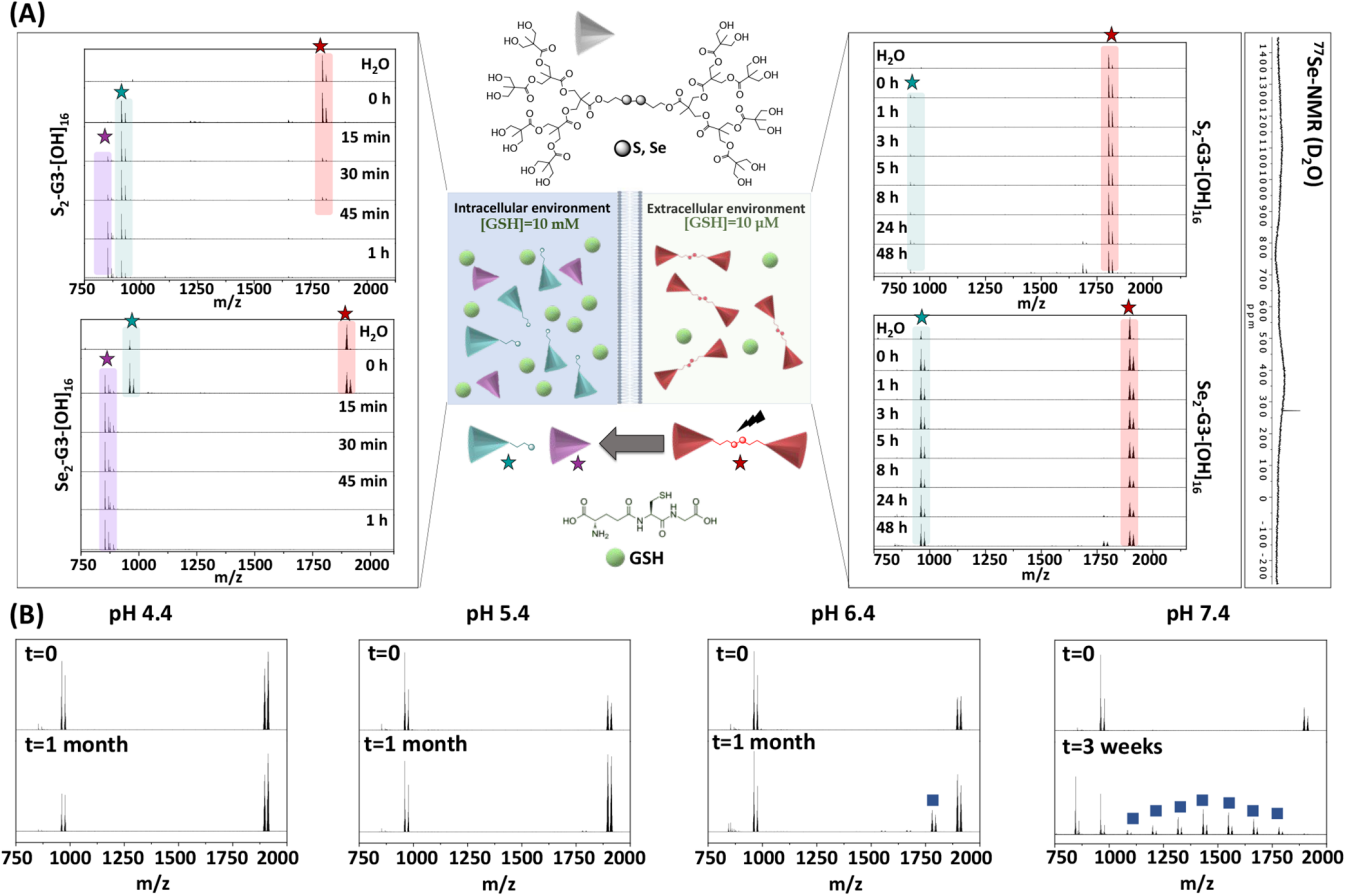
Degradation study for the diselenide third-generation dendrimer (Se_2_-G3-(OH)_16_) at 37 °C under the following settings: (A) pH 7.4 and glutathione (GSH) at intracellular (10 mM, left) and extracellular (10 μM, right) conditions analyzed through MALDI-ToF and ^77^Se NMR. Study performed at different incubation times up to 1 h for intracellular conditions and up to 48 h when mimicking extracellular environment; (B) different pHs (4.4, 5.4, 6.4, and 7.4) comparing time zero with 3 or 4 weeks of incubation with MALDI-ToF.

As predicted, the structure of the control dendrimer But-G3-(OH)_16_ was completely unaltered under both extracellular and intracellular conditions due to the absence of any redox-responsive bonds in its structure (Figures S47 and S48).

#### pH Responsiveness

For the same family of dendrimers, the stability was also assessed at various physiological pHs (4.4, 5.4, 6.4, and 7.4). Due to the presence of ester bonds in the structure, these dendrimers are highly susceptible to hydrolysis which is vastly dependent on the pH. At higher pHs, the hydrolysis increased for all families of dendrimers (Figures S43–S46), as previously reported in the literature for bis-MPA-based dendrimers.^1^ Additionally, the core was not altered as a consequence of the variations on the pH and the depolymerization proceeded by the gradual loss of all bis-MPA moieties from the outermost layer, represented in Figure 3B with blue squares.

### Se-Containing Linear–Dendritic (LD) Polymers

To showcase the versatility of the incorporation of Se into bis-MPA-based dendritic scaffolds, LDs containing monoselenide were also synthesized as a proof of concept. These structures amalgamated both the properties of a linear chain of mPEG5k with those provided by the bis-MPA dendritic scaffold. The mPEG was the main source of hydrophilicity as well as biocompatibility. From one of the ends of the polymeric chain emerged the different dendritic generations of bis-MPA dendrons providing hydrophobicity, biodegradability, multivalence, as well as responsive behavior as a consequence of the monoselenide moieties embedded into the polyester dendritic skeleton (Figure 4A).

**Figure 4:**
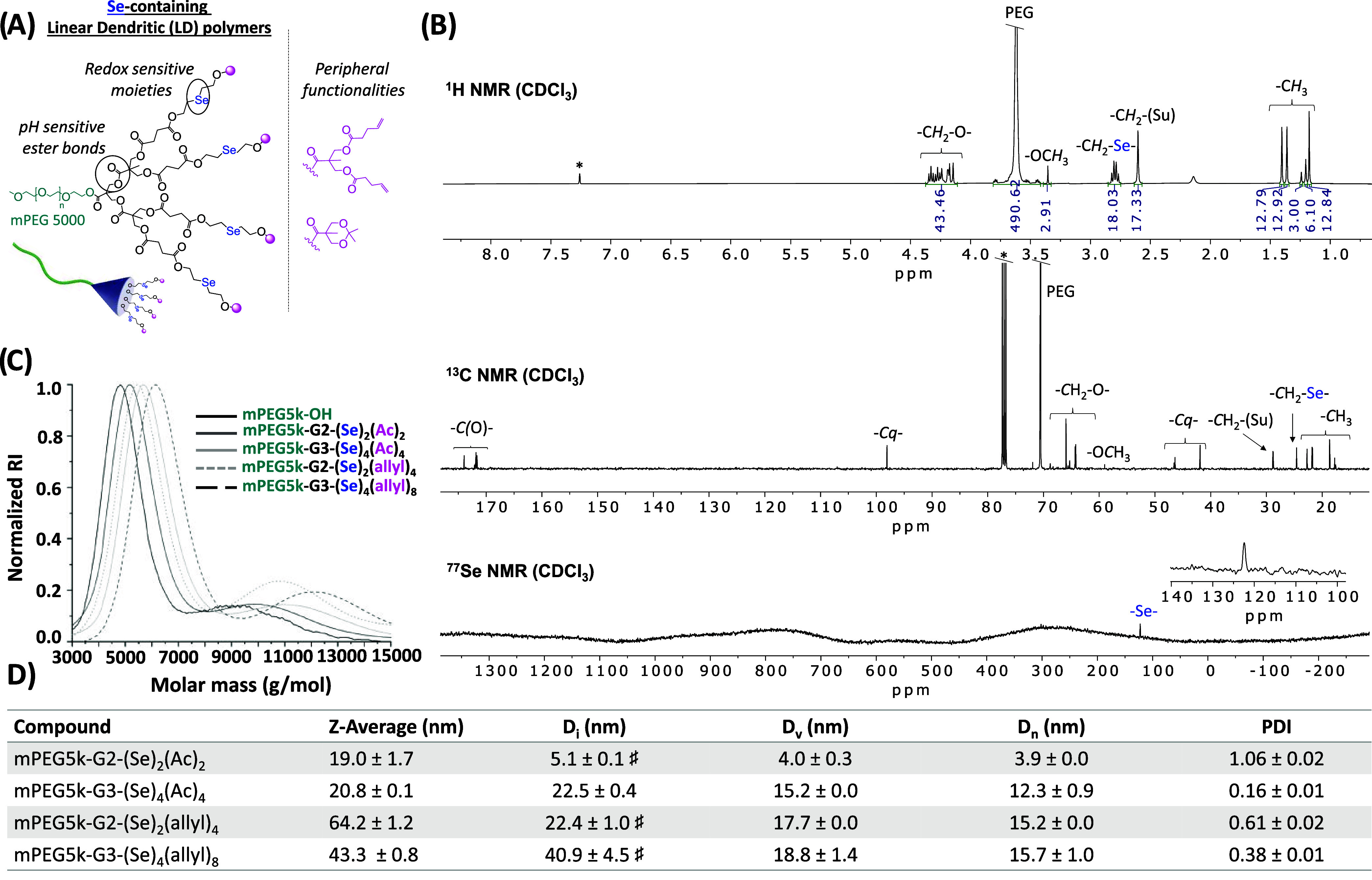
(A) Third-generation Se-containing linear–dendritic polymer with acetals or allyls as peripheral functionalities. (B) ^1^H, ^13^C, and ^77^Se NMR of mPEG-G3-(Se)_4_(Ac)_4_ in CDCl_3_ (*). (C) SEC overlay of the commercially available PEG5k–OH together with the synthesized Se-containing LDs of second and third generations. (D) DLS data obtained for the Se-LDs following the evaporation method at 5 mg/mL. ^⧧^Larger aggregates observed. Mean values accompanied by standard deviation, *n* ≥ 3.

LDs have attracted wide attention from the scientific community in the field of biomedicine for their capacity to self-assemble into drug delivery systems (DDS). As previously reported by Fréchet et al. LD hybrids based on PEG-bis-MPA conjugates were shown to form micelles with hydrodynamic diameters in the range of 17–50 nm.^37^ This size is ideal when targeting drug delivery applications in cancer treatment considering crucial factors such as clearance by the kidney, uptake by the tumor, and removal from the bloodstream by the liver and reticuloendothelial system.^38^ For the synthesis of these derivatives, the same asymmetric monomer containing monoselenide functionalities involved in the synthesis of dendrimers was utilized (AB_2_^Se^) (Figure 1, right). We anticipated that this architecture would not only promote micelle formation but also confer enhanced chemical stability to the AB_2_ monoselenide monomer by shielding sensitive groups, particularly the selenium moieties, within the hydrophobic micellar core. Starting from hydroxyl-functionalized LDs synthesized following previously described methodologies,^39^ second- and third-generation acetonide-protected LDs containing Se (mPEG5k-G*n*-(Se)*_m_* (Ac)*_m_*), where *n* = 2 or 3 and *m* = 2 or 4, were achieved by using anhydride chemistry through a divergent approach. Subsequently, these LDs were deprotected with DOWEX to obtain hydroxyl-functionalized LDs (mPEG5k-G*n*-(Se)_*m*/2_(OH)*_m_*, where *n* = 2 or 3 and *m* = 2 or 4), which were finally functionalized with allyl peripheral moieties as a proof of concept of postfunctionalization (mPEG5k-G*n*-(Se)_*m*/2_(Allyl)*_m_*, where *n* = 2 or 3 and *m* = 2 or 4). Allyl functionalities can aid in self-assembly and provide a higher hydrophobicity. Moreover, alkene moieties have proved to be an efficient functionality for the formation of nanogels from micelles through thiolene click chemistry (TEC).^40^ For this purpose, 4-pentenoic anhydride was utilized as a proof of concept.

The purity and structural integrity of the synthesized LDs were confirmed by NMR (^1^H, ^13^C, and ^77^Se) (Figure 4B) as well as SEC (Figure 4C).

The hydrolytic stability of the second-generation LD-containing acetonide functionalities (mPEG5k-G2-(Se)_2_(Ac)_2_), as an example, was evaluated by NMR in D_2_O (pH = 5.5) at 37 °C over various time intervals (Figure S49). The results suggest that in the presence of water the signal of the methylene groups in the succinic moiety at around 2.60 ppm remains intact for up to 10 days, along with the signals attributed to the PEG and dendritic skeleton, whose integrals also remain unaltered. Additionally, the hydroxyl groups remain protected in the short term with only ∼20% deprotection observed after 6 days of incubation.

### Micellar Arrangement of Se-LDs in Aqueous Environments

Satisfied with the purity of the isolated LD hybrids, their capacity to form micelles was evaluated using different methods, including direct solution in aqueous environments, thin-film adsorption, microprecipitation, and evaporation. For these experiments, both acetonide-protected and allyl-functionalized LDs were selected due to the increased hydrophobicity compared to the hydroxyl-functionalized counterparts.

First, the effect of dissolving the dendritic polymer in water was analyzed by ^1^H NMR. For this study, the LDs functionalized with acetonide groups were chosen as a proof of concept. Spectra of the LDs dissolved in either organic solvent (CDCl_3_) or water (D_2_O) were compared. The second-generation derivative showed a well-defined ^1^H NMR in both solvents, while the third-generation LD displayed much broader signals in aqueous solvent compared to the organic solvent, which indicted possible self-assembly of the third-generation derivative in aqueous environments (Figure S50).

To corroborate these results, the size of the polymers was analyzed by dynamic light scattering (DLS). The hydrodynamic diameter for the free mPEG5k-OH was 5 nm (*Z*-Average) at 1 and 10 mg/mL in PBS at 25 °C (Figure S51). The hydrodynamic diameters of the second- and third-generation acetonide-protected LDs were 9 and 20 nm, respectively, at 1 mg/mL, and their polydispersity values were 0.5 and 0.05, respectively. The reason for the high polydispersity obtained for the second-generation LD was due to the presence of larger populations due to a possible aggregation of the polymer. When the diameter was analyzed for the second-generation LD in terms of volume (*D_v_*) or number (*D_n_*), a value of approximately 4 nm was obtained, indicating that most of the polymer remained in solution and did not assemble into larger constructs. However, for the third-generation derivative, a monomodal distribution was obtained with a low polydispersity, and its size in volume or number was around 14 and 15 nm, respectively (Figure S51). This indicates that the third-generation LD might self-assembly, as suggested by the NMR experiments. To promote the self-assembly of the second-generation derivative, different protocols for micelle formation were tested, including thin-film adsorption, microprecipitation, and evaporation methods. The formation of the micelles was not observed under any of these conditions, as indicated by the low sizes obtained for *D_v_* and *D_n_*. In contrast, for the third-generation derivative, all described methods consistently gave *Z*-Average values of approximately 20 nm (Figure S52), pointing to direct solution as the most efficient method to prepare these micelles for being the easiest and fastest protocol.

The failure of micelle formation with the second-generation dendrimer could be attributed to the dendritic block not being hydrophobic enough. This has been corroborated after evaluation of the size of the derivatives functionalized with allyl groups. Their increased hydrophobicity hindered their evaluation by DLS by directly dissolving in water, due to the systems being sparingly soluble. However, a comparison of the *Z*-average values obtained after using the evaporation method with those of the acetonide-protected derivatives (Figure 4D) suggested that increasing the hydrophobicity through the presence of allyl functionalities did promote the self-assembly of the systems, even though larger aggregates were also observed in the mixture.

Due to the low polydispersity obtained for the third-generation acetonide-protected derivative (mPEG5k-G3-(Se)_4_(Ac)_4_), the critical micelle concentration (CMC) was quantified by measurements of surface tension in a range of concentrations between 0.01 and 1 mg/mL, obtaining a value of 0.12 mg/mL, what was in the range of previously described systems (Figures 5A and S53).^37^

**Figure 5:**
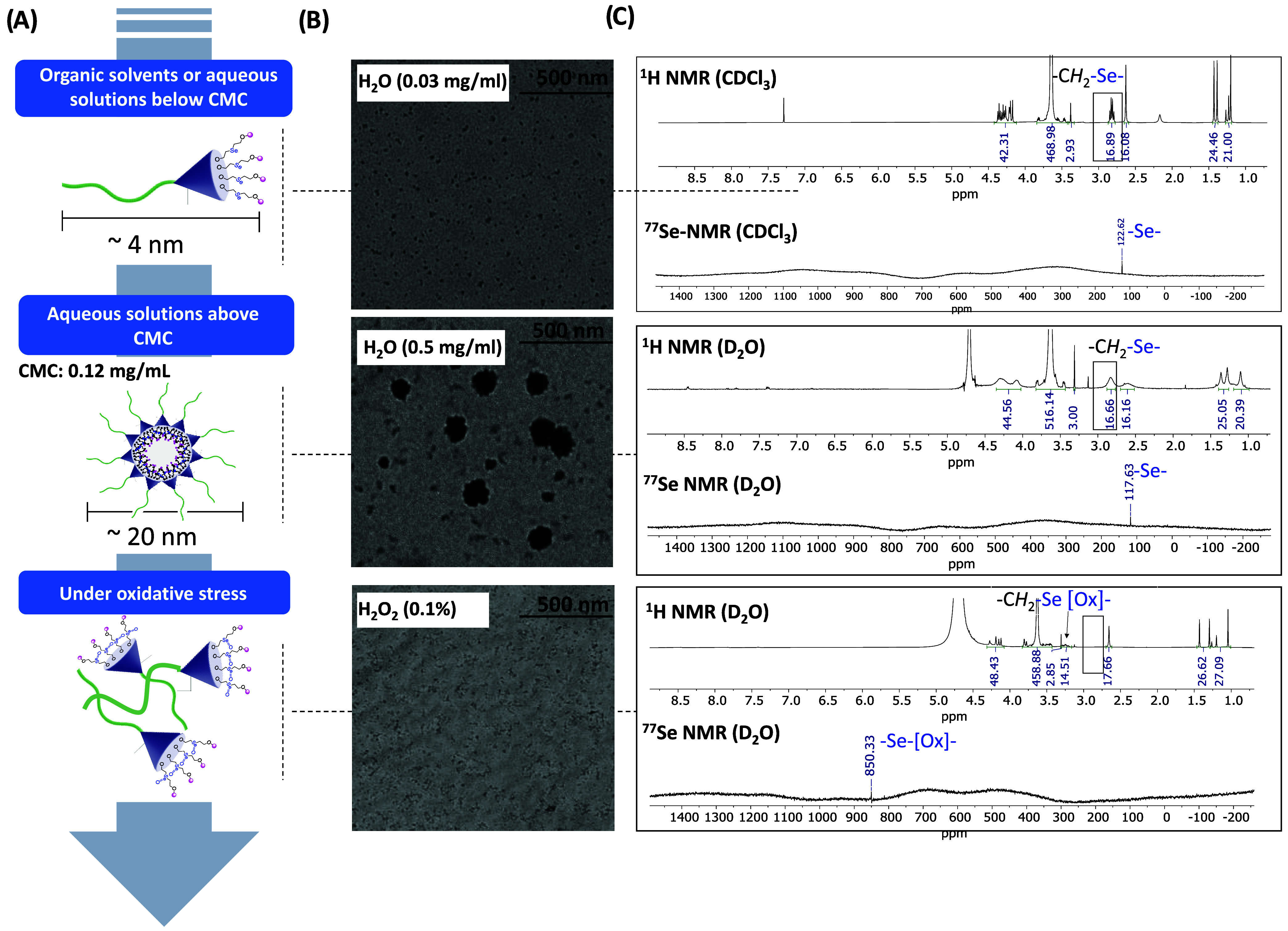
(A) Evolution line starting from a free LD configuration in organic solvents or aqueous solvents below the CMC to their self-assembly into micelles and disassembly under oxidative stress. (B) TEM images of the mPEG5k-G3-(Se)_4_(Ac)_4_ polymer/micelles in water at 0.03 and 0.5 mg/mL as well as after the treatment with H_2_O_2_ (0.1%). (C) Overlay of the ^1^H NMR and ^77^Se NMR spectra under the conditions specified for (B).

The micellar aggregates of mPEG5k-G3-(Se)_4_(Ac)_4_ were spherical, as indicated by transmission electron microscopy (TEM) experiments (Figure 5B). Micellar size measured by TEM was slightly smaller (16.2 nm) than the *Z*-average values obtained by DLS, but almost equal to D_v_ and D_n_. This slight reduction in size might be attributed to the micelle hydration shell in solution as well as the volume shrinkage in the drying process required for the sample preparation in TEM experiments.^41^

Interestingly, despite the compromised stability observed for the monoselenide dendrimers, the micellar constructs formed from mPEG5k-G3-(Se)_4_(Ac)_4_ remained stable up to 1 week under physiological conditions as indicated by DLS measurements (Figure S55). This difference could be attributed to their distinct configurations. Meanwhile, for dendrimers, the selenium moieties face outward, and in micellar arrangements, the Se moieties point inward and might be protected by the presence of PEG chains.

### Controlled Disassembly of Se-Containing Bis-MPA-Based Micelles under Oxidative Stress

The occurrence of Se in the polymer’s backbone allows for exceptional control of the disassembly of micelles under oxidative stress conditions,^42^ which is a particularly attractive feature in drug delivery in cancer treatment where ROS levels are higher compared to normal cells. Hydrogen peroxide (H_2_O_2_) is together with the superoxide anion and hydroxyl radical, a key member of the class of ROS. To understand how Se-dendritic micelles behave under oxidative stress, 0.1% H_2_O_2_ was added into a micellar dispersion of mPEG5k-G3-(Se)_4_(Ac)_4_ (Figure 5A) and its behavior was analyzed by TEM (Figure 5B) as well as ^1^H and ^77^Se NMR (Figure 5C). NMR confirmed that the addition of H_2_O_2_ resulted in the oxidation of the Se moieties, causing a shift of the Se peak in ^77^Se NMR from around 117.6 to 850.3 ppm. In addition to the ^77^Se NMR, the ^1^H NMR spectrum also showed the downfield shift of the signal attributed to the protons of the methylene group next to Se (Figure 5C). Moreover, by this experiment, it is possible to confirm that the oxidation did not alter the C–Se bond or the polyester dendritic skeleton.

As a consequence of the oxidation of the Se atom to selenoxides or selenones, the core of the micelle becomes more hydrophilic, which can lead to its disassembling. This hypothesis is supported by TEM and ^1^H NMR. TEM showed that after 3 h of oxidation, the micelles had completely disassembled, resulting in small particles. In agreement with the TEM, ^1^H NMR showed that after the addition of H_2_O_2_, the signals of the polymer were sharper as a consequence of the disassembly (Figure 5C).

### Preliminary Evaluation of the Impact on Cytotoxicity of Introducing Se into Dendritic Scaffolds

To determine the impact that including Se into bis-MPA biocompatible skeletons has on the cytotoxicity, preliminary cell viability experiments against breast cancer cell lines as well as noncancer fibroblasts were examined after the treatments with the dendrimers and LD polymers by using a CellTiter-Glo luminescent cell viability assay.

No toxicity was observed from the control dendrimers (S_2_-G*n*-(OH)*_m_* or But-G*n*-(OH)*_m_*) on 4T1 (mouse breast cancer cell line) or 3T3 (mouse fibroblasts) at various concentrations up to 40 μM (Figure S56). In contrast, the family of diselenide dendrimers (Se_2_-G*n*-(OH)*_m_*) showed obvious toxicity (Figure 6A). In regard to 4T1 cells, the first-generation diselenide dendrimer was the most potent with an IC_50_ value of 5.6 μM, showing a dose-dependent anticancer effect (Figure 6B). However, for the second- and third-generation counterparts, despite achieving 50% cell viability at a similar concentration to that of the first-generation dendrimer, the cell viability response was not dose-dependent when the concentration was higher than 5 μM. At these levels, further increases in concentration did not significantly impact cell viability, stabilizing at approximately 40% viability at 40 μM for both generations. Therefore, the accurate determination of IC_50_ values was not feasible (Figure 6B). Due to the intriguing results obtained in 4T1 cells for this family of dendrimers, we proceeded with the cytotoxicity test against MCF-7 (human-derived breast cancer cell line) as an additional breast cancer cell line. In this cell line, the three generations of dendrimers showed a dose-dependent effect with highly promising values of IC_50_ between 4.1 and 7.6 μM (Figure 6B). Moreover, when comparing the cytotoxicity of the noncancer cell line 3T3 and the cancerous cell line MCF-7, these dendrimers presented lower values of IC_50_ in cancer cells, indicating a degree of cancer-selective toxicity. It is important to highlight the significant selectivity shown by the Se_2_-G1-(OH)_4_ with an IC_50_ 2 or 1.5 times higher in 3T3 cells compared to 4T1 or MCF-7 cells, respectively.

**Figure 6:**
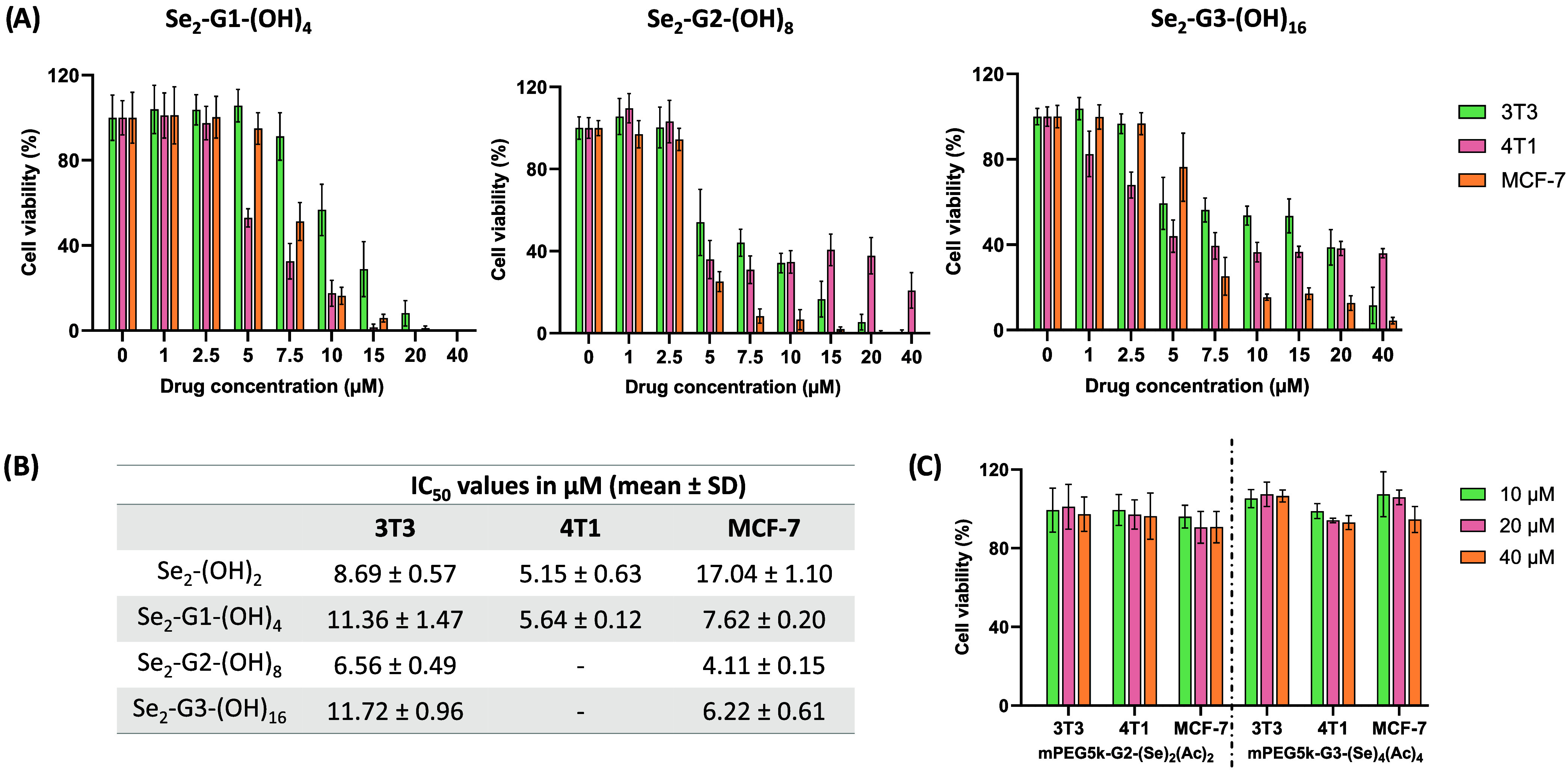
(A) Cell viability percentages of 3T3, 4T1, and MCF-7 cells after 24 h of treatment with diselenide compounds Se_2_-Gn-(OH)*_m_*, where *n* = 1–3 and *m* = 4–16. (B) IC_50_ values for the same family of diselenide dendrimers as well as the 2-hydroxyethyl diselenide core (Se_2_-(OH)_2_). (C) Cell viability percentages of 3T3, 4T1, and MCF-7 cells after 24 h of treatment with acetonide-protected linear dendritic (LD) polymers containing Se (mPEG5k-Gn-(Se)_m_(Ac)*_m_*), where *n* = 2 or 3 and *m* = 2 or 4. Mean values are shown with error bars showing standard deviation, *n* = 3.

To get a better understanding of the role of the bis-MPA moiety on the anticancer effect, the core 2-hydroxyethyl diselenide (Se_2_-(OH)_2_) was also evaluated. The results summarized in Figures 6B and S57 showed that the absence of hydrophobic bis-MPA fragments did not affect the cytotoxicity against 4T1 cells but increased toxicity toward noncancer cells and significantly reduced activity against MCF-7 cells compared to the first-generation counterpart. Such a finding highlights that the balance between hydrophobicity and hydrophilicity is a crucial parameter affecting the anticancer effect.

As a summary, in view of these results, it is worth highlighting the enormous impact of including diselenide bridges in the core of bis-MPA dendrimers compared to hydrophobic chains or disulfide bridges. Despite S and Se belonging to the same group in the periodic table, these results clearly demonstrate the significant impact of choosing one or the other when functionalizing dendritic systems.

As a consequence of the promising performance of the first-generation dendrimer Se_2_-G1-(OH)_4_, this derivative was further functionalized with monodisperse mPEG11 as well as with β-alanine through esterification reactions in order to further analyze how the polarity of the skeleton, the size of the dendritic fragment, and the nature of the peripheral functionalities impact on the biological performance. The results obtained for both post-functionalized dendrimers showed that size as well as the nature of peripheral functionalities played a crucial role in the selectivity toward cancer cells. Regarding the nature of the peripheral functionalities, the cationic derivative, Se_2_-G1-(NH_3_^+^)_4_, with a size similar to that of Se_2_-G1-(OH)_4_, retained some selectivity at specific concentrations (7.5 μM). However, this selectivity diminished at concentrations approaching the IC_50_. This suggests that the nature of the peripheral groups significantly influences the interaction of the dendrimer with the cell membranes. In particular, the introduction of cationic functionalities may alter the hydrophobic–hydrophilic balance of the compound, which is a critical parameter in modulating its biological performance. Moreover, to better understand the specific contribution of size, we designed a PEGylated first-generation dendrimer, Se_2_-G1-(mPEG_11_)_4_, with a molecular weight even higher than that of Se_2_-G3-(OH)_16_, but with much greater hydrophilicity. The fact that the PEGylated dendrimer, despite its polarity, did not show any appreciable selectivity at any tested concentration, with a close IC_50_ value in 3T3 cells (7.07 μM) and 4T1 cells (7.31 μM) (Figure S57), strongly suggests that size is a critical factor limiting selective behavior.

To date, the few papers that report dendrimers containing Se consider these macromolecules as carriers of drugs such as *cis*-platin^24^ or as mimics of glutathione peroxidase^25^ but dendritic Se polymers have yet to be evaluated as anticancer agents. Our study opens new avenues in the search for more selective treatments in the field of nanomedicine where Se plays an important role.

Finally, to understand if the anticancer activity was contributed by the breaking of the diselenide bridge, the first-generation dendrimer decorated with monoselenide in the core (Se-G1-(OH)_4_) was also evaluated. After replacing diselenide with monoselenide, the dendrimer became nontoxic up to 40 μM (Figure S58), confirming the hypothesis of the reduction of the diselenide bond as the main source of the anticancer potency.

All synthesized LDs as precursors of Se-containing micelles were also evaluated in terms of toxicity toward the previously mentioned cell lines. The results summarized in Figures 6C and S59 corroborated what was previously anticipated with Se-G1-(OH)_4_, where no toxicity was observed up to 40 μM indicating low toxicity as was observed for control bis-MPA dendrimers.

## Conclusions

Selenium (Se) has been successfully incorporated into a platform of bis-MPA-based dendritic polymers from monodisperse dendrimers to polydisperse LD hybrids following divergent methodologies. The incorporation of Se has been carried out at the dendritic core as well as at the branches of dendrimers up to third generation, showcasing good compatibility with two of the most robust synthetic approaches involved in the synthesis of polyester dendrimers: anhydride chemistry and FPE chemistry. Although the dendritic core functionalization can be achieved with both diselenide and monoselenide moieties, the functionalization of dendrimers at the branches was only achieved in the monoselenide form due to the well-known metathesis reactions that asymmetric diselenides undergo. Additionally, while diselenide dendrimers showed good stability when stored at −20 °C, monoselenide-based dendrimers resulted in unstable structures after long-term storage, with high hydrophobicity further limiting their potential for biomedical applications. The introduction of monoselenides into LD polymers overcame that drawback due to the presence of PEG, which was a source of hydrophilicity and increased the long-term stability. The third-generation acetonide-protected dendritic polymer mPEG5k-G3-(Se)_4_(Ac)_4_ was capable of self-assembly in aqueous environments, forming spherical micelles of around 20 nm in size according to DLS and TEM measurements, which is a desired size when targeting biomedical applications.

The presence of Se in bis-MPA-based dendritic skeletons has been a source of controlled degradation in both diselenide and monoselenide configurations due to its redox potential. In regard to the presence of Se in dendrimers, diselenide bridges endowed controlled degradation through the reduction of the diselenide functionalities, which was enhanced under intracellular conditions compared to extracellular environments. The presence of monoselenide in the dendritic polymers allowed for the controlled disassembly of their micelles under oxidative stress due to oxidation of the Se atom. Additionally, the presence of ester bonds in these dendritic structures makes them pH-responsive, where an increase in the pH promoted faster degradation through depolymerization mechanisms from outer to inner layers of the dendritic structure.

Finally, from a biological point of view, the choice of the Se linkage is a crucial parameter to take into consideration. Although bis-MPA control dendrimers (But-Gn-(OH)_m_ and S_2_-Gn-(OH)_m_) were not active toward 3T3 or 4T1 cells up to 40 μM, the introduction of diselenide bridges endowed bis-MPA dendritic systems with anticancer activity *per se* leaving external functionalities available for multiple purposes. The size of the dendrimers was an important parameter with a high influence on the selectivity, with the first-generation derivative Se_2_-G1-(OH)_4_ showing promising *in vitro* selectivity for cancer cells. Meanwhile diselenide bridges seemed to be a source of anticancer potential, the presence of monoselenides did not alter the well-known nontoxic nature of bis-MPA skeletons serving as potential future carriers of anticancer drugs which would disassemble under oxidative stress present in cancer cells as shown in TEM and RMN experiments in the presence of H_2_O_2_.

## Supplementary Material



## Data Availability

The raw data used to calculate the results in this manuscript are available in the following public repository: 10.5281/zenodo.14196415.

## Notes

Detailed methods and synthetic protocols together with spectroscopy data (^1^H NMR, ^13^C NMR, ^77^Se NMR, MALDI-ToF, and SEC); MALDI-ToF spectra for the degradation studies; size evaluation by DLS as well as TEM; CMC determination through surface tension measurements; and cell viability for control as post-functionalized dendrimers (PDF)
