# Supplementary material for: Responsive Organoselenium Dendritic Polymers: From Monodisperse Dendrimers to Self-Assembled Micelles for Advanced Therapeutic Applications
Source: J Am Chem Soc. 2025 May 23;147(22):18626–36. doi: 10.1021/jacs.5c00811 (PMC12147156; doi:10.1021/jacs.5c00811)
Supplement: Supplementary file 1 [file ja5c00811_si_001.pdf]

Electronic Supporting Information

Responsive Organoselenium Dendritic Polymers: From Monodisperse Dendrimers to Self-Assembled Micelles for Advanced Therapeutic Applications

Natalia Sanz del Olmo[1]*, Jorge San Jacinto García[1], Yikai Yin[2], Ying Zhao[2], Moustapha Hassan[2], Michael Malkoch[1]*.

1 Department of Fibre and Polymer Technology, KTH Royal Institute of Technology, Teknikringen 56-68, 100 44, Stockholm, Sweden.

2 Department of Laboratory Medicine, Experimental Cancer Medicine (ECM), Karolinska Institute; Center of Allogeneic Stem Cell Transplantation (CAST), Karolinska University Hospital Huddinge, 14186 Stockholm, Sweden.

* Author to whom correspondence should be addressed.

Corresponding authors: Prof. Michael Malkoch and Dr. Natalia Sanz del Olmo

Address correspondence to: School of Engineering Sciences in Chemistry, Biotechnology and Health; Department of Fibre and Polymer Technology; Division of Coating Technology Teknikringen 48, SE-10044, Stockholm. **Fax**: (+) 46 (0)8 790 82 83. **E-mail:** malkoch@kth.se

Table of Contents

Materials and Methods 6

Synthesis of diselenide based polyester dendrimers and control dendrimers with hydrophobic core 9

Synthesis of monoselenide monomers and dendrimers 16

Synthesis of monoselenide linear dendritic (LD) polymers 21

Figures 26

Figure S1. Complex mixture of polyselenides and monoselenides in the synthesis of 2-hydroxyethyl diselenide (**1**) of different batches in $CDCl_3$. 26

Figure S2. ^{1}H, ^{13}C and ^{77}Se NMR of 2-hydroxyethyl diselenide (**1**) in $CDCl_3$. 27

Figure S3. ^{1}H and ^{13}C NMR in $CDCl_3$ of the asymmetric $AB_2^{Se\text{-}Se}$ contaminated with both symmetric derivates after silica chromatography purification. 27

Figure S4. ^{1}H, ^{13}C and ^{77}Se NMR of Se_2-G1-$(Ac)_2$ **(2)** in $CDCl_3$. 28

Figure S5. ^{1}H, ^{13}C and ^{77}Se NMR of Se_2-G1-$(OH)_4$ **(3)** in CD_3OD. 28

Figure S6. ^{1}H, ^{13}C and ^{77}Se NMR of Se_2-G2-$(Ac)_4$ **(4)** in $CDCl_3$ 29

Figure S7. ^{1}H, ^{13}C and ^{77}Se NMR of Se_2-G3-$(Ac)_8$ **(6)** in $CDCl_3$ 29

Figure S8. ^{1}H, ^{13}C and ^{77}Se NMR of Se_2-G3-$(OH)_{16}$ **(7)** in CD_3OD 30

Figure S9. ^{1}H, ^{13}C and ^{77}Se NMR of Se_2-G1-$(mPEG)_4$ **(8)** in CD_3OD. 30

Figure S10. ^{1}H, ^{13}C and ^{77}Se NMR of Se_2-G1-$(NHBoc)_4$ **(9)** in $CDCl_3$. 31

Figure S11. ^{1}H, ^{13}C and ^{77}Se NMR of Se_2-G1-$(NH_3^+)_4$ **(10)** in CD_3OD 31

Figure S12. ^{1}H and ^{13}C NMR of But-G1-$(Ac)_2$ **(11)** in $CDCl_3$ 32

Figure S13. ^{1}H and ^{13}C NMR of But-G1-$(OH)_4$ **(12)** in CD_3OD. 32

Figure S14. ^{1}H and ^{13}C NMR of But-G2-$(Ac)_4$ **(13)** in $CDCl_3$. 33

Figure S15. ^{1}H and ^{13}C NMR of But-G2-$(OH)_8$ **(14)** in CD_3OD 33

Figure S16. ^{1}H and ^{13}C NMR of But-G3-$(Ac)_8$ **(15)** in $CDCl_3$. 34

Figure S17. ^{1}H and ^{13}C NMR of But-G3-$(OH)_{16}$ **(16)** in CD_3OD 34

Figure S18. ^{1}H, ^{13}C and ^{77}Se NMR of 2-hydroxyethyl selenide **(17)** in $CDCl_3$. 35

Figure S19. ^{1}H, ^{13}C and ^{77}Se NMR of 2-((2-hydroxyethyl)selanyl)ethyl 2,2,5-trimethyl-1,3-dioxane-5-carboxylate **(18)** in $CDCl_3$. 35

Figure S20. ^{1}H, ^{13}C and ^{77}Se NMR of 4-oxo-4-(2-((2-((2,2,5-trimethyl-1,3-dioxane-5carbonyl) oxy)ethyl)selanyl) ethoxy) butanoic acid **(19)** in $CDCl_3$. 36

Figure S21. ^{1}H, ^{13}C and ^{77}Se NMR of Se-G1-$(Ac)_2$ **(20)** in $CDCl_3$. 36

Figure S22. ^{1}H, ^{13}C and ^{77}Se NMR of Se-G1-$(OH)_4$ **(21)** in CD_3OD. 37

Figure S23. ^{1}H and ^{13}C NMR of imidazolide activated 4-oxo-4-(2-((2-((2,2,5-trimethyl-1,3-dioxane-5carbonyl) oxy)ethyl)selanyl) ethoxy) butanoic acid. 37

Figure S24. ^{1}H, ^{13}C and ^{77}Se NMR of TMP-G1-$(Se)_3(Ac)_3$ **(22)** in $CDCl_3$. 38

Figure S25. ^{1}H, ^{13}C and ^{77}Se NMR of TMP-G1-$(Se)_3(OH)_6$ **(23)** in $CDCl_3$............................38

Figure S26. ^{1}H, ^{13}C and ^{77}Se NMR of TMP-G2-$(Se)_9(Ac)_6$ **(24)** in $CDCl_3$. 39

Figure S27. ^{1}H, ^{13}C and ^{77}Se NMR of TMP-G2-$(Se)_9(OH)_{12}$ **(25)** in CD_3OD. 39

Figure S28. ^{1}H, ^{13}C and ^{77}Se NMR of TMP-G3-$(Se)_{12}(Ac)_{12}$ **(26)** in $CDCl_3$. 40

Figure S29. ^{1}H and ^{13}C NMR of the anhydride of the 4-oxo-4-(2-((2-((2,2,5-trimethyl-1,3-dioxane-5carbonyl) oxy)ethyl)selanyl) ethoxy) butanoic acid **(27)** in $CDCl_3$ 40

Figure S30. ^{1}H, ^{13}C and ^{77}Se NMR of mPEG(5k)-G2-$(Se)_2(Ac)_2$ **(28)** in $CDCl_3$ 41

Figure S31. ^{1}H, ^{13}C and ^{77}Se NMR of mPEG(5k)-G2-$(Se)_2(OH)_4$ **(29)** in $CDCl_3$ 41

Figure S32. ^{1}H, ^{13}C and ^{77}Se NMR of mPEG(5k)-G3-$(Se)_4(OH)_8$ **(31)** in $CDCl_3$. 42

Figure S33. ^{1}H, ^{13}C and ^{77}Se NMR of mPEG(5k)-G2-$(Se)_2(Allyl)_4$ **(33)** in $CDCl_3$. 42

Figure S34. ^{1}H, ^{13}C and ^{77}Se NMR of mPEG(5k)-G3-$(Se)_4(Allyl)_8$ **(34)** in $CDCl_3$.................43

Figure S35. MALDI-ToF of the dendrimers A) Se_2-Gn-$(Ac)_m$ B) Se_2-Gn-$(OH)_m$. 43

Figure S36. MALDI-ToF of the post functionalized dendrimers: Se_2-G1-$(NHBoc)_4$, Se_2-G1-$(NH_3^+)_4$ and Se_2-G1-$(mPEG_{11})_4$. 44

Figure S37. MALDI-ToF of the dendrimers A) But-Gn-$(Ac)_m$ and B) But-Gn-$(OH)_m$...........44

Figure S38. MALDI-ToF of the dendrimers TMP-Gn-$(Se)_m(Ac)_m$ where n=1-2; m=3-6.......45

Figure S39. SEC analysis of A) Se_2-Gn-$(Ac)_m$ where n=1-3 and m=2-8 and B) Post functionalized first generation diselenide dendrimers...45

Figure S40. SEC analysis of A) But-Gn-$(Ac)_m$ where n=1-3 and m=2-8 and B) But-Gn-$(OH)_m$ where n=1-3 and m=4-16 46

Figure S41. SEC analysis of A) TMP-Gn-$(Se)_m$ $(Ac)_m$ where n=1-3 ; m=3-12 and B) TMP-Gn-$(Se)_{m-3}(OH)_m$ where n=1-2 and m=6-12...46

Figure S42. Stacked MALDI-ToF spectra of the first and second generation monoselenide dendrimers at time zero and after being stored in the freezer (-20 °C) for 6 months. 47

Figure S43. Degradation evaluation through MALDI-ToF of the dendrimers But-G3-$(OH)_{16}$, S_2-G3-$(OH)_{16}$ and Se_2-G3-$(OH)_{16}$ at pH 7.4 and different times. 47

Figure S44. Degradation evaluation through MALDI-ToF of the dendrimers But-G3-$(OH)_{16}$, S_2-G3-$(OH)_{16}$ and Se_2-G3-$(OH)_{16}$ at pH 6.4 and different times. 48

Figure S45. Degradation evaluation through MALDI-TOF of the dendrimers But-G3-$(OH)_{16}$, S_2-G3-$(OH)_{16}$ and Se_2-G3-$(OH)_{16}$ at pH 5.4 and different times. 48

Figure S46. Degradation evaluation through MALDI-TOF of the dendrimers But-G3-$(OH)_{16}$, S_2-G3-$(OH)_{16}$ and Se_2-G3-$(OH)_{16}$ at pH 4.4 and different times. 49

Figure S47. Degradation evaluation through MALDI-TOF of the dendrimers But-G3-$(OH)_{16}$, S_2-G3-$(OH)_{16}$ and Se_2-G3-$(OH)_{16}$ at pH 7.4 and 10 mM of Glutathione at different times.....49

Figure S48. Degradation evaluation through MALDI-TOF of the dendrimers But-G3-$(OH)_{16}$, S_2-G3-$(OH)_{16}$ and Se_2-G3-$(OH)_{16}$ at pH 7.4 and 10 μM of Glutathione at different times......50

Figure S49. Stacked ^{1}H-NMR spectra of the second-generation LD acetonide protected (mPEG5k-G2-$(Se)_2(Ac)_2$) incubated in D_2O at 37°C at various time intervals.........................50

Figure S50. Stacked ^{1}H-NMR spectra of the second (mPEG5k-G2-$(Se)_2(Ac)_2$) and third generation (mPEG5k-G3-$(Se)_4(Ac)_4$) LDs in $CDCl_3$ and D_2O. 51

Figure S51. Hydrodynamic diameters and polydispersity index (PDI) obtained by DLS measurements of the commercially available mPEG5k-OH as well as LD polymers (mPEG5k-G2-$(Se)_2(Ac)_2$ and mPEG5k-G3-$(Se)_4(Ac)_4$) dissolved in PBS at 25 ^{o}C. D_i stands for Hydrodynamic diameter by intensity, D_v Hydrodynamic diameter by volume and D_n Hydrodynamic diameter by number. 52

Figure S52. Hydrodynamic diameters and polydispersity index (PDI) obtained by DLS measurements of the LD polymers (mPEG5k-G2-$(Se)_2(Ac)_2$ and mPEG5k-G3-$(Se)_4(Ac)_4$) formulated at 5 mg/mL under microprecipitation, thin-film and evaporation protocols in PBS at 25 ^{o}C. D_i stands for Hydrodynamic diameter by intensity, D_v Hydrodynamic diameter by volume and D_n Hydrodynamic diameter by number. 52

Figure S53. Surface tension measurements and linear approximation with 95 % confidence to attain the CMC of the third generation (mPEG5k-G3-$(Se)_4(Ac)_4$) at a range of concentrations between 0.001 and 1 mg/mL in PBS at 25 ^{o}C. 53

Figure S54. TEM pictures representative of mPEG5k-G3-$(Se)_4(Ac)_4$ at 0.5 mg/mL in MiliQ water as well as the histogram gathered from 1285 individual micelles from mPEG5k-G3-$(Se)_4(Ac)_4$ at 0.5 mg/mL with a distribution curve overlay and peak value...............................53

Figure S55. Stability study by DLS of the micelle formed from mPEG5k-G3-$(Se)_4(Ac)_4$ incubated at 37 ^{o}C in PBS at 1 mg/mL 53

Figure S56. Cell viability percentages of 3T3 (a) and 4T1 (b) cells after 24h of treatment with a range of concentrations between 1 and 40 μM of CTRL dendrimers (S_2-Gn-$(OH)_m$ where n=1-3 and m=4-16 as well as But-Gn-$(OH)_m$ where n=1-3 and m=4-16). Mean values shown with error bars showing standard deviation, n = 3...54

Figure S57. Cell viability percentages after 24h of treatment with a range of concentrations between 1 and 40 μM of a) the dendritic core (2-hydroxyethyl diselenide, Se_2-$(OH)_2$), b) first generation diselenide dendrimer post-functionalized with $mPEG_{11}$ (Se_2-G1-$(mPEG_{11})_4$) and c) first generation dendrimer functionalized with β-alanine (Se_2-G1-$(NH_3^+)_4$). Mean values shown with error bars showing standard deviation, n ≥ 3. 54

Figure S58. Cell viability percentages of 4T1 and 3T3 cells after 24h of treatment with 10, 20 and 40 μM of Se-G1-$(OH)_4$. Mean values shown with error bars showing standard deviation, n = 3 54

Figure S59. Cell viability percentages of 4T1 and 3T3 cells after 24h of treatment with 10, 20 and 40 μM of the allyl-functionalized linear dendritic (LD) polymers (mPEG5k-Gn-$(Se)_{m/2}(Allyl)_m$ where n=2 or 3 and m=2 or 4). Mean values shown with error bars showing standard deviation, n = 2. 55

References 55

Materials and Methods

Materials. All materials and solvents were purchased from Sigma Aldrich and used as received unless otherwise noted. 2,2-Bis(methylol)propionic acid (bis-MPA) was kindly donated by Perstorp AB, Sweden. Dowex™ 50WX2 50-100 (H) was purchased form Acros Organics, pyridine was purchased from VWR and 1,1'-carbonyldiimidazole (CDI) was purchased from TCI Chemicals. RPMI-1640 and Dulbecco's Modified Eagle Medium (DMEM) cell cultures were obtained from ThermoFisher.

Characterization Methods. *Nuclear Magnetic Resonance (NMR) Spectroscopy.* ^{1}H, ^{13}C and ^{77}Se NMR spectroscopy were performed on a 400 MHz Bruker Avance III. ^{1}H NMR spectroscopy was performed using a 1 s relaxation delay and 20 ppm spectral window. The respective values for ^{13}C NMR spectroscopy were 2 s and 240 ppm, as well as 2 s and 1800 ppm for ^{77}Se NMR. Samples were analyzed in deuterated solvents ($CDCl_3$, CD_3OD and D_2O). ^{1}H-NMR spectra were referenced to the residual solvent peak of $CDCl_3$ δ 7.24, CD_3OD δ 3.31, D_2O δ 4.79 and ^{13}C-NMR spectra to $CDCl_3$ δ 77.0 and CD_3OD δ 49.0. Assignment of the signals has been performed through bidimensional NMR experiments (^{1}H-^{13}C HSQC-2D-NMR and ^{1}H-^{13}C HMBC-2D-NMR) when needed. The spectra were analyzed with MestreNova v. 14.2.0-26256 from Mestrelab Research S.L.

Matrix-Assisted Laser Desorption/Ionization (MALDI-ToF). A Bruker UltrafleXtreme MALDI ToF/ToF Mass Spectrometer (Bruker Daltonics, Bremen, Germany) with a SmartbeamII laser (355 nm, UV) in positive mode was used for the analysis. SpheriCal™ calibrants (Polymer Factory, Sweden) were used for calibrations. The mass spectra were acquired using FlexControl and analyzed with FlexAnalysis Version 3.4 (Bruker Daltonics). For dendrimer characterization, the analyte was dissolved in DCM or MeOH at a concentration of 1 mg mL^{-1}, while, for dendrimer degradation, it was dissolved in phosphate-citrate buffers. The matrix, 2,5-dihydroxybenzoic acid (DHB), was prepared at a concentration of 20 mg mL^{-1} in THF. Samples were prepared by mixing the matrix and analyte at a ratio of 40:1, respectively, and depositing a 5 µL droplet on an MPT 284 Target ground steel TF Target plate purchased from Bruker Daltonics. The spectra were recorded in reflector mode with an acceleration voltage of 25 kV and reflection voltage of 26.3 kV. The laser intensity was adjusted between 50-100% to obtain high resolution spectra.

Size Exclusion Chromatography (SEC). SEC was performed on a TOSOH EcoSECHLC-8320GPC system equipped with an EcoSES RI detector and three columns from PSS GmbH were used (PSS PFG 5 µm; Microguard, 100 Å and 300 Å). The mobile phase was DMF with 0.01M LiBr (0.2mL min^{-1}) at 50 °C using a conventional calibration method with narrow poly (methyl methacrylate) (PMMA) standards for dendrimers and polyethylene glycol (PEG) standards for LDs both purchased from PSS. Corrections for flow rate fluctuations were made using toluene as an internal standard. The data and graphs were processed by WinGPC Unity Software version 7.2, which were normalized and plotted in Origin 2020 (64-bit) SR1 9.7.0.188 (Academic).

Dynamic light scattering (DLS). Samples were analyzed with a Malvern Zetasizer NanoZS at 25 °C in PBS with polymer concentrations of from 1 to 10 mg/mL. Each sample was allowed to equilibrate for 2 minutes at measurement temperature prior to analysis. All results are averages of minimum three individual samples where each sample data is an average of 5 measurements, each consisting of 10 runs. Data was processed using Malvern Zetasizer.

Transmission electron microscopy (TEM). The morphology and size of the nanoparticles were observed under Hitachi TEM HT7700 (Japan), operated at 100kV and equipped with a 2k x 2k camera (AMT XR41, USA) and a W filament. The samples were prepared diluting the compounds at a concentration of 0.03 and 0.5 mg/ml in MiliQ water and placing a 10 µL drop of the solution on a carbon-coated copper TEM grid (EM-Tec Formvar Carbon support film on copper 200 square mesh). The water excess was removed with a tissue and the grid was dried overnight in a desiccator. Images were taken in vacuum without staining in high contrast mode. Particle size was determined analyzing the TEM micrographs with ImageJ, excluding particles with lower diameter than 4 nm.

Surface tension measurements using a tensiometer. The surface tension of mPEG5k-G3-$(Se)_4(Ac)_4$ was determined in a range of concentrations (0.001, 0.002, 0.004, 0.008, 0.016, 0.031, 0.063, 0.125, 0.250, 0.5 and 1 mg/mL) using the Wilhelmy plate method. These measurements were done using PBS as solvent and at 25 ^{o}C. For this experiment a KRÜSS tensiometer has been used. Values calculated for surface tension were then plotted against concentration using Origin 2020 (64-bit) SR1 9.7.0.188 (Academic). Linear fitting was applied to two separate regions with a 95% confidence interval. Intersect was identified for those two regions and determined as CMC for the polymer.

Dendrimer Degradation Evaluation. *pH responsive degradation.* Solutions of But-G3-$[OH]_{16}$, S_2-G3-$[OH]_{16}$ and Se_2-G3-$[OH]_{16}$ dendrimers at 500 µM were prepared in phosphate-citrate buffers of pH 4.4, 5.4, 6.4, and 7.4 with an ionic strength of 0.1 M KCl and kept at 37 °C. Aliquots were analyzed with MALDI by following the above-mentioned protocol at different times (0 h, 1 h, 3 h, 8 h, 24 h, 48 h, 1 week, 2 weeks, 3 weeks and 1 month).

Redox responsive degradation. Solutions of But-G3-$[OH]_{16}$, S_2-G3-$[OH]_{16}$ and Se_2-G3-$[OH]_{16}$ dendrimers at 500 µM were prepared in phosphate-citrate buffers at pH 7.4 containing 10 µM or 10 mM of glutathione (GSH). The solutions were kept at 37 °C and aliquots were analyzed with MALDI by following the above-mentioned protocol at different times depending on the conditions. For 10 µM GSH, the time selected were: 0 h, 1 h, 3 h, 8 h, 24 h, 48 h, 1 week, 2 weeks, 3 weeks and 1 month; whereas for the 10 mM GSH containing buffers the time intervals were: 0 h, 15 min, 30 min, 45 min and 1 h.

Protocols for micelles preparation. *Micro precipitation method.* LD polymer was dissolved in THF (100 µL for 1 mg), then quickly added by micropipette to PBS buffer (filtered through a 0.2 µm syringe filter) in a vial equipped with a magnetic stir bar. The solution was vigorously stirred to achieve the desired concentration. The solution was left to evaporate for 16 hours stirring at 100 RPM, resulting in a clear solution.

Thin film method. LD polymer was dissolved in DCM (100 µL for 1 mg). Afterwards, the DCM was allowed to evaporate slowly under a N_2 flow while the vessel was rotated to create a thin polymeric film. Subsequently high vacuum was applied for 15 minutes ensuring complete removal of DCM. PBS (filtered through a 0.2 µm syringe filter) was added to the vessel to achieve the desired concentration. Afterwards, vigorous vortex for 15 seconds followed by ultrasound bath for 15 minutes was applied resulting in a clear solution.

Evaporation method. LD polymer was dissolved in DMC (100 µL for 1 mg). Afterwards, PBS (filtered through a 0.2 µm syringe filter) was added to the vial along with a small magnetic bar to achieve the desired concentration. The vial was sealed with a lid and allowed to homogenize for 30 minutes at 300 RPM. The lid was removed, the stirring set to 100 RPM and the organic solvent was allowed to slowly evaporate overnight.

Cell culture. Cell experiments were performed using the following cell lines: 4T1 (mouse breast cancer cell), MCF-7 (human breast cancer cell) and 3T3 (mouse embryonic fibroblast cells). All cell lines were purchased from ATCC (American Tissue Culture Collection) and maintained in cell culture containing 10% fetal bovine serum (FBS) and 100 units per mL^{-1} of penicillin as well as 100 µg mL^{-1} streptomycin under 5% CO_2 at 37 °C. Cell culture media used for 4T1 cells was RPMI-1640 medium, whereas 3T3 as well as MCF-7 cells required Dulbecco's Modified Eagle Medium (DMEM).

Cell viability assay. Cytotoxicity of dendritic polymers was determined by CellTiter-Glo® Luminescent Cell Viability Assay (Promega, G7571, WI, USA) according to the manufacturer´s instructions. Briefly, 100 µL of cells were seeded into 96-well plates at a concentration of 5 x 10^4 cells mL^{-1} and cultured for 24h. This was followed by a 24h treatment with dendritic polymers at concentrations ranging from 1µM to 40 µM. Subsequently, 100 µL of CellTiter-Glo® Reagent was added to 100 µL of medium containing cells and mixed for 2 min to induce cell lysis. After 15 min stabilization, luminescence was recorded by Spectramax i3x plate reader (Molecular Devices, CA, USA). Results were analyzed by GraphPad prism 10.1.2 software (GraphPad Inc. La Jolla, CA, USA) and presented as mean ± standard deviation (SD).

Synthetic protocols. Anhydride based monomers such as bis-MPA anhydride[1], mPEG anhydride[2] and β-alanine anhydride[3], were synthesized according to previously published protocols. Similarly, dendrimers with disulfide bonds in the core from first to third generation (S_2-Gn-$[OH]_m$ where n=1-3 and m=4-16)[4], dendritic bis-MPA precursors hydroxyl functionalized (TMP-Gn-$[OH]_m$ where n=1 or 2 and m=6 or 12)[5] as well as LD precursors hydroxyl functionalized (mPEG5k-Gn-$(OH)_m$ where n=1 or 2 and m=2 or 4)[6] were obtained following previously reported literature procedures.

General esterification procedure for the synthesis of dendritic polymers based on anhydride chemistry. Hydroxyl functionalized derivative and 4-(dimethylamino) pyridine (DMAP) were dissolved in pyridine and DCM. Afterwards, the anhydride was added to the solution and left stirring overnight at room temperature. The progress of the reaction was monitored by ^{13}C NMR spectroscopy and MALDI-ToF (in the case of dendrimers).

The purification protocol differed between dendrimers and linear-dendritic (LD) polymers. For dendrimers, after full substitution was reached, the residual anhydride was quenched with water overnight. The solution was thereafter extracted with $NaHSO_4$ (10 %, 3 times) and with Na_2CO_3 (10 %, 3 times). The organic phase was dried on $MgSO_4$, filtered and further purified by column chromatography. The obtained solution is concentrated to dryness. For the purification of the LDs the excess anhydride did not need to be quenched and the polymer was purified by precipitations in cold ether three times followed by drying under high vacuum.

General esterification procedure for the synthesis of dendrimers based on FPE chemistry. CDI was added to a solution of the acid in DCM (1:1 molar ratio). The reaction was carried out for 1 h after which the formation of the imidazolide activated acid was confirmed by ^{1}H and ^{13}C NMR. Afterward, hydroxyl functionalized derivative and CsF were added to the flask and the reaction was carried out with stirring overnight at room temperature. Complete conversion was confirmed with MALDI using DHB as matrix and Cs^+ as counterion (already present in the mixture). The reaction was quenched with water and washed 3 times with 10 % aqueous $NaHSO_4$ and 10 % aqueous $NaHCO_3$, dried with $MgSO_4$, filtered, and concentrated to dryness.

General deprotection procedure for the synthesis of hydroxyl-functional dendritic polymers. The acetonide-protected derivative was dissolved in MeOH and DOWEX™ was added. The mixture was stirred at room temperature and monitored by MALDI-ToF and ^{1}H-NMR. After completion, DOWEX was filtered and the solution is concentrated to dryness.

Synthesis of diselenide based polyester dendrimers and control dendrimers with hydrophobic core

Synthesis of 2-Hydroxyethyl diselenide (1)

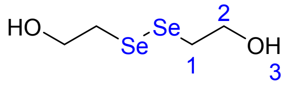

Selenium powder (2 g, 25.3 mmol) was added in small portions to a mixture of KOH (1.4 g, 24.9 mmol), water (7 mL) and 50-60 % hydrazine hydrate (1.1 mL, 14.6 mmol) at 0 °C. The resulting brownish red solution was heated at 85 °C for three hours. After the temperature was cooled to room temperature, 2-bromoethanol (1.8 mL, 26.4 mmol) was dissolved in THF and added dropwise to the mixture which was then left to react for 2 h at 55 °C. After cooling to room temperature, the crude was filtered to remove any traces of unreacted selenium powder, obtaining a yellow solution. The solution was extracted three times with DCM, and the combined organic layer was dried over anhydrous $MgSO_4$. Reddish-orange crude product was obtained after filtering and concentration. After purification by silica gel column chromatography (DCM:EtOAc:EtOH = 47.5:47.5:5), a bright yellowish-orange oil was obtained (1.5 g, 79 %). **^{1}H-NMR** (400 MHz, $CDCl_3$) δ ppm: 3.90 (4H, m, H2), 3.09 (4H, t, *J=6.2 Hz,* H1) and 2.81 ppm (2H, broad singlet, H3). **^{13}C-NMR** (101 MHz, $CDCl_3$) δ ppm: 61.9 (C2), 32.7 (C1). **^{77}Se NMR** (76 MHz, $CDCl_3$) δ ppm: 260.3 (Se-Se).

Synthesis of Se_2-G1-[Ac]$_2$ (2)

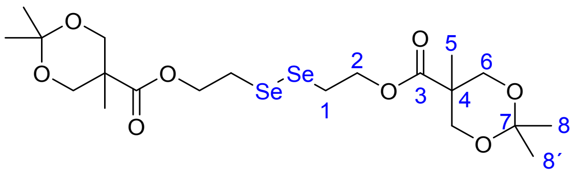

Compound **2** was synthesized following the general esterification procedure for the synthesis of dendrimers based on anhydride chemistry using the following reagents (amounts): 2-hydroxyethyl diselenide **(1)** (1 g, 4.03 mmol), acetonide protected bis-MPA anhydride (4 g, 12.9 mmol), DMAP (100 mg, 0.8 mmol) and pyridine (1 mL). Column chromatography: EtOAc/heptane starting from 20:80 and gradually increasing the polarity. Dendrimer **2** was obtained as a yellow oil (2.2 g, 98 %). **^{1}H NMR** (400 MHz, $CDCl_3$) δ ppm: 4.42 (4H, t, *J=6.4 Hz*, H2), 4.19 (4H, d, *J=11.8 Hz*, H6), 3.64 (4H, d, *J=11.8 Hz*, H6), 3.14 (4H, t, *J=6.4 Hz*, H1), 1.42 (6H, s, H8), 1.38 (6H, s, H8'), 1.19 (6H, s, H5). **^{13}C NMR** (101 MHz, $CDCl_3$) δ ppm: 174.1 (C3), 98.2 (C7), 66.1 (C6), 64.3 (C2), 42.1 (C4), 27.4 (C1), 24.9 (C8), 22.7 (C8'), 18.8 (C5). **^{77}Se NMR** (76 MHz, $CDCl_3$) δ ppm: 268.7 (Se-Se). **MALDI-ToF MS** Calc. 562.06 Da; found $[M+Na]^+$= 585.04 Da. **SEC(DMF)** M_n = 496.6 g/mol, M_w= 506.5 g/mol, Đ = 1.02.

Synthesis of Se_2-G1-$[OH]_4$ (3)

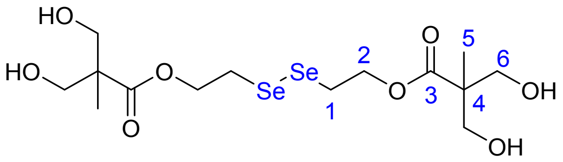

Compound **3** was synthesized following the general deprotection procedure for the synthesis of hydroxyl-functional dendrimers using the following reagents (amounts): Dendrimer **2** (1 g, 1.8 mmol), DOWEX™ (1 g). Dendrimer **3** was obtained as a yellow oil (0.75 g, 87 %). **^{1}H NMR** (400 MHz, CD_3OD) δ ppm: 4.39 (4H, t, *J=6.4 Hz*, H2), 3.67 (8H, m, H6), 3.18 (4H, t, *J=6.4 Hz*, H1), 1.17 (6H, s, H5). **^{13}C NMR** (101 MHz, CD_3OD) δ ppm: 176.3 (C3), 65.8 (C6), 65.2 (C2), 51.6 (C4), 28.2 (C1), 17.4 (C5). **^{77}Se NMR** (76 MHz, CD_3OD) δ ppm: 279.6 (Se-Se). **MALDI-ToF MS** Calc. 482.00 Da; found $[M+Na]^+$= 504.97 Da. **SEC(DMF)** M_n = 532.4 g/mol, M_w= 543.5 g/mol, Đ = 1.02.

Synthesis of Se_2-G2-$[Ac]_4$ (4)

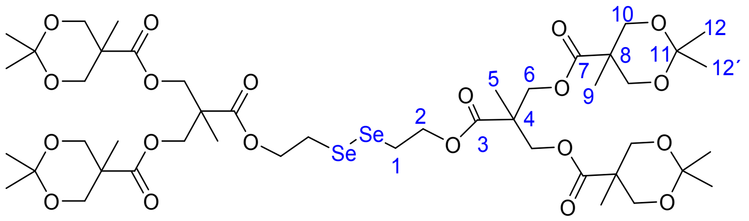

Compound **4** was synthesized following the general esterification procedure for the synthesis of dendrimers based on anhydride chemistry using the following reagents (amounts): Dendrimer **3** (0.73 g, 1.5 mmol), acetonide protected bis-MPA anhydride (3.0 g, 9.1 mmol), DMAP (110 mg, 0.9 mmol) and pyridine (0.7 mL). Column chromatography: EtOAc/heptane starting from 20:80 and increasing slowly the polarity. Dendrimer **4** was obtained as a yellow

oil (1.1 g, 66 %). **^{1}H NMR** (400 MHz, $CDCl_3$) δ ppm: 4.40 (4H, t, *J=6.4 Hz*, H2), 4.33 (8H, s, H6), 4.15 (8H, d, *J=12.2 Hz*, H10), 3.62 (8H, d, *J=12.2 Hz*, H10), 3.11 (4H, t, *J= 6.4 Hz*, H1), 1.41 (12H, s, H12), 1.35 (12H, s, H12'), 1.30 (6H, s, H5), 1.14 (12H, s, H9). **^{13}C NMR** (101 MHz, $CDCl_3$) δ ppm: 173.7 (C7), 172.4 (C3), 98.3 (C11), 66.2 (C10), 65.4 (C6), 64.8 (C2), 47.0 (C8), 42.2 (C4), 27.1 (C1), 25.4 (C12), 22.2 (C12′), 18.7 (C9), 17.9 (C5). **^{77}Se NMR** (76 MHz, CD_3OD) δ ppm: 288.0 (Se-Se). **MALDI-ToF MS** Calc. 1106.31 Da; found $[M+Na]^+$= 1129.35 Da, $[M+K]^+$= 1145.324 Da. **SEC(DMF)** M_n = 1020.8 g/mol, M_w= 1043.9 g/mol, Đ = 1.02.

Synthesis of Se_2-G2-[OH]$_8$ (5)

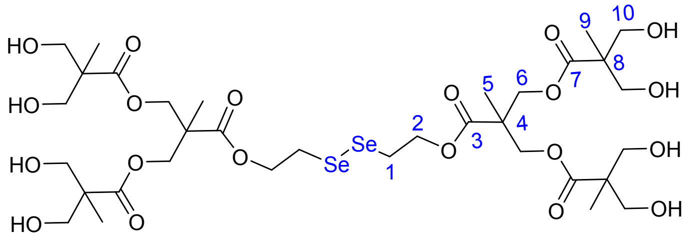

Compound **5** was synthesized following the general deprotection procedure for the synthesis of hydroxyl-functional dendrimers using the following reagents (amounts): Dendrimer **4** (0.6 g, 0.54 mmol), DOWEX™ (0.6 g). Dendrimer **5** was obtained as a yellow oil (0.48 g, 94 %). **^{1}H NMR** (400 MHz, CD_3OD) δ ppm: 4.46 (4H, t, *J= 6.8 Hz*, H2), 4.31 (8H, m, H6), 3.61-3.71 (16H, m, H10), 3.22 (4H, t, *J=6.8 Hz*, H1), 1.33 (6H, s, H5), 1.17 (12H, s, H9). **^{13}C NMR** (101 MHz, CD_3OD) δ ppm: 175.9 (C7), 174.2 (C3), 66.4 (C6), 65.9 (C10), 65.8 (C2), 51.8 (C8), 47.9 (C4), 28.1 (C1), 18.25 (C5), 17.3 (C9). **^{77}Se NMR** (76 MHz, CD_3OD) δ ppm: 280.8 (Se-Se). **MALDI-ToF MS** Calc. 946.19 Da; found $[M+Na]^+$= 969.22 Da, $[M+K]^+$= 985.20 Da. **SEC(DMF)** M_n = 1236.1 g/mol, M_w= 1266.4 g/mol, Đ = 1.02.

Synthesis of Se_2-G3-[Ac]$_8$ (6)

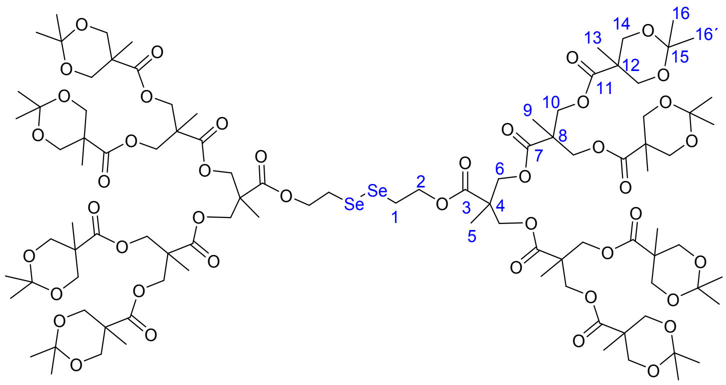

Compound **6** was synthesized following the general esterification procedure for the synthesis of acetonide protected dendrimers based on anhydride chemistry using the following reagents (amounts): Dendrimer **5** (0.33 g, 0.349 mmol), bis-MPA anhydride (1.2 g, 3.6 mmol), DMAP (43 mg, 0.350 mmol) and pyridine (0.3 mL). Column chromatography: EtOAc/heptane starting from 20:80 and increasing slowly the polarity. Dendrimer **5** was obtained as a yellow oil (0.6 g, 78 %). **^{1}H NMR** (400 MHz, $CDCl_3$) δ ppm: 4.41 (4H, t, *J=6.9 Hz,* H2), 4.23-4.34 (24H, s,

H6 and H10), 4.14 (16H, d, *J=12.0 Hz*, H14), 3.61 (16H, d, *J=12.0 Hz*, H14), 3.13 (4H, t, *J=6.9 Hz,* H1), 1.40 (24H, s, H16), 1.34 (24H, s, H16'), 1.26 (18H, s, H5 and H9), 1.13 (24H, s, H13).**^{13}C NMR** (101 MHz, $CDCl_3$) δ ppm: 173.6 (C11), 172.9 (C3,C7), 98.2 (C15), 66.0 (C14), 65.9 (C10), 65.0 (C6), 47.0 (C12), 46.8, (C8), 42.2 (C4), 26.9 (C1), 25.4 (C16), 22.1 (C16´), 18.6 (C13), 17.8 (C5,C9). **^{77}Se NMR** (76 MHz, $CDCl_3$) δ ppm: 268.2 (Se-Se). **MALDI-ToF MS** Calc. 2194.81 Da; found $[M+Na]^{+}$= 2217.79 Da, $[M+K]^{+}$= 2233.94 Da. **SEC(DMF)** M_n = 1987.2 g/mol, M_w= 2010.9 g/mol, Đ = 1.01.

Synthesis of Se_2-G3-$[OH]_{16}$ (7)

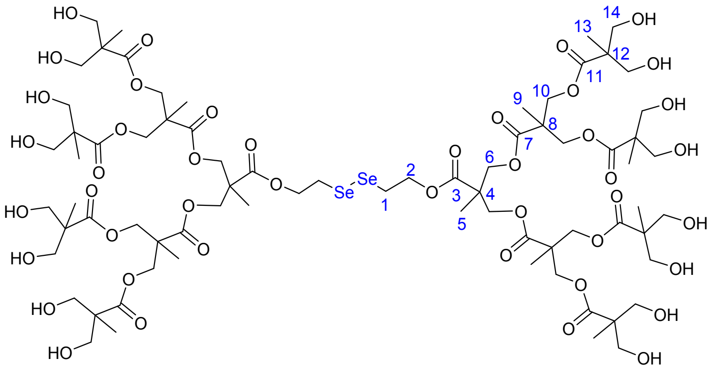

Compound **7** was synthesized following the general deprotection procedure for the synthesis of hydroxyl-functional dendrimers using the following reagents (amounts): Dendrimer **6** (100 mg, 0.046 mmol), DOWEX™ (100 mg). Dendrimer **7** was obtained as a yellow oil (84.3 mg, 98 %). **^{1}H NMR** (400 MHz, CD_3OD) δ ppm: 4.58 (16H, broad s, OH), 4.47 (4H, t, *J= 6.8 Hz,* H2), 4.35 – 4.20 (24H, m, H6 and H10), 3.72 – 3.57 (32H, m, H14), 3.23 (4H, t, *J= 6.8 Hz*, H1), 1.33 (6H, s, H5), 1.30 (12H, s, H9), 1.15 (24H, s, H13). **^{13}C NMR** (101 MHz, CD_3OD) δ ppm: 176.0 (C11), 172.4 (C7), 172.3 (C3), 65.8 (C14), 64.8 (C6, C10), 64.6 (C2), 51.8 (C12), 47.9 (C4, C8), 26.5 (C1), 18.3 (C5, C9), 17.4 (C13). **^{77}Se NMR** (76 MHz, CD_3OD) δ ppm: 281.0 (Se-Se). **MALDI-ToF MS** Calc. 1874.56 Da; found $[M+Na]^{+}$= 1897.779 Da. **SEC(DMF)** M_n = 2615.6 g/mol, M_w= 2653.2 g/mol, Đ = 1.02.

Synthesis of Se_2-G1-$(mPEG11)_4$ (8).

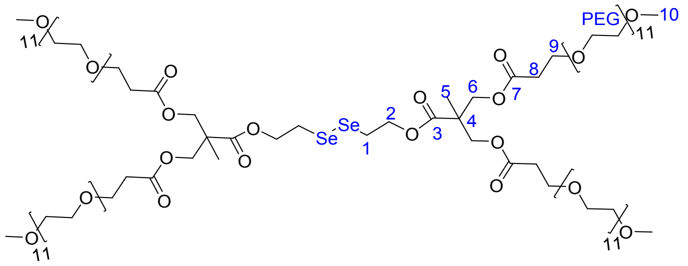

Compound **8** was synthesized following the general esterification procedure based on anhydride chemistry using the following reagents (amounts): dendrimer **3** (35 mg, 0.073 mmol), mPEG11 anhydride (405.5 mg, 0.350 mmol), DMAP (53 mg, 0.434 mmol), pyridine (1 ml). Column chromatography: DCM:MeOH (95:5). Dendrimer **16** was purified obtained as a yellow oil (150 mg, 74.5 %). **^{1}H NMR** (400 MHz, CD_3OD) δ ppm: 4.44 (4H, t, *J= 6.6 Hz*,

H2), 4.28 (8H, s, H6), 3.78-3.51 (184H, m, PEG, H9), 3.36 (12H, s, H10), 3.20 (4H, t, *J=6.6 Hz*, H1), 2.61 (8H, t, *J=6.1 Hz*, H8), 1.29 (6H, s, H5). **^{13}C NMR** (101 MHz, CD_3OD) δ ppm: 173.9 (C3), 172.6 (C7), 72.9-71.3 (PEG), 67.6 (C9), 66.5 (C6), 65.6 (C2), 59.1 (C10), 47.8 (C4), 36.0 (C8), 28.3 (C1), 18.2 (C5). **^{77}Se NMR** (76 MHz, CD_3OD) δ ppm: 279.2 (Se-Se). **MALDI-ToF MS** Calc. 2763.30 Da; found $[M+Na]^+$= 2786.57 Da. **SEC(DMF)** M_n = 3990.4 g/mol, M_w=4073.2 g/mol, Đ =1.02.

Synthesis of Se_2-G1-(NHBoc)$_4$ (9).

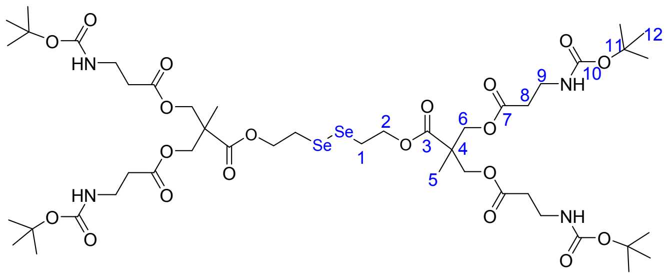

Compound **9** was synthesized following the general esterification procedure based on FPE chemistry using the following reagents (amounts): dendrimer **3** (80 mg, 0.17 mmol), Boc β-alanine (151.3 mg, 0.8 mmol), CDI (129.6 mg, 0.8 mmol), CsF (20 mg, 0.13 mmol). Dendrimer **9** was obtained as a yellow oil (140 mg, 72 %). **^{1}H NMR** (400 MHz, $CDCl_3$) δ ppm: 5.14 (4H, s, NH), 4.41 (4H, t, *J=6.8 Hz*, H2), 4.32-4.16 (8H, m, H6), 3.37 (8H, m, H9), 3.11 (4H, t, *J=6.8 Hz*, H1), 2.53 (8H, t, *J=6.8 Hz*, H8), 1.43 (36H, s, H12), 1.26 (6H, s, H5). **^{13}C NMR** (101 MHz, $CDCl_3$) δ ppm: 172.6 (C7), 172.1 (C3), 155.9 (C10), 79.5 (C11), 65.1 (C6), 64.7 (C2), 46.5 (C4), 36.2 (C9), 34.7 (C8), 28.5(C12), 27.2 (C1), 18.0 (C5). **^{77}Se NMR** (76 MHz, $CDCl_3$) δ ppm: 279.2 (Se-Se). **MALDI-ToF MS** Calc. 1166.35 Da; found $[M+Na]^+$= 1189.45 Da. **SEC(DMF)** M_n = 3990.4 g/mol, M_w= 4076.3 g/mol, Đ = 1.02 .

Synthesis of Se_2-G1-(NH$_3$)$_4$ (10).

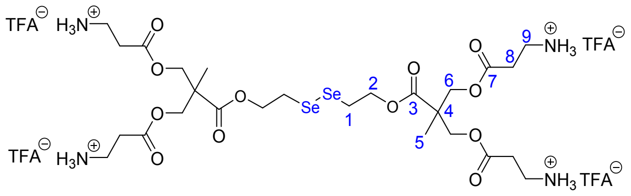

Dendrimer **9** (70 mg, 0.06 mmol) was dissolved in DCM with stirring at room temperature. Trifluoroacetic acid (TFA) was then added carefully (0.2 mL). The vessel was then sealed with a septum equipped with a needle to release the CO_2 generated by the reaction. The reaction mixture was stirred for 1 h and progress was monitored with ^{1}H NMR spectroscopy. Upon completion the solvents were removed and the product was dried *in vacuo* to give dendrimer **10** as a yellow oil (65 mg, 89 %). **^{1}H NMR** (400 MHz, CD_3OD) δ ppm: 4.09 (4H, t, *J= 6.6 Hz*, H2), 3.99 (8H, s, H6), 2.91-2.81 (12H, m, H1 and H9), 2.43 (8H, t, *J= 6.6 Hz*, H8), 0.95 (6H,s, H5). **^{13}C NMR** (101 MHz, CD_3OD) δ ppm: 173.9 (C3), 171.8 (C7), 161.2-160.0 (TFA (C=O)), 121.2-112.6 (TFA (-CH_3)), 67.0 (C6), 65.69 (C2), 47.7 (C4), 36.28 (C9), 32.11 (C8), 28.1 (C1), 18.0 (C5). **^{77}Se NMR** (76 MHz, CD_3OD) δ ppm: 278.8 (Se-Se). **MALDI-ToF MS** Calc. 766.14

Da; found $[M+H]^{+}$= 767.3 Da, $[M+Na]^{+}$= 789.24 Da. **SEC(DMF)** M_n = 3990.4 g/mol, M_w= 4076.3 g/mol, Đ = 1.02.

Synthesis of But-G1-[Ac]$_2$ (11)

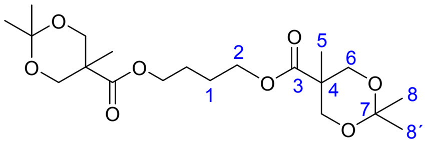

Compound **11** was synthesized following the general esterification procedure for the synthesis of dendrimers using the following reagents (amounts): 1,4- butanediol (1 g, 11.1 mmol), bis-MPA anhydride (10.9 g, 33.2 mmol), DMAP (271 mg, 2.2 mmol) and pyridine (2.7 mL). Dendrimer **11** was obtained as a white solid (3.9 g, 87 %). **^{1}H NMR** (400 MHz, $CDCl_3$) δ ppm: 4.16 (8H, m, H6, H2), 3.62 (4H, d, *J=11.9 Hz*, H6), 1.74 (4H, m, H1), 1.41 (6H, s, H8), 1.36 (6H, s, H8′), 1.16 (6H, s, H5). **^{13}C NMR** (101 MHz, $CDCl_3$) δ ppm: 174.3 (C3), 98.2 (C7), 66.2 (C6), 64.3 (C2), 42.0 (C4), 25.3 (C1), 24.8 (C8), 22.7 (C8'), 18.8 (C5). **SEC(DMF)** M_n = 454.02 g/mol, M_w= 461.69 g/mol, Đ = 1.02.

Synthesis of But-G1-[OH]$_4$ (12)

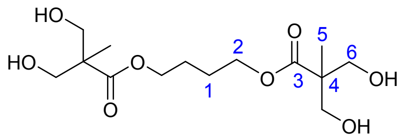

Compound **12** was synthesized following the general deprotection procedure for the synthesis of hydroxyl-functional dendrimers using the following reagents (amounts): Dendrimer **11** (1 g, 2.5 mmol), DOWEX™ (1 g). Dendrimer **12** was obtained as a white solid (0.75 g, 94%). **^{1}H NMR** (400 MHz, CD_3OD) δ ppm: 4.15 (4H, m, H2), 3.66 (8H, m H6), 1.76 (4H, m, H1), 1.16 (6H, s, H5). **^{13}C NMR** (101 MHz, CD_3OD) δ ppm: 176.7 (C3), 65.9 (C6), 65.3 (C2), 51.6 (C4), 26.3 (C1), 17.3 (C5). **SEC(DMF)** M_n = 472.18 g/mol, M_w= 481.58 g/mol, Đ = 1.02.

Synthesis of But-G2-[Ac]$_4$ (13)

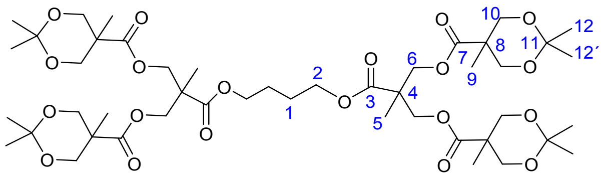

Compound **13** was synthesized following the general esterification procedure for the synthesis of acetonide protected dendrimers using the following reagents (amounts): Dendrimer **12** (0.703 g, 2.18 mmol), bis-MPA anhydride (3.8 g, 11.3 mmol), DMAP (134 mg, 1.1 mmol) and pyridine (0.9 mL). Dendrimer **13** was obtained as a white solid (1.9 g, 92 %). **^{1}H NMR** (400 MHz, $CDCl_3$) δ ppm: 4.32 (8H, s, H6), 4.14 (12H, m, H2, H10), 3.61 (8H, d, *J=12.3 Hz*, H10), 1.70 (4H, m, H1), 1.40 (12H, s, H12), 1.34 (12H, s, H12′), 1.28 (6H, s, H5), 1.13 (12H, s, H9). **^{13}C NMR** (101 MHz, $CDCl_3$) δ ppm: 173.7 (C7), 172.6 (C3), 98.2 (C11), 66.1 (C10), 65.4 (C6), 64.7 (C2), 46.9 (C4), 42.2 (C8), 25.3 (C1), 25.2 (C12), 22.2 (C12'), 18.7 (C9), 17.9 (C5).

MALDI-ToF MS Calc. 946.48 Da; found $[M+Na]^{+}$= 969.56 Da **SEC(DMF)** M_n = 1006.7 g/mol, M_w= 1027.2 g/mol, Đ = 1.02.

Synthesis of But-G2-[OH]$_8$ (14)

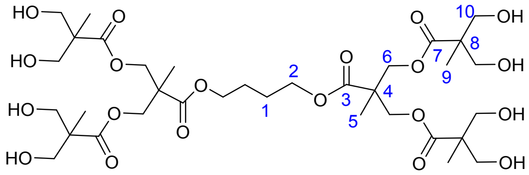

Compound **14** was synthesized following the general deprotection procedure for the synthesis of hydroxyl-functional dendrimers using the following reagents (amounts): Dendrimer **13** (0.5 g, 0.53 mmol), DOWEX™ (0.5 g). Dendrimer **14** was obtained as a white solid (0.35 g, 88%). **^{1}H NMR** (400 MHz, CD_3OD) δ ppm: 4.35 – 4.23 (8H, m, H6), 4.18 (4H, m, H2), 3.71 – 3.55 (16H, m, H10), 1.77 (4H, m, H1), 1.30 (6H, s, H5), 1.15 (12H, s, H9). **^{13}C NMR** (101 MHz, CD_3OD) δ ppm: 175.9 (C7), 174.5(C3), 66.4 (C6), 65.9 (C2), 65.8 (C10), 51.8 (C8), 47.8 (C4), 26.3 (C1), 18.2 (C5), 17.3 (C9). **MALDI-ToF MS** Calc. 786.35 Da; found $[M+Na]^{+}$= 809.32 Da. **SEC(DMF)** M_n = 1142.9 g/mol, M_w= 1165.0 g/mol, Đ = 1.02.

Synthesis of But-G3-[Ac]$_8$ (15)

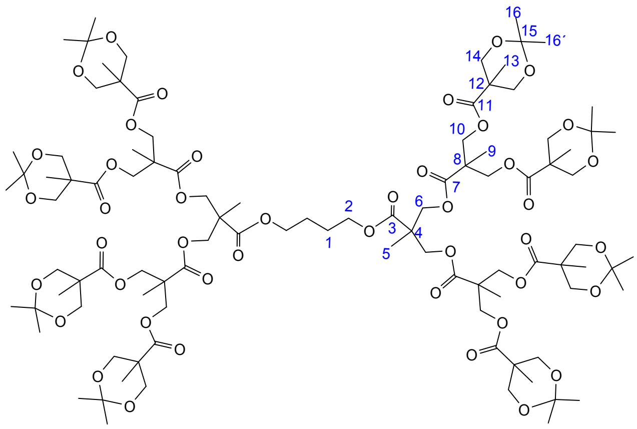

Compound **15** was synthesized following the general esterification procedure for the synthesis of acetonide protected dendrimers using the following reagents (amounts): Dendrimer **14** (0.3 g, 0.381 mmol), bis-MPA anhydride (1.3 g, 3.96 mmol), DMAP (47 mg, 0.381 mmol) and pyridine (0.3 mL). Dendrimer **15** was obtained as a white solid (0.68 g, 85 %). **^{1}H NMR** (400 MHz, $CDCl_3$) δ ppm: 4.30 (12H, s, H10), 4.26 (8H, s, H6), 4.14 (20H, m, H2 and H14), 3.61 (16H, d, *J= 11.9 Hz*, H14), 1.72 (4H, m, H1), 1.40 (24H, s, H16), 1.34 (24H, s, H16′), 1.27 (18H, s, H5 and H9), 1.14 (24H, s, H13). **^{13}C NMR** (101 MHz, $CDCl_3$) δ ppm: 173.6 (C11), 172.1 (C3, C7), 98.2 (C15), 66.1 (14), 65.9 (C6), 65.0 (C10), 47.0 (C8), 46.7 (C4), 42.2 (C12), 25.3 (C16), 25.2 (C1), 22.2 (C16'), 18.7 (C13), 17.9 (C9), 17.8 (C5). **MALDI-ToF MS** Calc. 2034.98 Da; found $[M+Na]^{+}$= 2057.87 Da. **SEC(DMF)** M_n = 1883.8 g/mol, M_w= 1907.8 g/mol, Đ = 1.01.

Synthesis of But-G3-[OH]$_{16}$ (16)

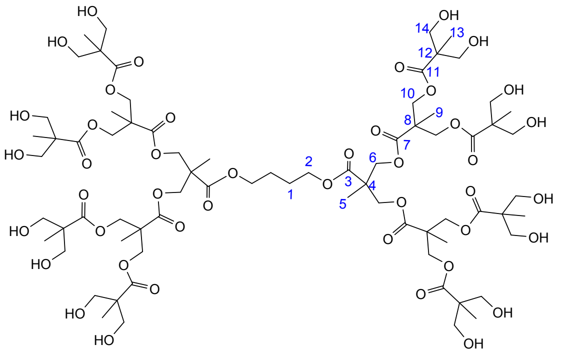

Compound **16** was synthesized following the general deprotection procedure for the synthesis of hydroxyl-functional dendrimers using the following reagents (amounts): Dendrimer **15** (0.3 g, 0.15 mmol), DOWEX^{TM} (0.3 g). Dendrimer **16** was obtained as a white solid (0.23 g, 92 %). **^{1}H NMR** (400 MHz, CD_3OD) δ ppm: 4.42 – 4.19 (28H, m, H2, H6 and H10), 3.72 – 3.54 (32H, m, H14), 1.80 (4H, m, H1), 1.32 (6H, s, H5), 1.30 (12H, s. H9), 1.15 (24H, s, H13). **^{13}C NMR** (101 MHz, CD_3OD) δ ppm: 175.9 (C11), 174.0 (C3), 173.80 (C7), 67.3 (C6), 66.2 (C10), 65.8 (C14), 51.8 (C12), 48.0 (C4, C8), 18.3 (C5, C9), 17.4 (C13). **MALDI-ToF MS** Calc. 1714.73 Da; found $[M+Na]^+$= 1737.68 Da, $[M+K]^+$= 1753.67 Da. **SEC(DMF)** M_n = 2489.7 g/mol, M_w= 2526.0 g/mol, Đ = 1.01.

Synthesis of monoselenide monomers and dendrimers

Synthesis of hydroxyethyl selenide (17)

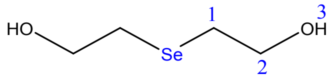

Sodium borohydride (4.6 g, 119 mmol) was dissolved in deionized water (30 mL) at 0 °C and the system was purged with N_2. Then, Se powder (1.5 g, 19 mmol) was added in portions to react for one hour, followed by the addition of 2-bromoethanol (5 g, 0.19 mol) in THF (50 mL) under N_2 flow. The system was stirred at 50 °C overnight and then was extracted with dichloromethane. The organic layers were combined, dried, and concentrated for column chromatography with a 70:30 (Volume ratio) mixture of EtOAc:DCM as eluent. The product was obtained as light-yellow liquid (2.0 g, 63 %). **^{1}H NMR** (400 MHz, $CDCl_3$) δ ppm: 3.82 (4H, t, *J=6.0 Hz,* H2), 2.80 (4H, t, *J=6.0 Hz,* H1), 2.52 (2H, s, H3). **^{13}C NMR** (100 MHz, $CDCl_3$) δ ppm: 61.9 (C2), 27.9 (C1). **^{77}Se NMR** (76 MHz, $CDCl_3$) δ ppm: 76.0 (-Se-).

Synthesis of 2-((2-hydroxyethyl)selanyl)ethyl 2,2,5-trimethyl-1,3-dioxane-5-carboxylate (18)

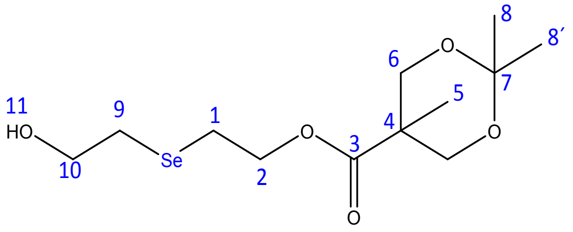

Compound **17** (1.0 g, 5.9 mmol) and 4-(dimethylamino) pyridine (DMAP) (140 mg, 1.2 mmol) were dissolved in pyridine (1.5 mL) and DCM (10 mL). Afterwards, bis-MPA anhydride (1.9 g, 5.9 mmol) was added to the solution and left overnight stirring at room temperature. The progress of the reaction was monitored by ^{1}H and ^{13}C NMR spectroscopy. The solution was thereafter extracted with $NaHSO_4$ (10 %, 3 times) and with Na_2CO_3 (10 %, 3 times). The organic phase was dried on $MgSO_4$, filtered and the solvent was evaporated. The product was purified by column chromatography with a 70:30 (volume ratio) mixture of EtOAc:heptane as eluent. The product was obtained as colourless oil (0.9 g, 50 %). **^{1}H NMR** (400 MHz, $CDCl_3$) δ ppm: 4.36 (2H, t, *J=7.1 Hz*, H2), 4.19 (2H, d, *J=12.0 Hz*, H6), 3.79 (2H, m, H10), 3.64 (2H, d, *J=12.0 Hz,* H6), 2.81 (4H, m, H1 and H9), 2.43 (1H, t, *J= 6.2 Hz*, H11), 1.42 (3H, s, H8), 1.37 (3H, s, H8′), 1.16 (3H, s, H5). **^{13}C NMR** (100 MHz, $CDCl_3$) δ ppm: 174.2 (C3), 98.3 (C7), 66.2 (C6), 64.70 (C2), 61.5 (C10), 42.1 (C4), 28.1 (C9), 25.2 (C8), 22.3 (C8′), 21.5 (C1), 18.8 (C5).**^{77}Se NMR** (76 MHz, $CDCl_3$) δ ppm: 90.8 (-Se-).

Synthesis of 4-oxo-4-(2-((2-((2,2,5-trimethyl-1,3-dioxane-5carbonyl) oxy)ethyl)selanyl) ethoxy) butanoic acid (19)

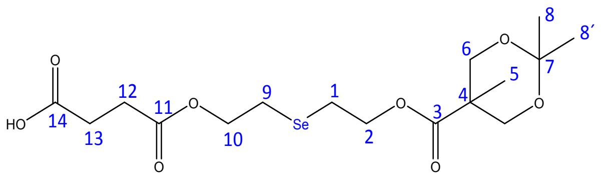

Compound **18** (0.7 g, 2.2 mmol) and 4-(dimethylamino) pyridine (DMAP) (53 mg, 0.4 mmol) were dissolved in pyridine (0.5 mL) and DCM (5 mL). Afterwards, succinic anhydride (258.4 mg, 2.6 mmol) was added to the solution and left overnight stirring at room temperature. The progress of the reaction was monitored by ^{1}H and ^{13}C NMR spectroscopy. The excess of anhydride was quenched with water and the solution was thereafter extracted with $NaHSO_4$ (10 %, 3 times) and with DI water (3 times). The organic phase was dried on $MgSO_4$, filtered and the solvent was evaporated. The product was obtained as colourless oil (0.87 g, 95 %). **^{1}H NMR** (400 MHz, $CDCl_3$) δ ppm: 4.34 (4H, m, H2 and H10), 4.20 (2H, d, *J= 12.0 Hz*, H6), 3.65 (2H, d, *J= 12.0 Hz*, H6), 2.82 (4H, m, H1 and H9), 2.66 (4H, m, H12 and H13), 1.43 (3H, s, H8), 1.39 (3H, s, H8′), 1.19 (3H, s, H5). **^{13}C NMR** (100 MHz, $CDCl_3$) δ ppm:176.3 (C14), 174.2 (C3), 172.0 (C11), 98.4 (C7), 66.1 (C6), 64.7 (C10), 42.1 (C4), 29.1-28.8 (C12, C13), 25.0 (C8), 22.6 (C8′), 21.9 (C1, C9), 18.8 (C5). **^{77}Se NMR** (76 MHz, $CDCl_3$) δ ppm: 124.8 (-Se-).

Synthesis of Se-G1-[Ac]$_2$ (20)

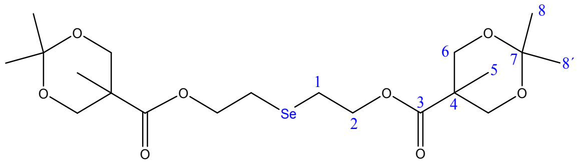

Compound **20** was isolated from the silica column used to purify compound **18**. Dendrimer **20** was obtained as a colourless oil. **^{1}H NMR** (400 MHz, $CDCl_3$) δ ppm: 4.34 (4H, t, *J=7.2 Hz*,

H2), 4.18 (4H, d, *J=11.9 Hz*, H6), 3.63 (4H, d, *J=11.9 Hz*, H6), 2.82 (4H, t, *J=7.2 Hz*, H1), 1.41 (6H, s, H8), 1.37 (6H, s, H8'), 1.18 (6H, s, H5). **^{13}C NMR** (101 MHz, $CDCl_3$) δ ppm: 174.1 (C3), 98.2 (C7), 66.1 (C6), 64.3 (C2), 42.0 (C4), 24.7 (C8), 22.8 (C8'), 21.9 (C1), 18.8 (C5). **^{77}Se NMR** (76 MHz, $CDCl_3$) δ ppm: 120.4 (-Se-).

Synthesis of Se-G1-[OH]$_4$ (21)

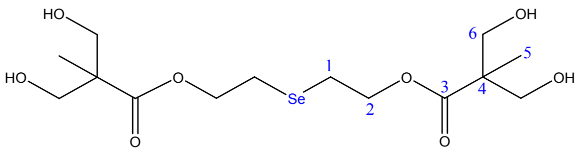

Compound **21** was synthesized following the general deprotection procedure for the synthesis of hydroxyl-functional dendrimers using the following reagents (amounts): Dendrimer **20** (850 mg, 1.8 mmol), DOWEX™ (900 mg). Dendrimer **21** was obtained as a colourless oil (668.1 mg, 94 %). **^{1}H NMR** (400 MHz, CD_3OD) δ ppm: 4.32 (4H, t, *J=7.1 Hz*, H2), 3.67 (8H, m, H6), 2.87 (4H, t, *J=7.1 Hz*, H1), 1.16 (6H, s, H5). **^{13}C NMR** (101 MHz, CD_3OD) δ ppm: 176.3 (C3), 65.8 (C6), 65.5 (C2), 51.6 (C4), 22.4 (C1), 17.3 (C5). **^{77}Se NMR** (76 MHz, CD_3OD) δ ppm: 119.6 (-Se-).

Synthesis of TMP-G1-(Se)$_3$ (Ac)$_3$ (22)

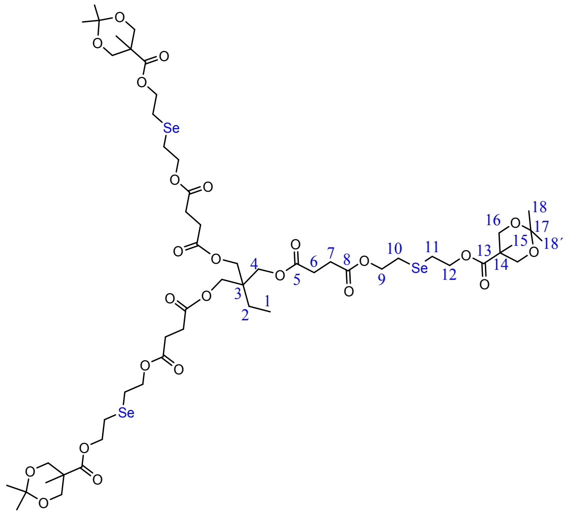

Compound **22** was synthesized following the general esterification procedure for the synthesis of acetonide protected dendrimers based on FPE chemistry using the following reagents (amounts): CDI (217.5 mg, 1.34 mmol), compound **19** (570.6 mg, 1.34 mmol), TMP (50.0 mg, 0.4 mmol) and CsF (34 mg, 0.2 mmol). Compound **22** was obtained as a colourless oil (439 mg, 87 %). **^{1}H NMR** (400 MHz, $CDCl_3$) δ ppm: 4.38-4.24 (12H, m, H9 and H12), 4.18 (6H, d, *J= 11.9 Hz,* H16), 4.03 (6H, s, H4), 3.63 (6H, d, *J= 11.9 Hz,* H16), 2.85-2.76 (12H, m, H10 and H11), 2.63 (12H, m, H6 and H7), 1.47 (2H, m, H2), 1.42 (9H, s, H18), 1.38 (9H, s, H18′), 1.19 (9H, s, H15), 0.88 (3H, m, H1). **^{13}C NMR** (100 MHz, $CDCl_3$) δ ppm: 174.1(C13), 172.0 (C5,C8), 98.2 (C17), 66.1 (C16), 64.5 (C4), 64.4 (C12), 64.1 (C9), 42.1 (C14), 40.9 (C3), 29.1 (C6, C7), 24.7 (C18), 23.1 (C2), 22.8 (C18′), 22.0 (C10, C11), 18.8 (C15), 7.50 (C1). **^{77}Se**

NMR (76 MHz, $CDCl_3$) δ ppm: 122.7 (-Se-). **MALDI-ToF MS** Calc. 1358.30 Da; found $[M+Na]^+$= 1381.34 Da. **SEC(DMF)** M_n = 1396 g/mol, M_w= 1424 g/mol, Đ = 1.02.

Synthesis of TMP-G1-$(Se)_3(OH)_6$ (23)

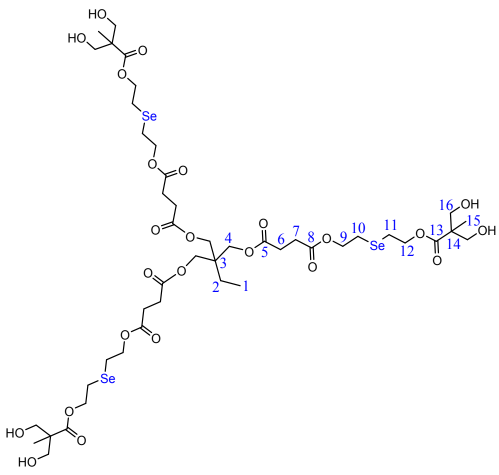

Compound **23** was synthesized following the general deprotection procedure for the synthesis of hydroxyl-functional dendrimers using the following reagents (amounts): Dendrimer **22** (250 mg, 0.18 mmol), DOWEX™ (250 mg). Dendrimer **23** was obtained as a light-yellow oil (210.9 g, 93 %). **^{1}H NMR** (400 MHz, CD_3OD) δ ppm: 4.43-4.26 (12H, m, H9 and H12), 4.04 (6H, s, H4), 3.87 (6H, d, *J= 11.2 Hz*, H16), 3.72 (6H, d, *J= 11.2 Hz*, H16), 2.91-2.76 (12H, m, H10 and H11), 2.64 (12H, s, H6 and H7), 1.47 (2H, m, H2), 1.08 (9H, s, H15), 0.88 (3H, m, H1). **^{13}C NMR** (101 MHz, CD_3OD) δ ppm: 175.7 (C13), 172.2-172.1 (C5, C8), 68.0 (C16), 64.6-64.1 (C4, C9, C12), 49.5 (C14), 40.9 (C3), 29.1 (C6, C7), 23.1 (C2), 22.2-21.8 (C10, C11), 17.3 (C15), 7.5 (C1). **^{77}Se NMR** (76 MHz, CD_3OD) δ ppm: 121.2 (-Se-). **MALDI-ToF MS** Calc. 1238.21 Da; found $[M+Na]^+$= 1261.12 Da. **SEC(DMF)** M_n = 1862 g/mol, M_w= 1897 g/mol, Đ = 1.02.

Synthesis of TMP-G2-$(Se)_9(Ac)_6$ (24)

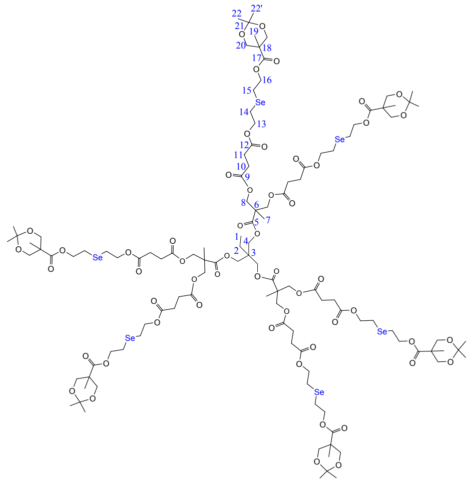

Compound **24** was synthesized following the general esterification procedure for the synthesis of acetonide protected dendrimers based on FPE chemistry using the following reagents

(amounts): CDI (282.0 mg, 1.74 mmol), compound **19** (740.0 mg, 1.74 mmol), TMP-G1-$(OH)_6$ (100 mg, 0.21 mmol) and CsF (38.2 mg, 0.25 mmol). Compound **24** was obtained as a colourless oil (436 mg, 72 %). **^{1}H NMR** (400 MHz, $CDCl_3$) δ ppm: 4.37-4.24 (24H, m, H13 and H16), 4.21 (12H, s, H8), 4.16 (12H, d, *J=11.9 Hz*, H20), 4.03 (6H, s, H4), 3.62 (12H, d, *J=11.9 Hz*, H20), 2.84-2.73 (24H, m, H14 and H15), 2.60 (24H, s, H10 and H11), 1.44 (2H, m, H2), 1.40 (18H, s, H22), 1.36 (18H, s, H22'), 1.22 (9H, s, H7), 1.17 (18H, s, H19), 0.90 (3H, m, H1). **^{13}C NMR** (100 MHz, $CDCl_3$) δ ppm: 174.0 (C17), 172.2 (C5), 171.9-171.7 (C9, C12), 98.2 (C21), 66.0 (C20), 65.4-63.8 (C4, C8, C13, C16), 46.6 (C18), 42.0 (C6), 41.6 (C3), 28.9 (C10, C11), 24.7 (C22), 22.8 (C22′), 21.9-21.8 (C14, C15), 18.7 (C19), 17.9 (C7), 7.5 (C1). **^{77}Se NMR** (76 MHz, $CDCl_3$) δ ppm: 122.5 (-Se-). **MALDI-ToF MS** Calc. 2930.65 Da; found $[M+Na]^+$ = 2953.04 Da. **SEC(DMF)** M_n = 2678 g/mol, M_w= 2795 g/mol, Đ = 1.04.

Synthesis of TMP-G2-$(Se)_9(OH)_{12}$ (25)

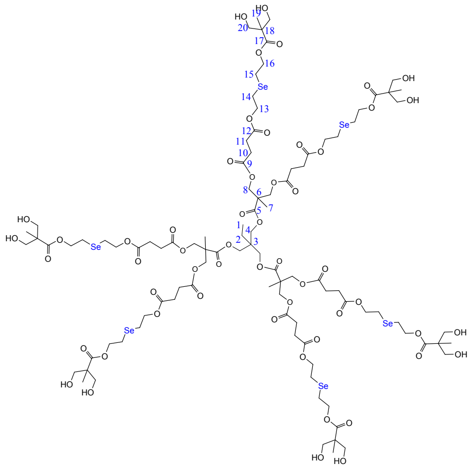

Compound **25** was synthesized following the general deprotection procedure for the synthesis of hydroxyl-functional dendrimers using the following reagents (amounts): Dendrimer **24** (400 mg, 0.14 mmol), DOWEX^{TM} (400 mg). Dendrimer **25** was obtained as a light-yellow oil (323.1 g, 88%). **^{1}H NMR** (400 MHz, CD_3OD) δ ppm: 4.37-4.29 (24H, m, H13 and H16), 4.27 (12H, s, H8), 4.15 (6H, s, H4), 3.73-3.60 (24H, m, H20), 2.91-2.82 (24H, m, H14 and H15), 2.65 (24H, s, H10 and H11), 1.56 (2H, m, H2), 1.28 (9H, s, H7), 1.17 (18H, s, H19), 0.99 (3H, m , H1). **^{13}C NMR** (101 MHz, CD_3OD) δ ppm: 176.4 (C17), 173.8-173.4 (C5, C9, C12), 66.8 (C8), 65.8-65.5 (C4, C13, C16, C20), 51.6 (C18), 47.9 (C3, C6), 29.9 (C10, C11), 22.7-22.6 (C14, C15), 18.3 (C7), 17.4 (C19). **^{77}Se NMR** (76 MHz, CD_3OD) δ ppm: 121.3 (-Se-). **MALDI-ToF MS** Calc. 2690.46 Da; found $[M+Na]^+$= 2713.65 Da. **SEC(DMF)** M_n = 3837 g/mol, M_w= 4129 g/mol, Đ = 1.07.

Synthesis of TMP-G3-$(Se)_{12}(Ac)_{12}$ (26)

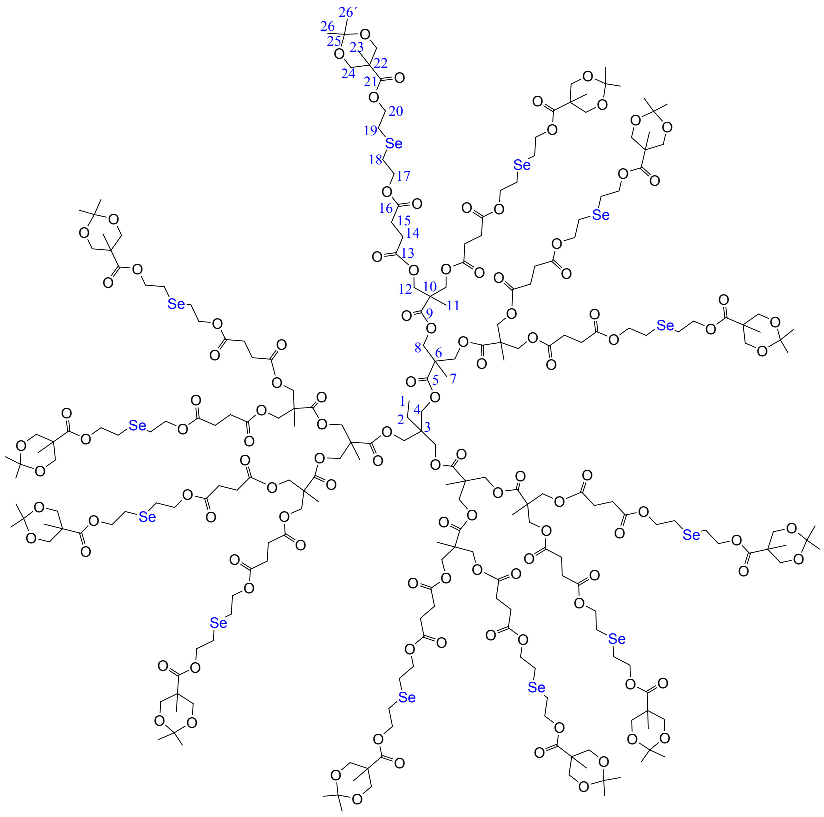

Compound **26** was synthesized following the general esterification procedure for the synthesis of acetonide protected dendrimers based on FPE chemistry using the following reagents (amounts): CDI (49.5 mg, 0.3 mmol), compound **19** (129.8 mg, 0.3 mmol), TMP-G2-$(OH)_{12}$ (20 mg, 0.02 mmol) and CsF (6 mg, 0.04 mmol). Compound **26** was obtained as a light-yellow oil (97.3 mg, 80 %). **^{1}H NMR** (400 MHz, $CDCl_3$) δ ppm: 4.38-4.10 (114H, m, H4, H8, H12, H17, H20 and H24), 3.63 (24H, d, H24), 2.86-2.84 (48H, m, H18 and H19), 2.61 (48H, s, H14 and H15), 1.41 (36H, s, H26), 1.37 (36H, s, H26'), 1.26 (9H, s, H7), 1.21 (18H, s, H11), 1.18(36H, s, H23), 0.93 (3H, m, H1). **^{13}C NMR** (100 MHz, $CDCl_3$) δ ppm: 174.1-171.8 (C5, C9, C13, C16, C21), 98.2 (C25), 66.1 (C24), 65.3-64.3 (C4, C8, C12, C17, C20), 46.9- 42.0 (C3, C6, C10, C22), 28.9 (C14, C15), 24.7 (C26), 22.8 (C26'), 21.9 (C18, C19), 18.8-17.9 (C7, C11, C23). **^{77}Se NMR** (76 MHz, $CDCl_3$) δ ppm: 122.2 (-Se-). **SEC(DMF)** M_n = 4056 g/mol, M_w= 4162 g/mol, Đ = 1.02.

Synthesis of monoselenide linear dendritic (LD) polymers

Synthesis of Se-containing bis-MPA anhydride (27).

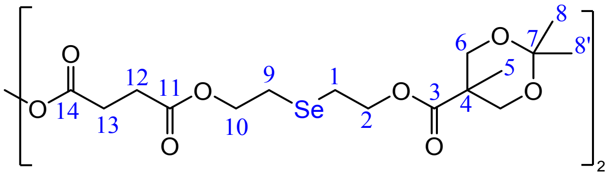

Compound **17** (4 g, 9.4 mmol) was dissolved in DCM with stirring. The reaction vessel was subsequently cooled in an ice bath. DCC (0.97 g, 4.7 mmol) dissolved in DCM was added

dropwise. Once addition was completed, the reaction was allowed to proceed overnight at room temperature. The reaction mixture was filtered and the filtrate was collected and evaporated by rotary evaporation and the product dried *in vacuo* to give the Se- containing bis-MPA anhydride **(27)** as a colourless oil (3.7 g, 95 %).^{1}**H NMR** (400 MHz, $CDCl_3$) δ ppm: 4.32 (8H, m, H2 and H10), 4.16 (4H, d, *J= 11.9 Hz*, H6), 3.62 (4H, d, *J= 11.9 Hz*, H6), 2.80 (8H, m, H1 and H9), 2.67 (8H, m, H12 and H13), 1.41 (6H, s, H8), 1.36 (6H, s, H8′), 1.17 (6H, s, H5). ^{13}**C NMR** (100 MHz, $CDCl_3$) δ ppm: 174.1 (C3), 171.4 (C11), 167.9 (C14), 98.2 (C7), 66.1 (C6), 64.6 (C2, C10), 42.0 (C4), 30.3-28.5 (C12, C13), 24.8 (C8), 22.7 (C8′), 21.8 (C1, C9), 18.7 (C5).

Synthesis of mPEG(5k)-G2-(Se)$_2$(Ac)$_2$ (28).

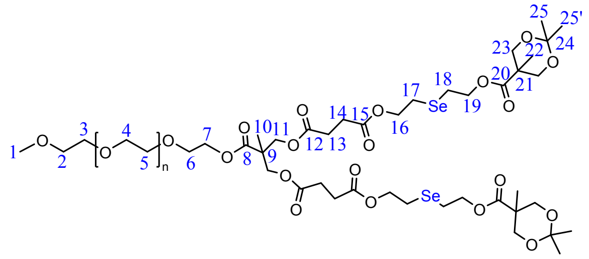

Compound **28** was synthesized following the general esterification procedure based on anhydride chemistry using the following reagents (amounts): mPEG(5k)-G1-(OH)$_2$ (1.3 g, 0.26 mmol), monoselenide anhydride **(27)** (0.9 g, 1.0 mmol), DMAP (13 mg, 0.1 mmol), pyridine (0.1 ml). Polymer **28** was purified by precipitations in cold ether three times obtaining a white solid (1.3 g, 84 %). ^{1}**H NMR** (400 MHz, $CDCl_3$) δ ppm: 4.40-4.16 (22H, m, H7, H11, H16, H19 and H23), 3.64 (452H, s, PEG), 3.37 (3H, s, H1), 2.82 (8H, m, H17, H18), 2.62 (8H, s, H13, H14), 1.42 (6H, s, H25), 1.38 (6H, s, H25'), 1.24 (3H, s, H10), 1.19 (6H, s, H22). ^{13}**C NMR** (101 MHz, $CDCl_3$) δ ppm: 174.0-171.7 (C8, C12, C15 and C20), 98.2 (C24), 70.7 (PEG), 66.1 (C23), 65.5 (C11), 64.3 (C16, C19), 59.1 (C1), 46.4 (C21), 42.0 (C9), 29.0 (C13, C14), 24.7 (C25), 22.8 (C25′), 21.9-21.8 (C17, C18), 18.7 (C22), 17.8 (C10). ^{77}**Se NMR** (76 MHz, $CDCl_3$) δ ppm: 122.6 (-Se-). **SEC(DMF)** M_n = 5134 g/mol, M_w= 5771 g/mol, Đ = 1.1.

Synthesis of mPEG(5k)-G2-(Se)$_2$(OH)$_4$ (29).

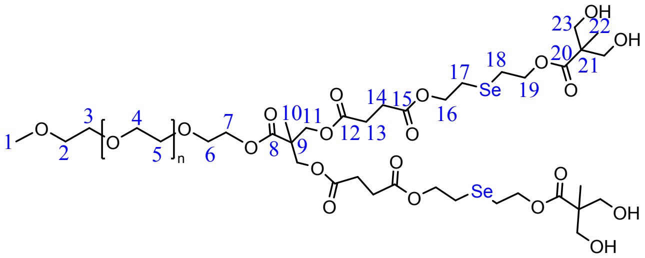

Compound **29** was synthesized following the general deprotection procedure for the synthesis of hydroxyl-functional dendritic polymers using the following reagents (amounts): Polymer **28** (200 mg, 0.03 mmol), DOWEX^{TM} (200 mg). Polymer **29** was obtained as a white solid (150 mg, 76%). %). ^{1}**H NMR** (400 MHz, $CDCl_3$) δ ppm: 4.40-4.19 (14H, m, H7, H11, H16, H19), 3.64 (460H, m, PEG and H23), 3.37 (3H, s, H1), 2.89-2.77 (8H, m, H17, H18), 2.62 (8H, s,

H13, H14), 1.24 (3H, s, H10), 1.09 (6H, s, H22). **^{13}C NMR** (101 MHz, $CDCl_3$) δ ppm: 175.3.0-171.6 (C8, C12, C15 and C20), 70.5 (PEG), 68.8-63.8 (C11, C16, C19 and C23), 58.9 (C1), 49.4 (C9), 46.2 (C21), 28.8 (C13, C14), 21.9-21.6 (C17, C18), 17.7 (C10), 17.2 (C22). **^{77}Se NMR** (76 MHz, $CDCl_3$) δ ppm: 122.2 (-Se-). **SEC(DMF)** M_n = 5951 g/mol, M_w= 6940 g/mol, Đ = 1.2.

Synthesis of mPEG(5k)-G3-(Se)$_4$(Ac)$_4$ (30).

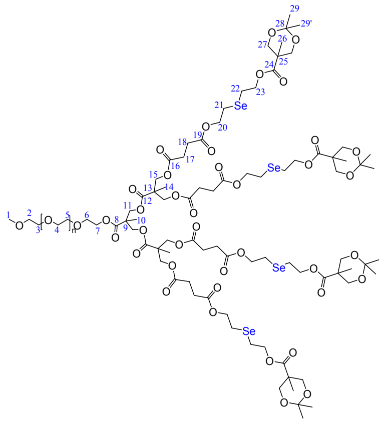

Compound **30** was synthesized following the general esterification procedure based on anhydride chemistry using the following reagents (amounts): mPEG(5k)-G2-(OH)$_4$ (1.3 g, 0.25 mmol), monoselenide anhydride **(27)** (1.7 mg, 2.0 mmol), DMAP (24 mg, 0.2 mmol), pyridine (0.1 ml). Polymer **30** was purified by precipitations in cold ether three times obtaining a white solid (1.5 g, 88 %). **^{1}H NMR** (400 MHz, $CDCl_3$) δ ppm: 4.36-4.12 (42H, m, H7, H15, H20, H23 and H27), 3.62 (452H, s, PEG), 3.36 (3H, s, H1), 2.79 (16H, m, H21 and H22), 2.60 (16H, m, H17 and H18), 1.40 (12H, s, H29), 1.36 (12H, s, H29'), 1.24 (3H, s, H10), 1.20 (6H, s, H14), 1.18 (12H, s, H26). **^{13}C NMR** (101 MHz, $CDCl_3$) δ ppm: 174.1- 171.7 (C8, C12, C16, C19 and C24), 98.2 (C28), 70.7 (PEG), 66.1 (C27), 65.4 (C11 and C15), 64.4 (C20 and C23) 59.1 (C1), 46.7 (C25), 42.0 (C9 and C13), 28.9 (C17 and C18), 24.7 (C21 and C22), 22.8 (C29), 21.9 (C29'), 18.8 (C26), 17.8 (C14), 17.7 (C10). **^{77}Se NMR** (76 MHz, $CDCl_3$) δ ppm: 122.5 (-Se-). **SEC(DMF)** M_n = 5822 g/mol, M_w= 6430 g/mol, Đ = 1.1.

Synthesis of mPEG(5k)-G3-(Se)$_4$(OH)$_8$ (31).

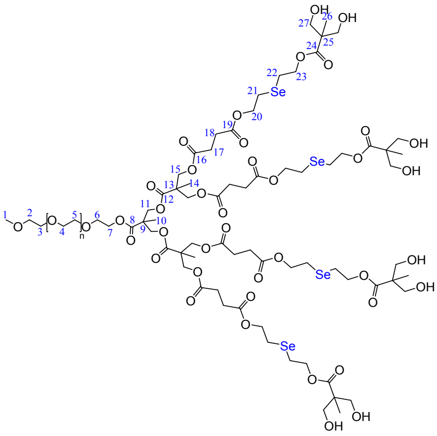

Compound **31** was synthesized following the general deprotection procedure for the synthesis of hydroxyl-functional dendritic polymers using the following reagents (amounts): Polymer **30** (200 mg, 0.03 mmol), DOWEX^{TM} (200 mg). Polymer **31** was obtained as a white solid (198 mg, 97%). **^{1}H NMR** (400 MHz, $CDCl_3$) δ ppm: 4.40-4.16 (26H, m, H7, H15, H20, H23), 3.62 (468H, s, PEG and H27), 3.37 (3H, s, H1), 2.89-2.77 (16H, m, H21 and H22), 2.62 (16H, m, H17 and H18), 1.26 (3H, s, H10), 1.22 (6H, s, H14), 1.09 (12H, s, H26). **^{13}C NMR** (101 MHz, $CDCl_3$) δ ppm: 175.6- 171.9 (C8, C12, C16, C19 and C24), 70.7 (PEG), 67.8-64.1 (C7, C11, C15, C20, C23 and C27), 59.2 (C1), 49.6 (C9 and C13), 46.5 (C25), 29.0 (C17 and C18), 22.2-21.8 (C21 and C22), 17.9-17.3 (C10, C14 and C26). **^{77}Se NMR** (76 MHz, $CDCl_3$) δ ppm: 122.3 (-Se-). **SEC(DMF)** M_n = 6782 g/mol, M_w= 7874 g/mol, Đ = 1.2.

Synthesis of 4-pentenoic anhydride (32).

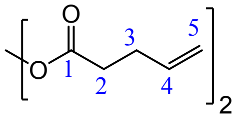

4-Pentenoic acid (20 g, 0.20 mol) was dissolved in DCM with stirring. The reaction vessel was subsequently cooled in an ice bath. DCC (20.60 g, 0.10 mol) dissolved in DCM was added dropwise. Once addition was completed, the reaction was allowed to proceed overnight at room temperature. The reaction mixture was filtered and the filtrate was collected and evaporated by rotary evaporation and the product dried *in vacuo* to give the 4-pentenoic anhydride as a colourless oil (18.5 g, 87 %). ^{1}H NMR (400 MHz, $CDCl_3$) δ 5.81 (2H, ddt, *J= 16.8, 10.2, 6.4* Hz, H4), 5.12 – 5.01 (4H, m, H5), 2.54 (4H, td, *J= 7.6, 0.9* Hz, H2), 2.39 (4H, dtt*, J= 7.6, 6.4, 1.5* Hz, H3). ^{13}C NMR (101 MHz, $CDCl_3$) δ 168.67 (C1), 135.72 (C4), 116.15 (C5), 34.52 (C2), 28.09 (C3).

Synthesis of mPEG(5k)-G2-$(Se)_2(Allyl)_4$ (33).

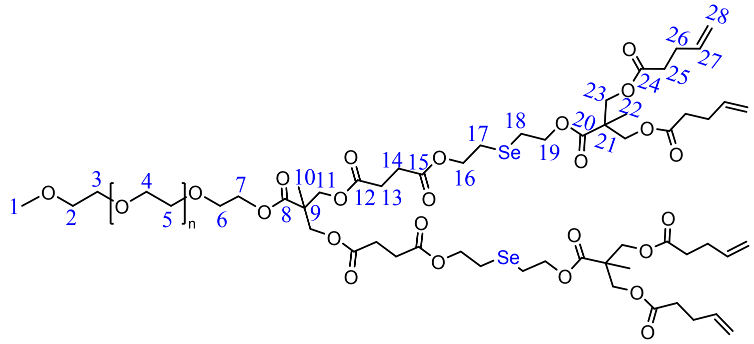

Compound **33** was synthesized following the general esterification procedure based on anhydride chemistry using the following reagents (amounts): mPEG(5k)-G2-$(Se)_2(OH)_4$ **(29)** (200 mg, 0.03 mmol), pentenoic anhydride (52 mg, 0.27 mmol), DMAP (3 mg, 0.002 mmol), pyridine (1 ml). Polymer **32** was purified by precipitations in cold ether three times obtaining a white solid (185 mg, 88 %).**^{1}H NMR** (400 MHz, $CDCl_3$) δ ppm: 5.78 (4H, m, H27), 5.01 (8H, m, H28), 4.35-4.19 (22H, m, H7, H11, H16, H19 and H23), 3.63 (452H, m, PEG), 3.37 (3H, s, H1), 2.79 (8H, m, H17 and H18), 2.62 (8H, s, H13 and H14), 2.44-2.31 (16H, m, H25 and H26), 1.25-1.23 (9H, s, H10 and H22). **^{13}C NMR** (101 MHz, $CDCl_3$) δ ppm: 172.6-171.6

(C8, C12, C15, C20 and C24), 136.6 8 (C27), 115.8 (C28), 70.7 (PEG), 69.0-63.9 (C7, C11, C16, C19, C23), 59.1 (C1), 46.4 (C9 and C21), 33.5 (C25), 31.0 (C26), 28.8 (C13 and C14), 21.9 (C17 and C18), 18.0 (C10), 15.4 (C22). **^{77}Se NMR** (76 MHz, $CDCl_3$) δ ppm: 122.7 (-Se-). **SEC(DMF)** M_n = 5957 g/mol, M_w= 6763 g/mol, Đ = 1.1.

Synthesis of mPEG(5k)-G3-(Se)$_4$(Allyl)$_8$ (34).

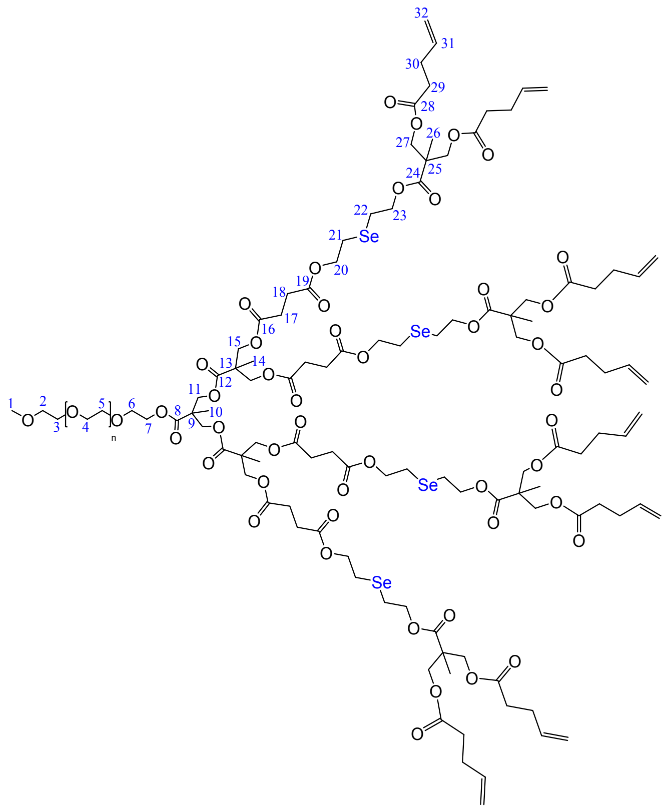

Compound **34** was synthesized following the general esterification procedure based on anhydride chemistry using the following reagents (amounts): mPEG(5k)-G3-(Se)$_4$(OH)$_8$ **(31)** (200 mg, 0.03 mmol), pentenoic anhydride (86 mg, 0.47 mmol), DMAP (6 mg, 0.05 mmol), pyridine (1 ml). Polymer **34** was purified by precipitations in cold ether three times obtaining a white solid (217 mg, 96 %). **^{1}H NMR** (400 MHz, $CDCl_3$) δ ppm: 5.80 (8H, m, H31), 5.00 (16H, m, H32), 4.37-4.16 (46H, m, H7, H11, H15, H20, H23 and H27), 3.62 (452H, s, PEG), 3.37 (3H, s, H1), 2.78 (16H, m, H21 and H22), 2.62 (16H, m, H17 and H18), 2.47-2.28 (32H, m, H29 and H30), 1.25 (3H, s, H10), 1.24 (12H, s, H26), 1.22 (6H, s, H14). **^{13}C NMR** (101 MHz, $CDCl_3$) δ ppm: 172.3- 171.7 (C8, C12, C16, C19, C24 and C28), 136.5 (C31), 115.8 (C32), 70.7 (PEG), 68.9-64.0 (C7, C11, C15, C20, C23 and C27), 59.1 (C1), 49.4 (C9, C13 and C25), 33.4 (C29), 31.0 (C30), 28.8 (C17 and C18), 21.8 (C21 and C22), 17.9-17.6 (C10, C14 and C26). **^{77}Se NMR** (76 MHz, $CDCl_3$) δ ppm: 122.9 (-Se-). **SEC(DMF)** M_n = 6771 g/mol, M_w= 7608 g/mol, Đ = 1.1.

Figures

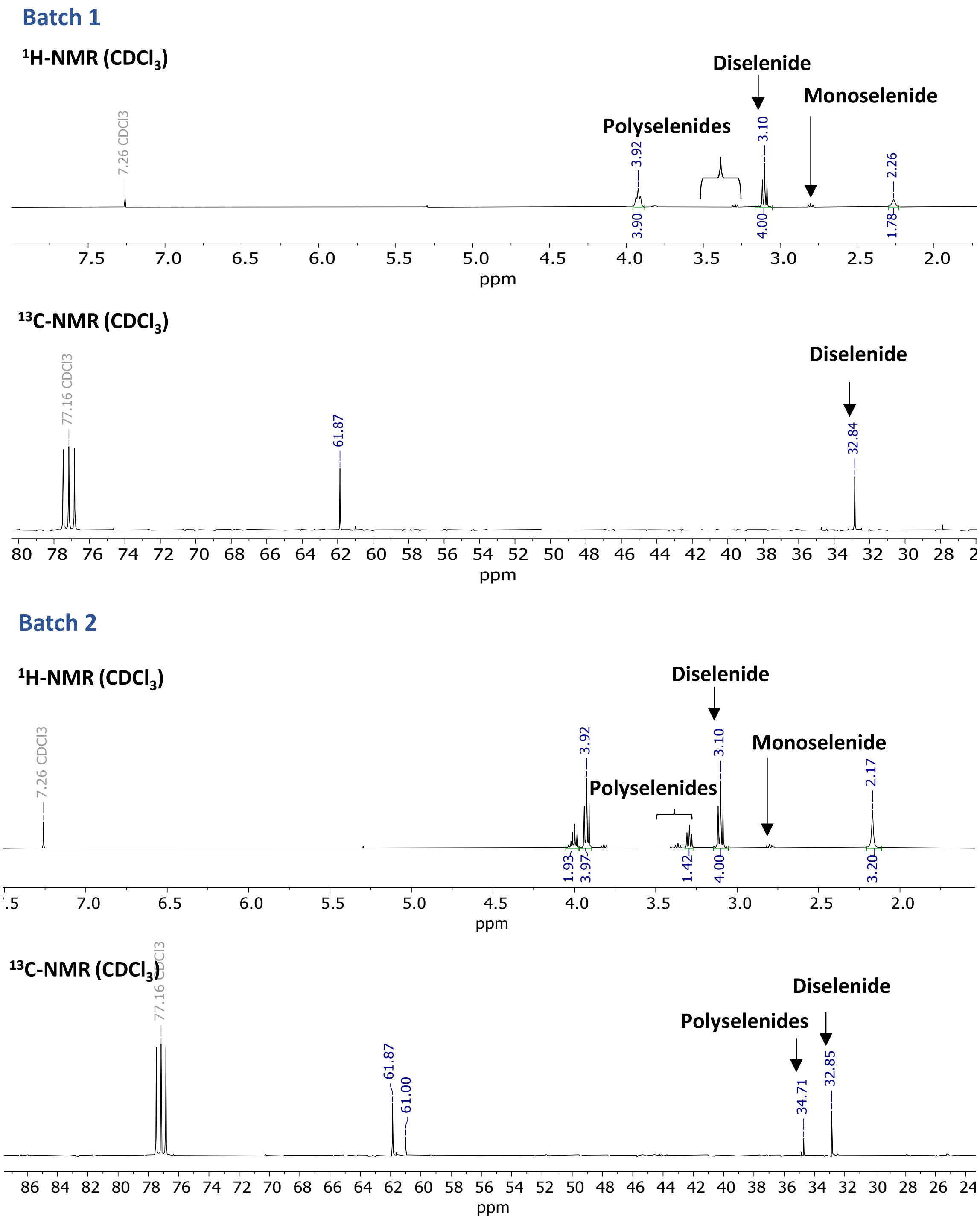

Figure S1. Complex mixture of polyselenides and monoselenides in the synthesis of 2-hydroxyethyl diselenide (**1**) of different batches in $CDCl_3$.

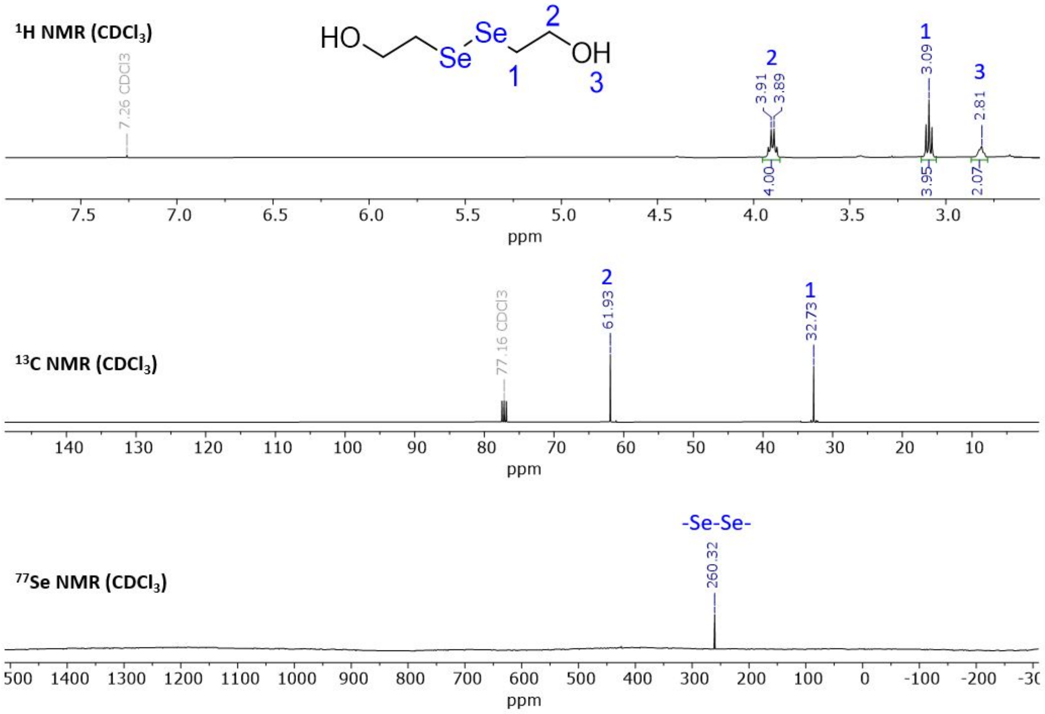

Figure S2. ^{1}H, ^{13}C and ^{77}Se NMR of 2-hydroxyethyl diselenide (**1**) in $CDCl_3$.

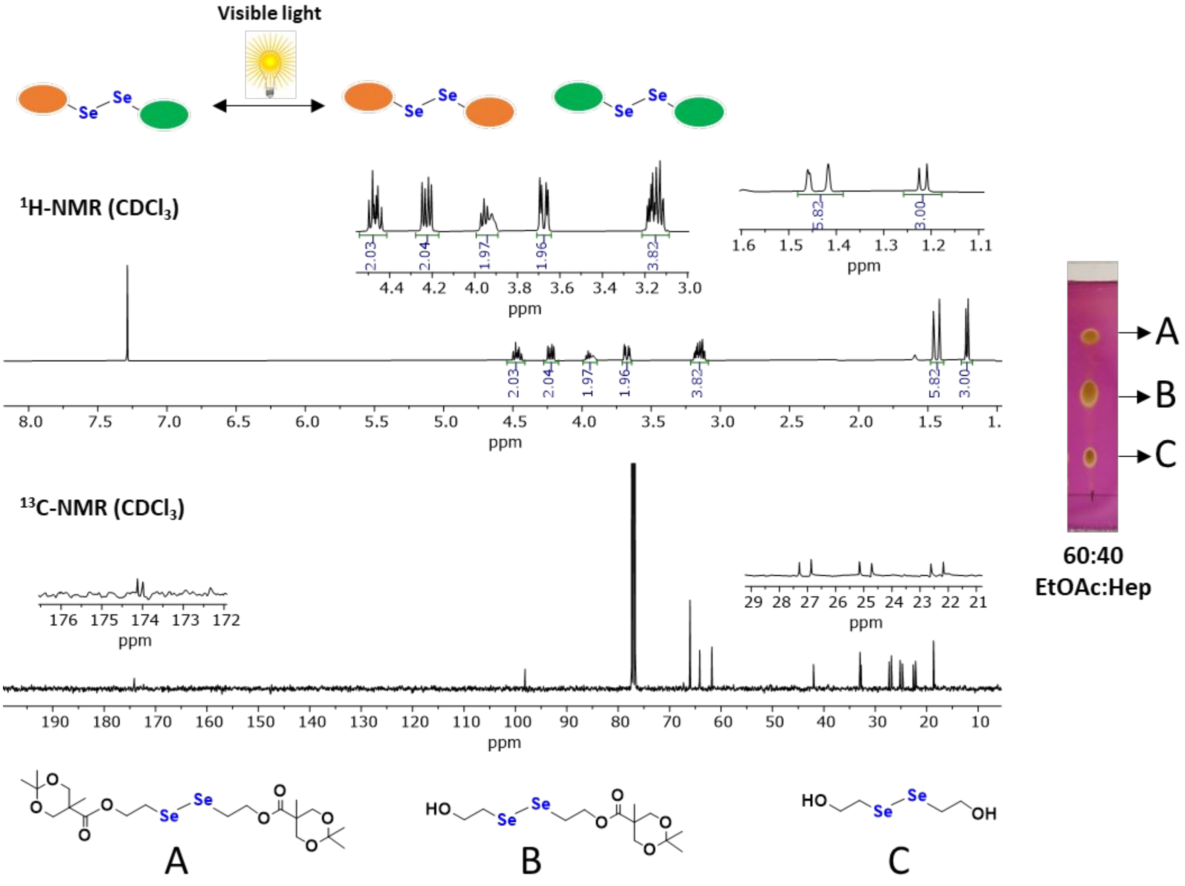

Figure S3. ^{1}H and ^{13}C NMR in $CDCl_3$ of the asymmetric $AB_2^{Se\text{-}Se}$ contaminated with both symmetric derivates after silica chromatography purification.

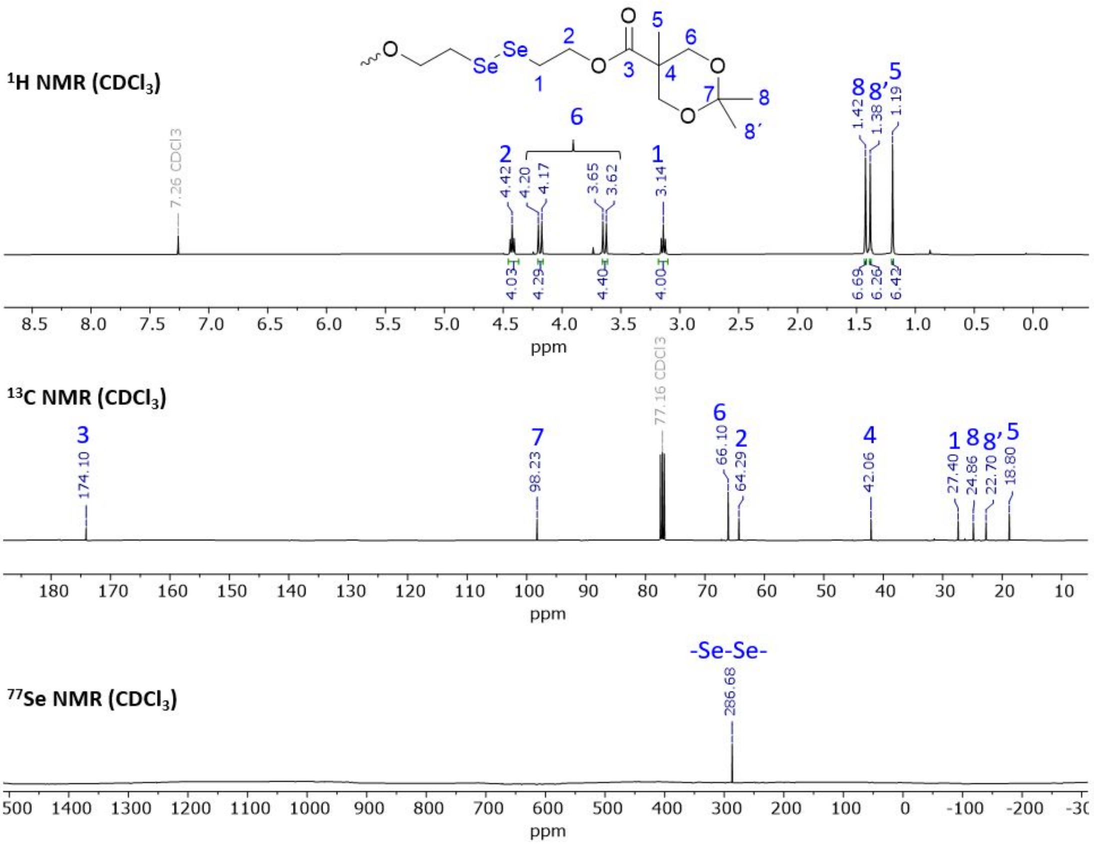

Figure S4. ^{1}H,^{13}C and ^{77}Se NMR of Se_2-G1-$(Ac)_2$ **(2)** in $CDCl_3$.

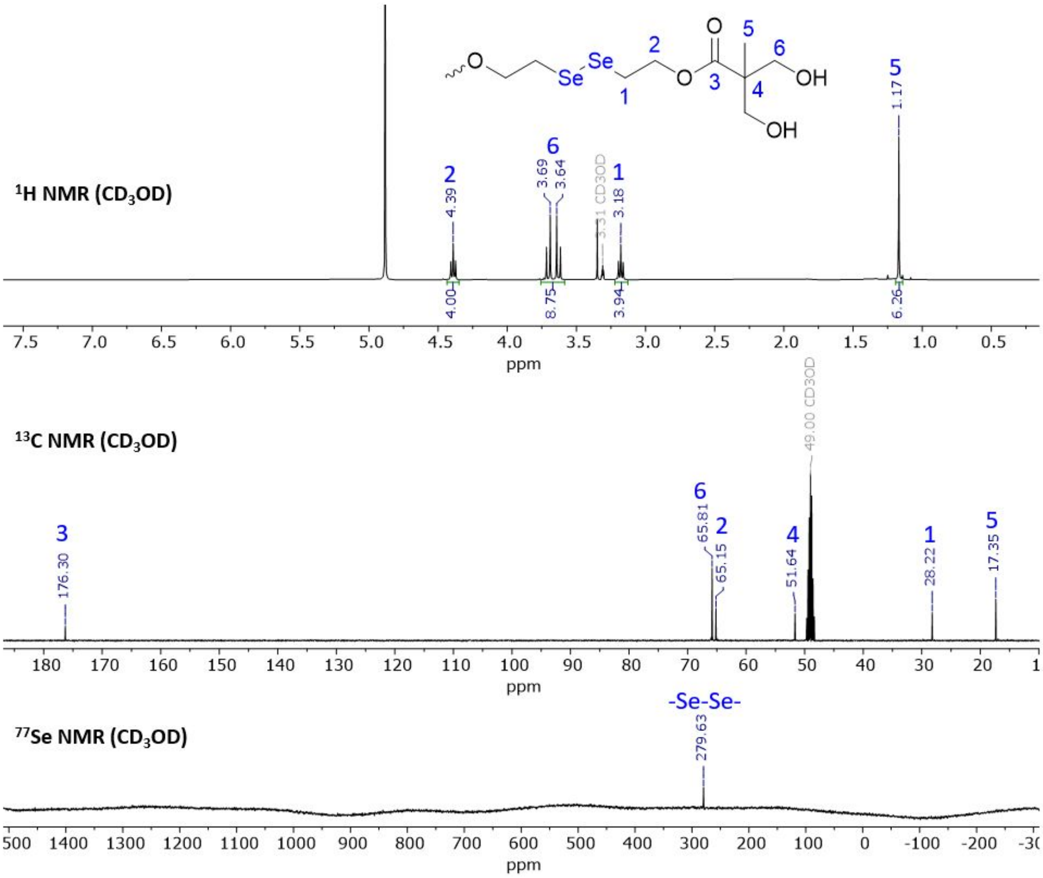

Figure S5. ^{1}H, ^{13}C and ^{77}Se NMR of Se_2-G1-$(OH)_4$ **(3)** in CD_3OD.

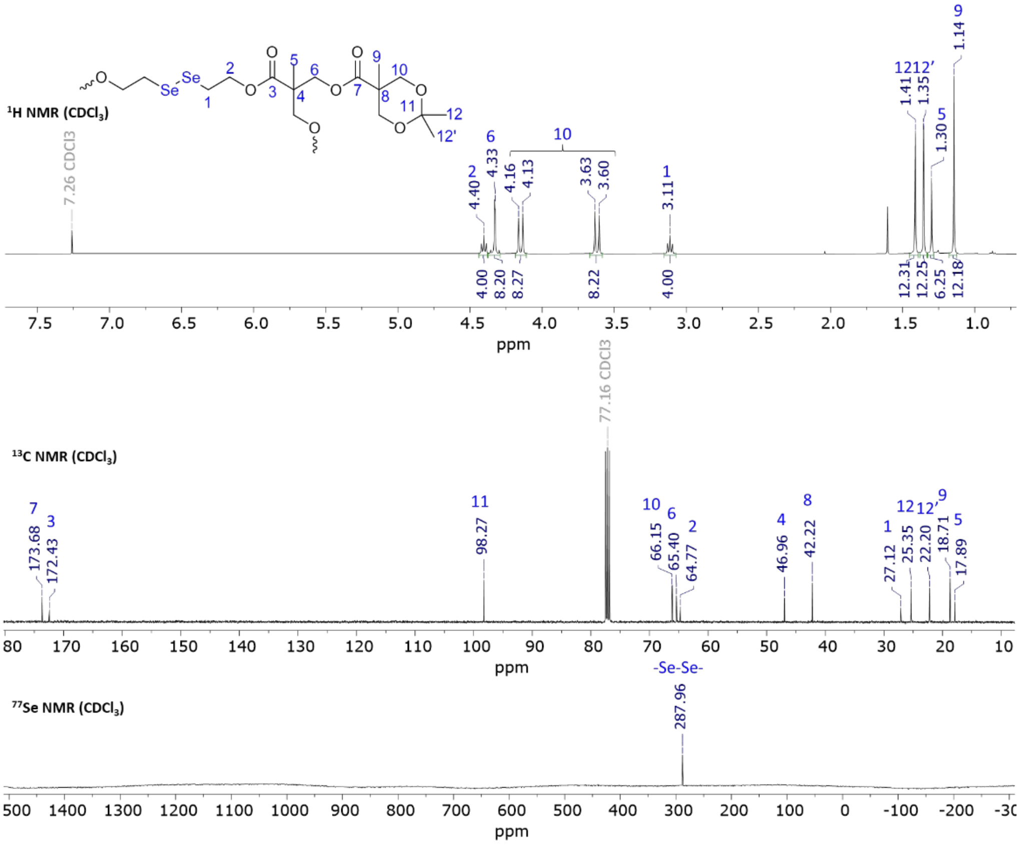

Figure S6. ^{1}H, ^{13}C and ^{77}Se NMR of Se_2-G2-$(Ac)_4$ **(4)** in $CDCl_3$.

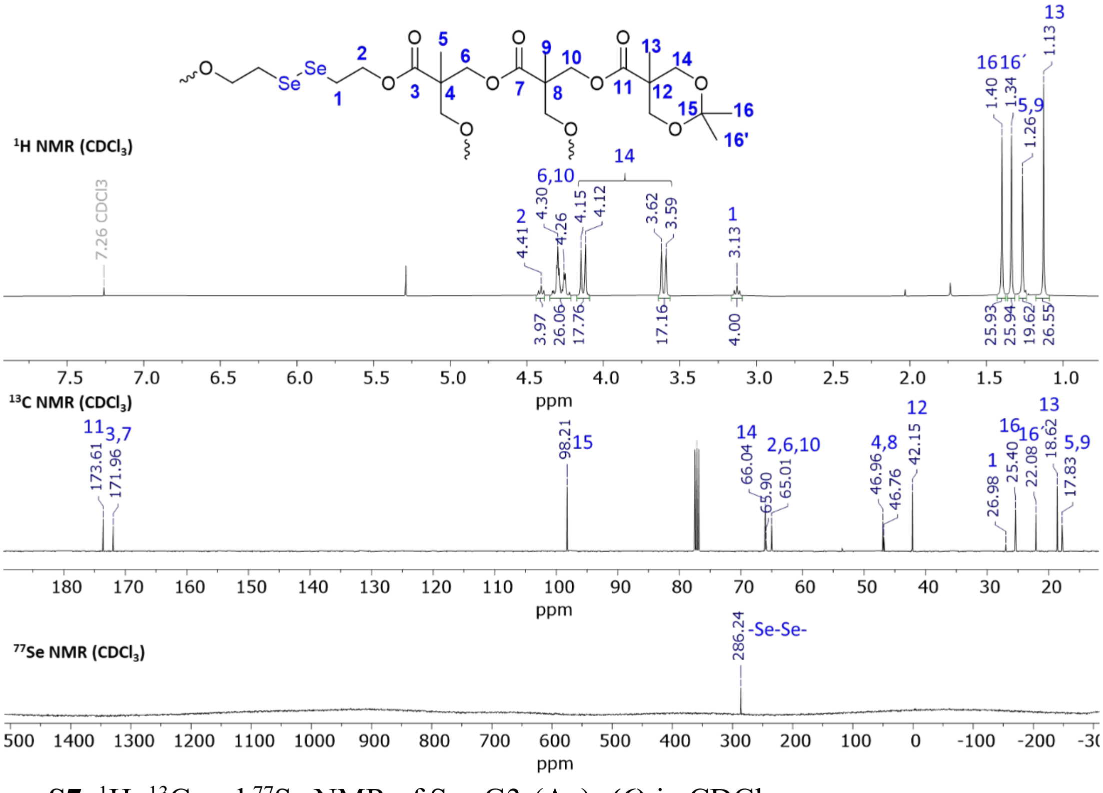

Figure S7. ^{1}H, ^{13}C and ^{77}Se NMR of Se_2-G3-$(Ac)_8$ **(6)** in $CDCl_3$.

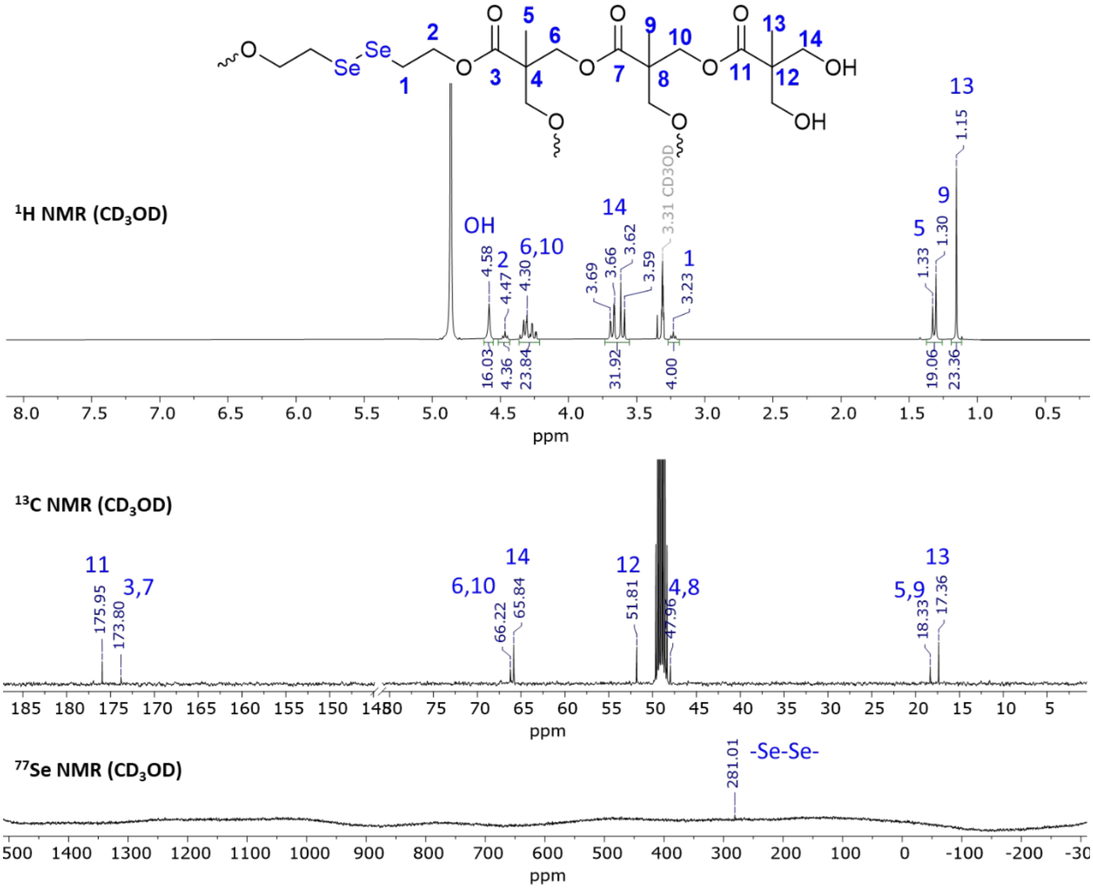

Figure S8. ^{1}H, ^{13}C and ^{77}Se NMR of Se_2-G3-$(OH)_{16}$ **(7)** in CD_3OD.

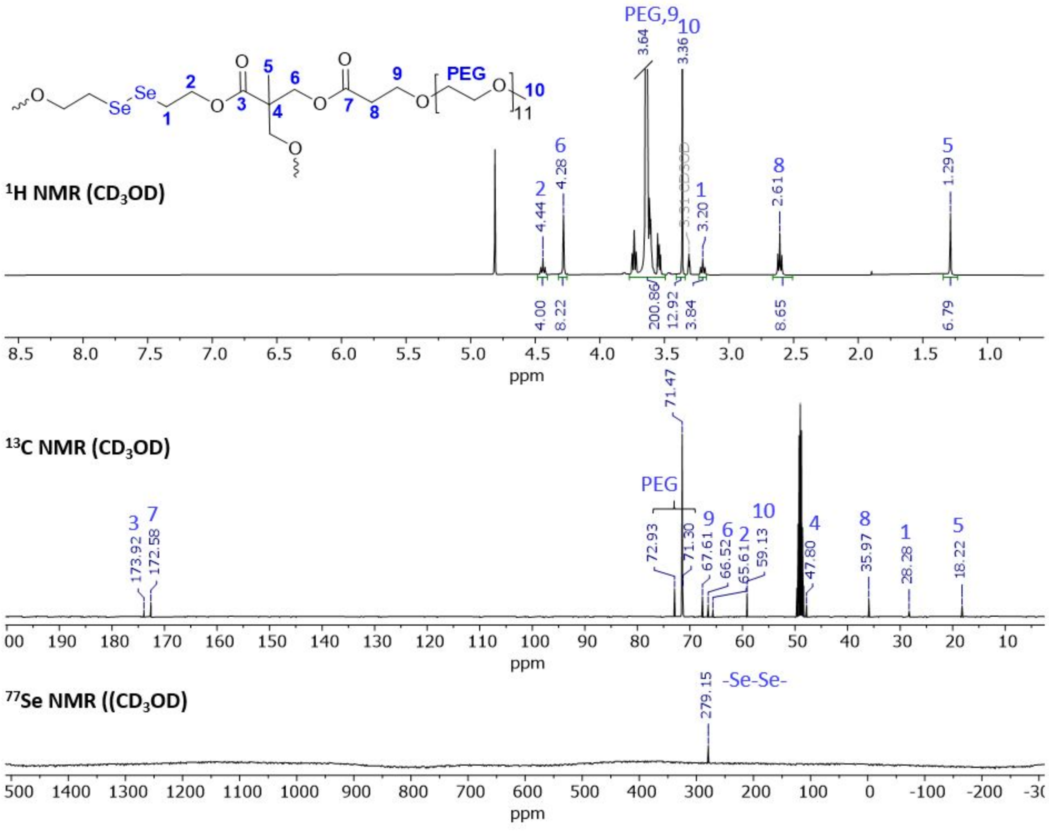

Figure S9. ^{1}H, ^{13}C and ^{77}Se NMR of Se_2-G1-$(mPEG)_4$ **(8)** in CD_3OD.

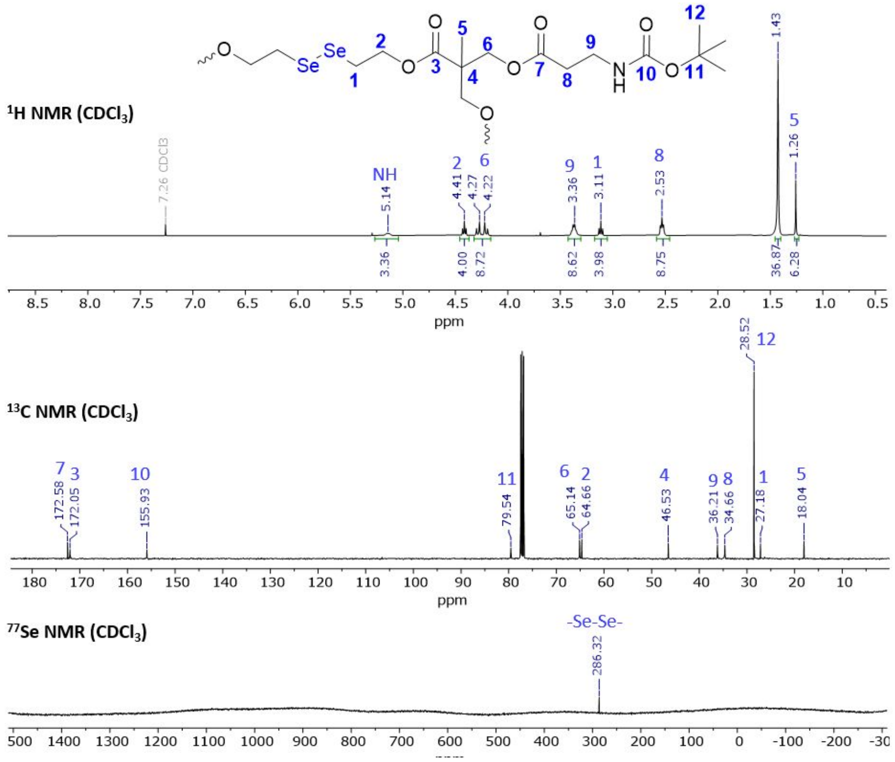

Figure S10. ^{1}H, ^{13}C and ^{77}Se NMR of Se_2-G1-$(NHBoc)_4$ **(9)** in $CDCl_3$.

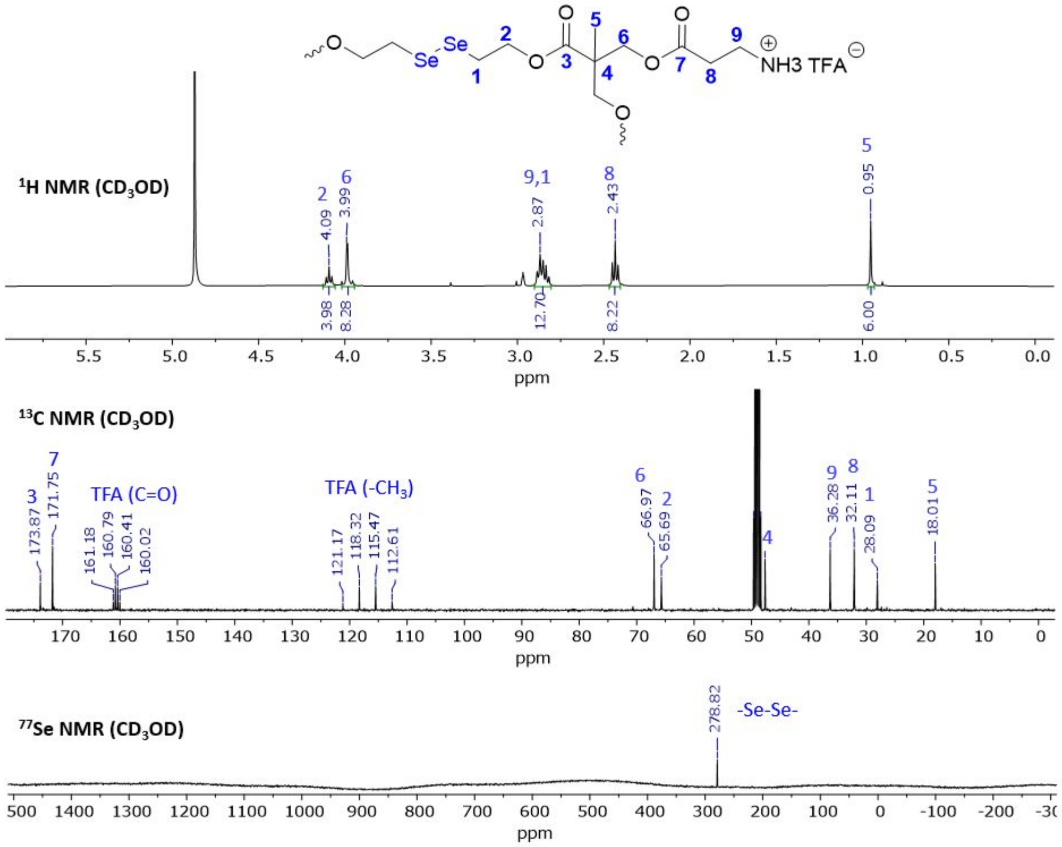

Figure S11. ^{1}H, ^{13}C and ^{77}Se NMR of Se_2-G1-$(NH_3^+)_4$ **(10)** in CD_3OD.

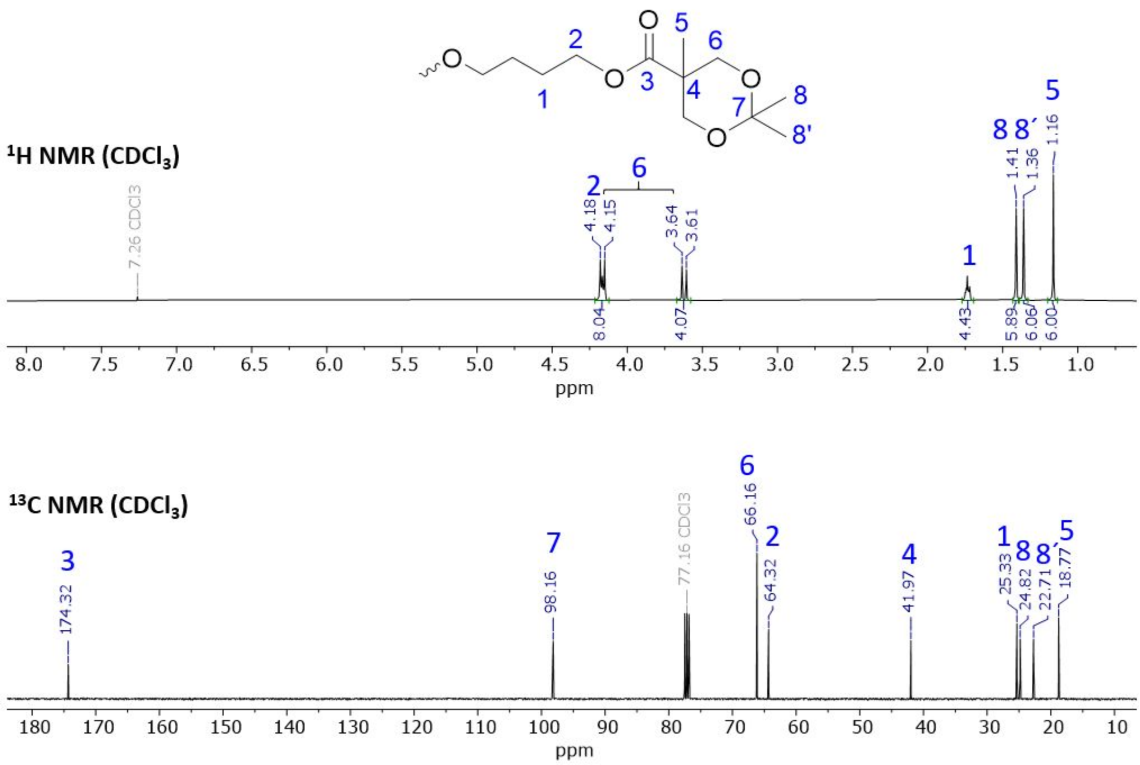

Figure S12. ^{1}H and ^{13}C NMR of But-G1-$(Ac)_2$ **(11)** in $CDCl_3$.

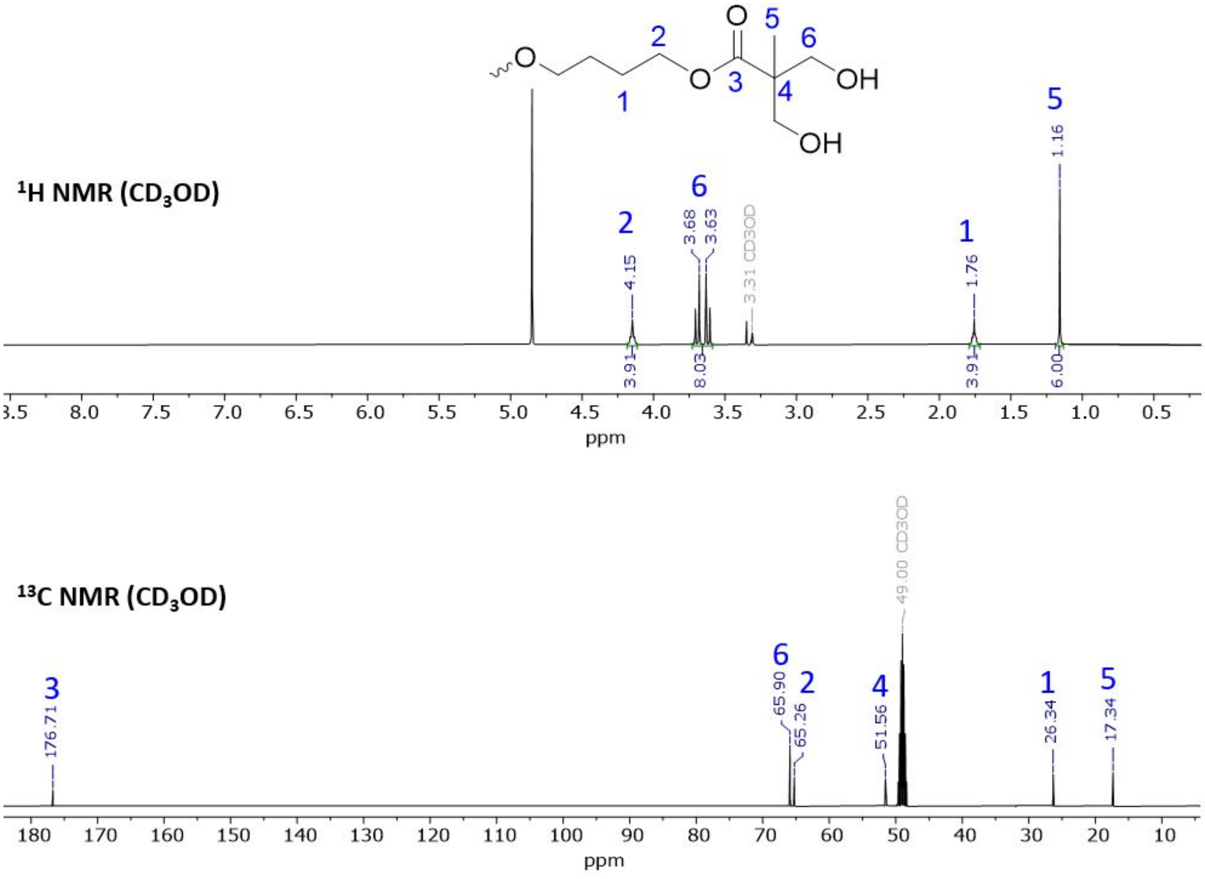

Figure S13. ^{1}H and ^{13}C NMR of But-G1-$(OH)_4$ **(12)** in CD_3OD.

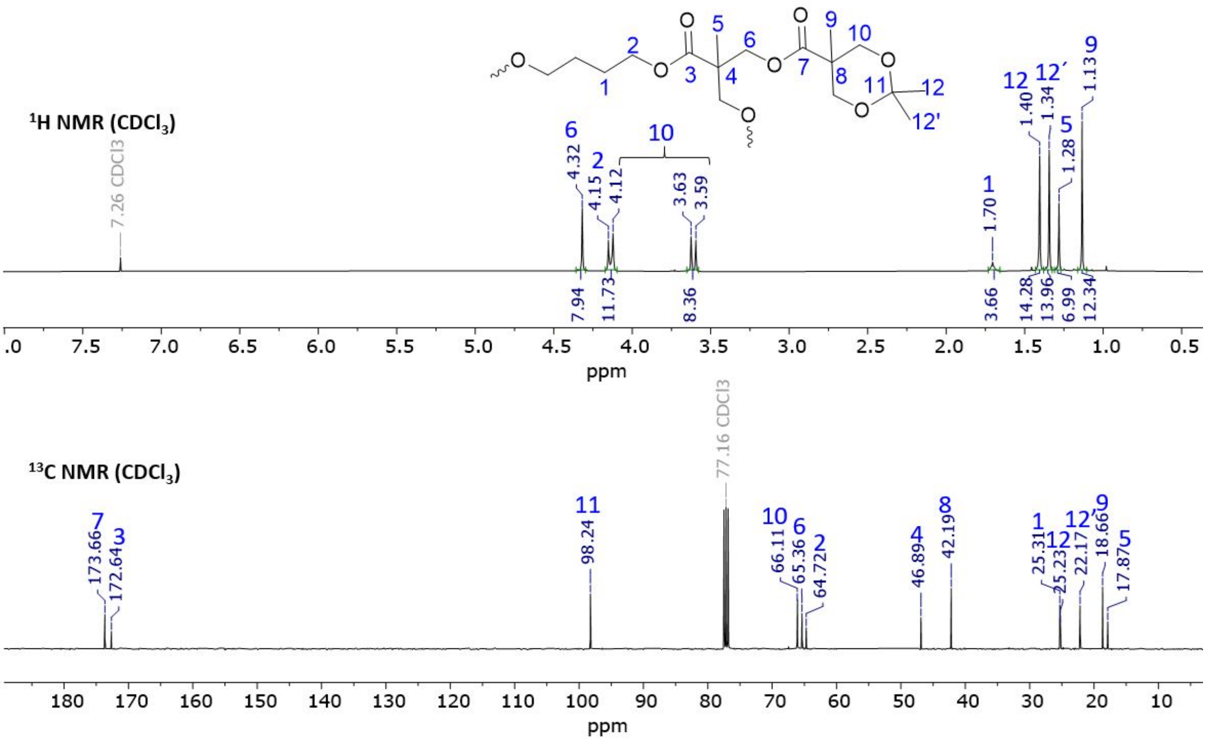

Figure S14. ^{1}H and ^{13}C NMR of But-G2-$(Ac)_4$ **(13)** in $CDCl_3$.

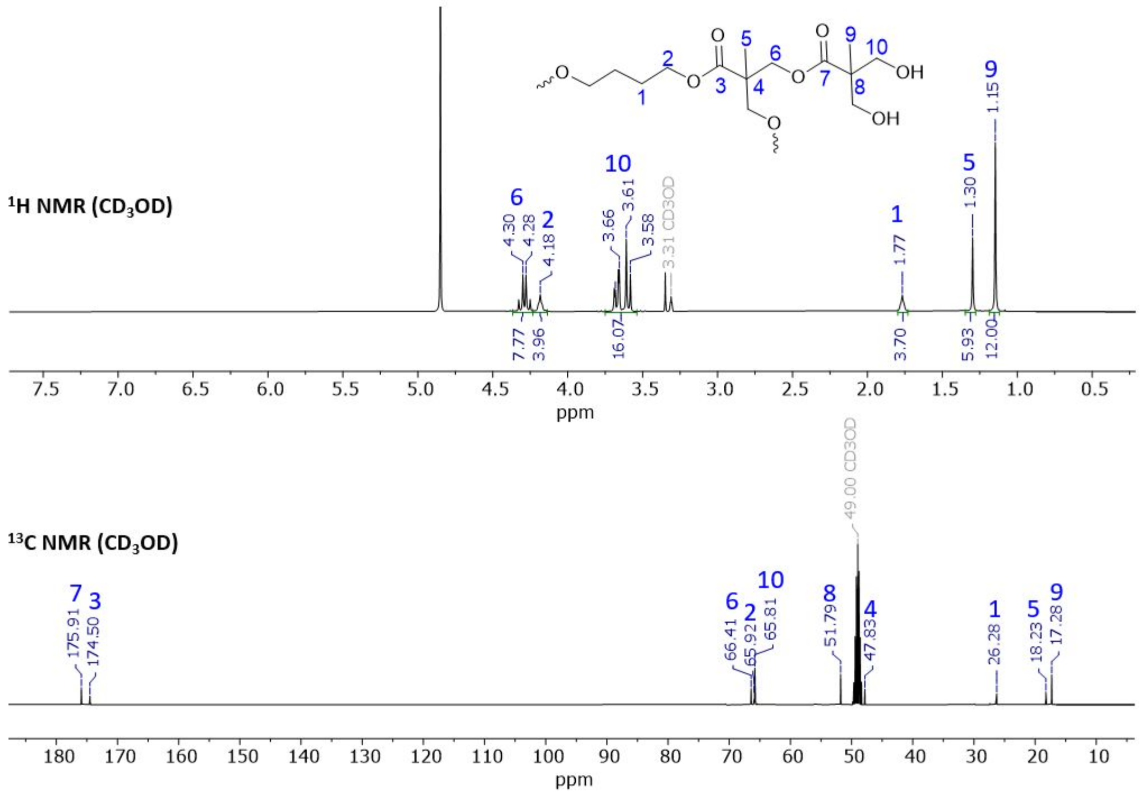

Figure S15. ^{1}H and ^{13}C NMR of But-G2-$(OH)_8$ **(14)** in CD_3OD.

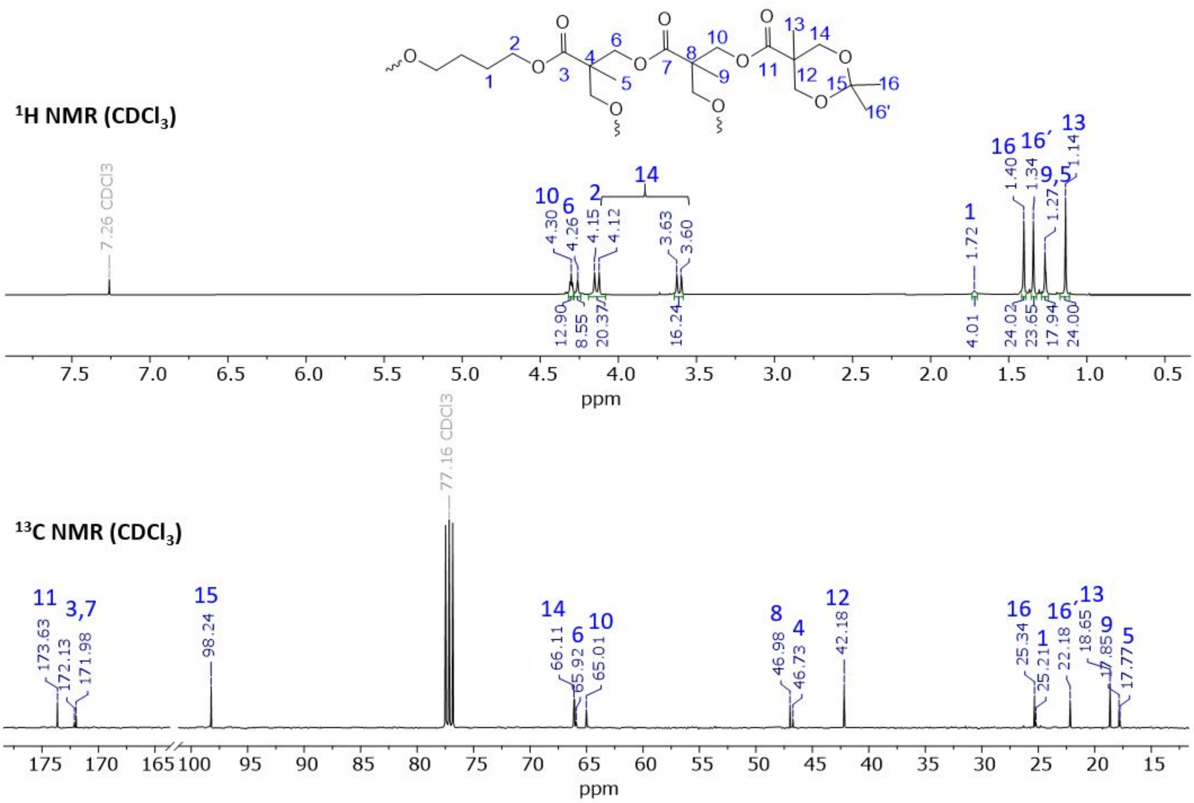

Figure S16. ^{1}H and ^{13}C NMR of But-G3-$(Ac)_8$ **(15)** in $CDCl_3$.

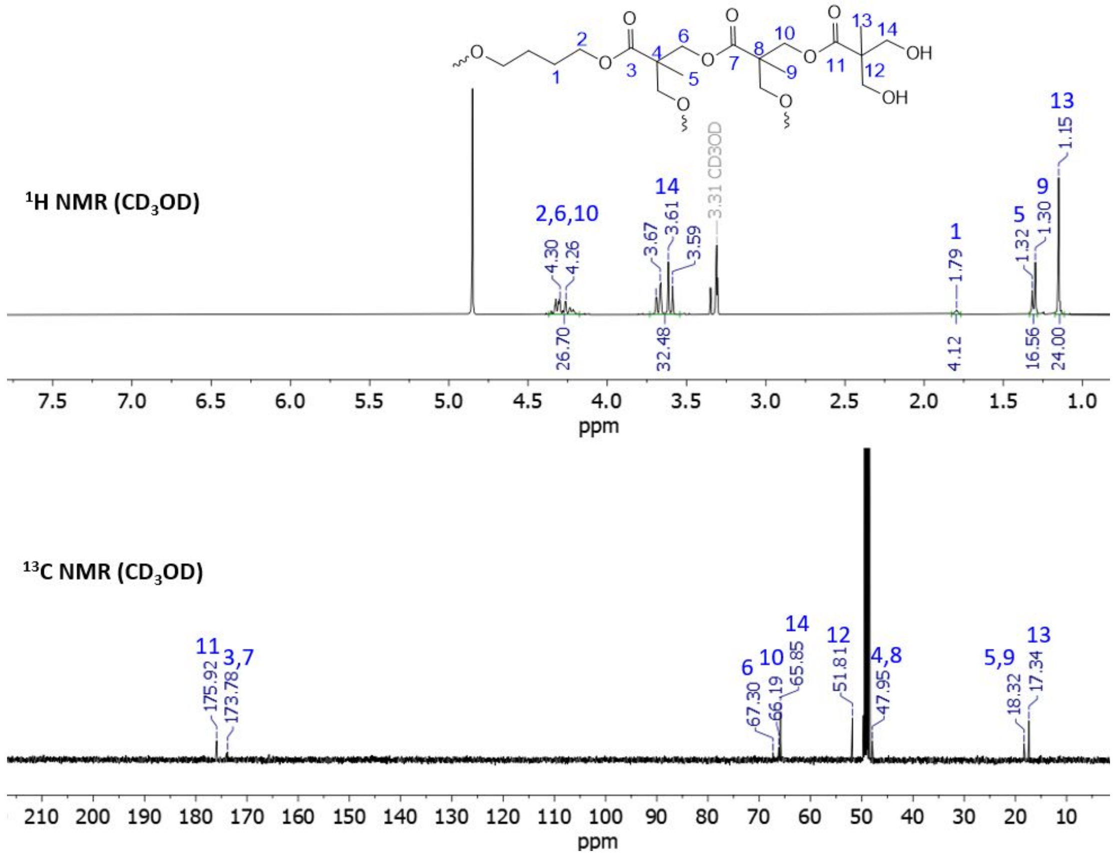

Figure S17. ^{1}H and ^{13}C NMR of But-G3-$(OH)_{16}$ **(16)** in CD_3OD.

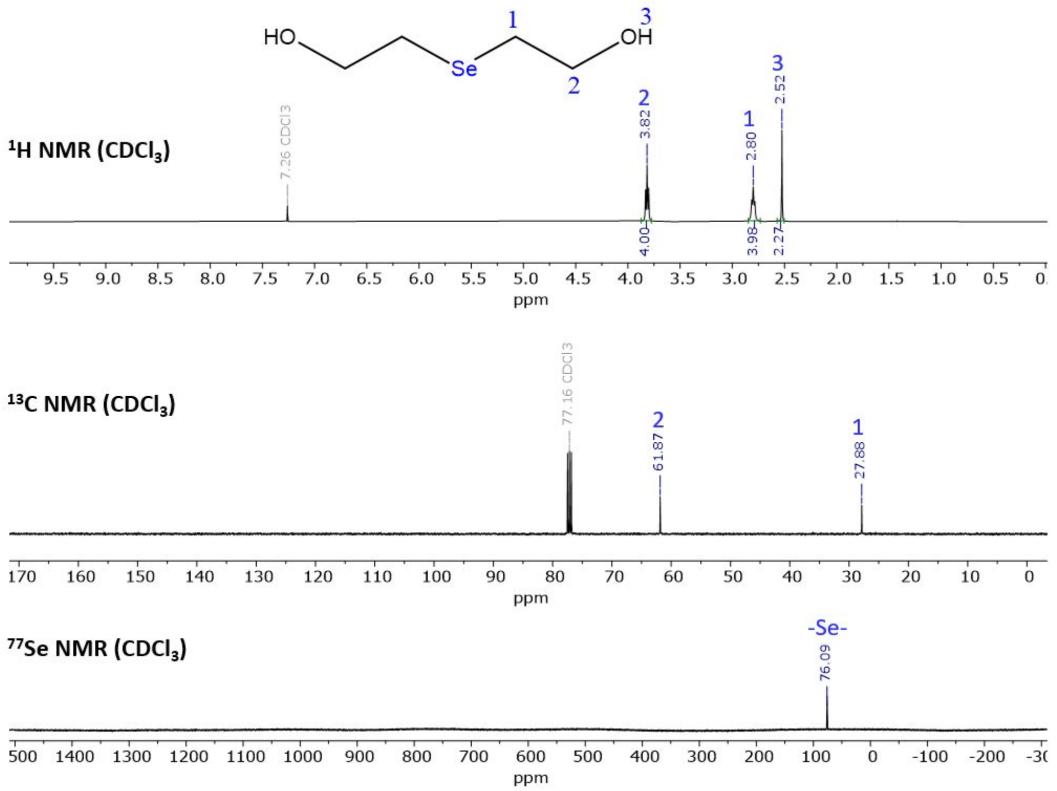

Figure S18. ^{1}H, ^{13}C and ^{77}Se NMR of 2-hydroxyethyl selenide **(17)** in $CDCl_3$.

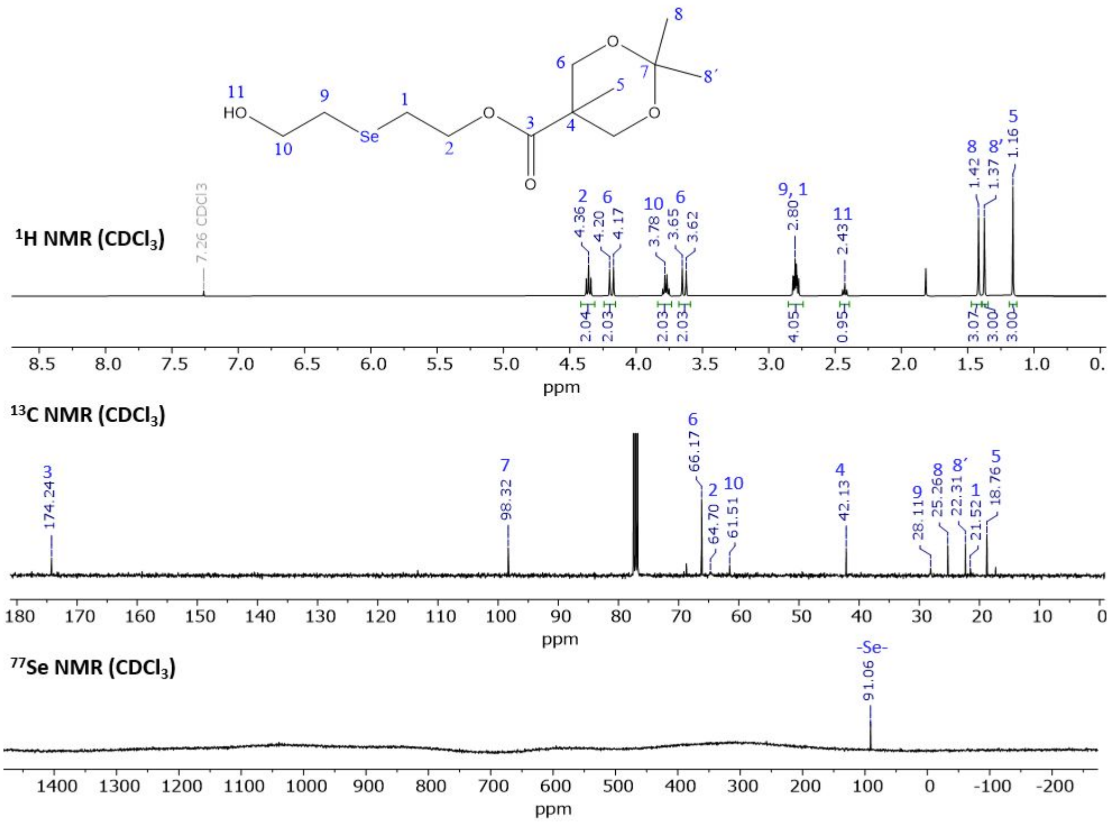

Figure S19. ^{1}H, ^{13}C and ^{77}Se NMR of 2-((2-hydroxyethyl)selanyl)ethyl 2,2,5-trimethyl-1,3-dioxane-5-carboxylate **(18)** in $CDCl_3$.

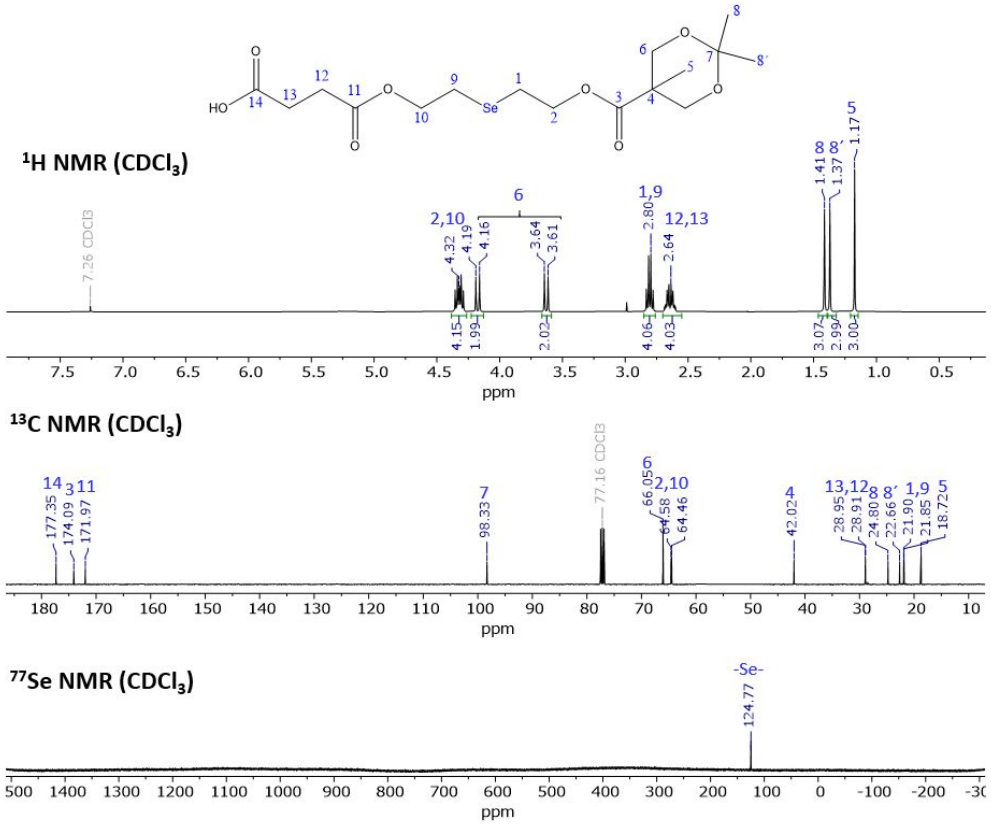

Figure S20. ^{1}H, ^{13}C and ^{77}Se NMR of 4-oxo-4-(2-((2-((2,2,5-trimethyl-1,3-dioxane-5carbonyl) oxy)ethyl)selanyl) ethoxy) butanoic acid **(19)** in $CDCl_3$.

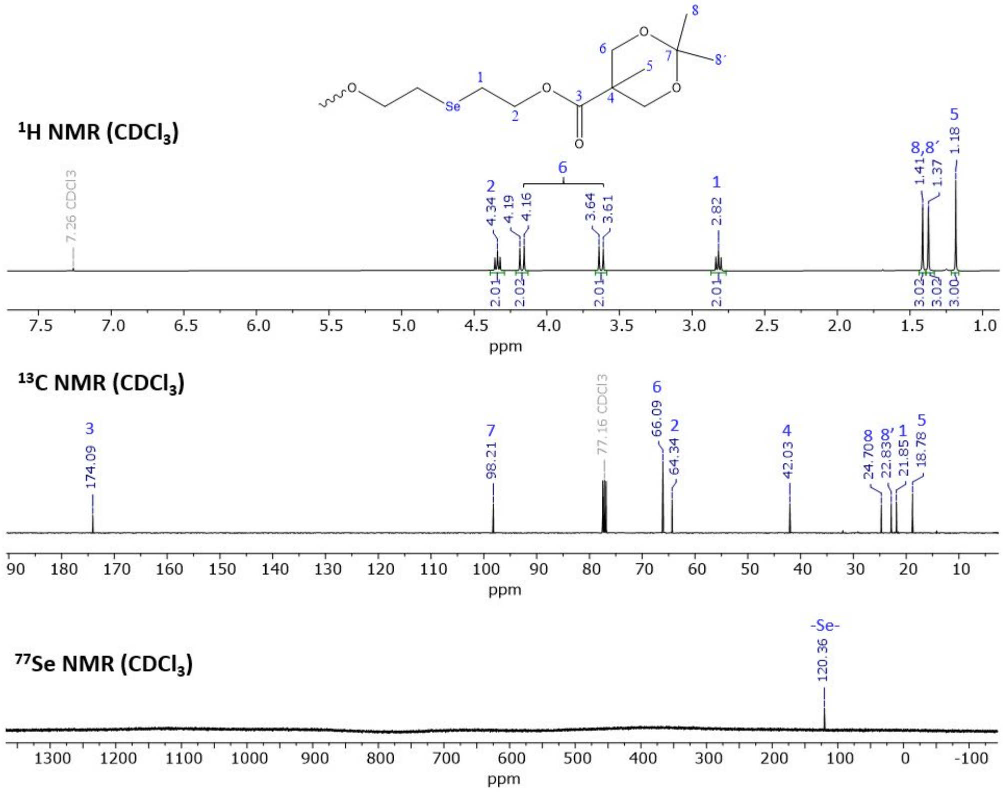

Figure S21. ^{1}H, ^{13}C and ^{77}Se NMR of Se-G1-$(Ac)_2$ **(20)** in $CDCl_3$.

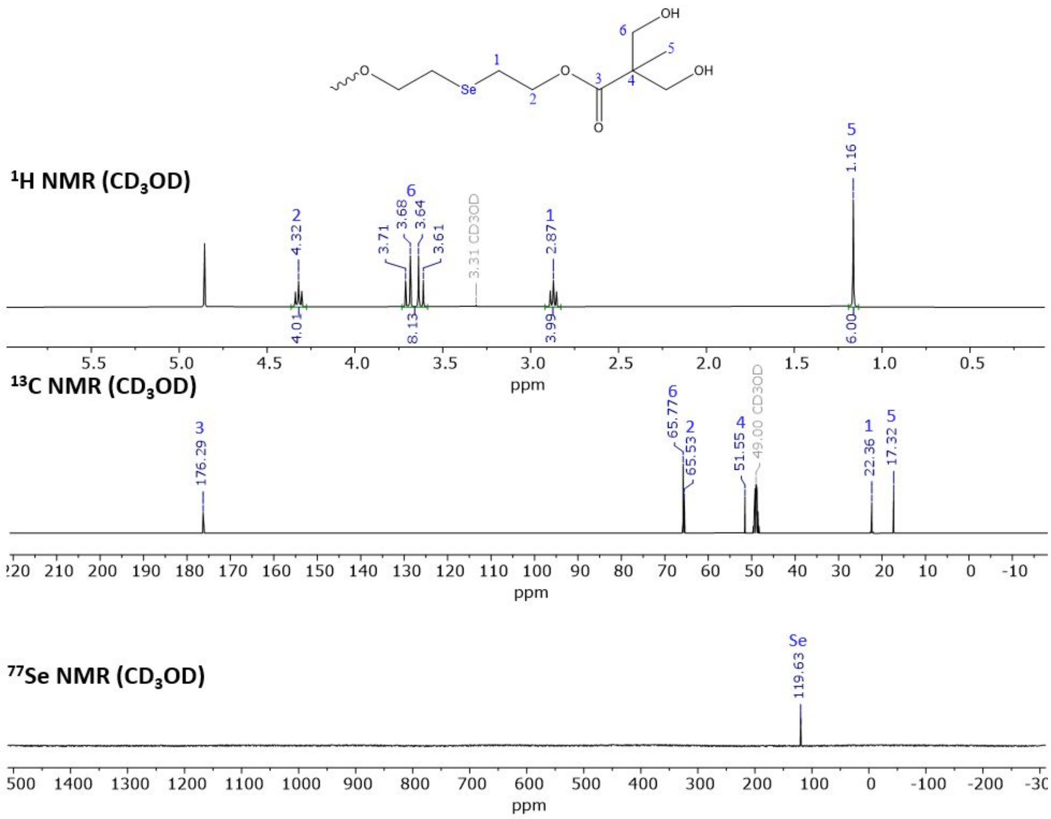

Figure S22. ^{1}H, ^{13}C and ^{77}Se NMR of Se-G1-$(OH)_4$ **(21)** in CD_3OD.

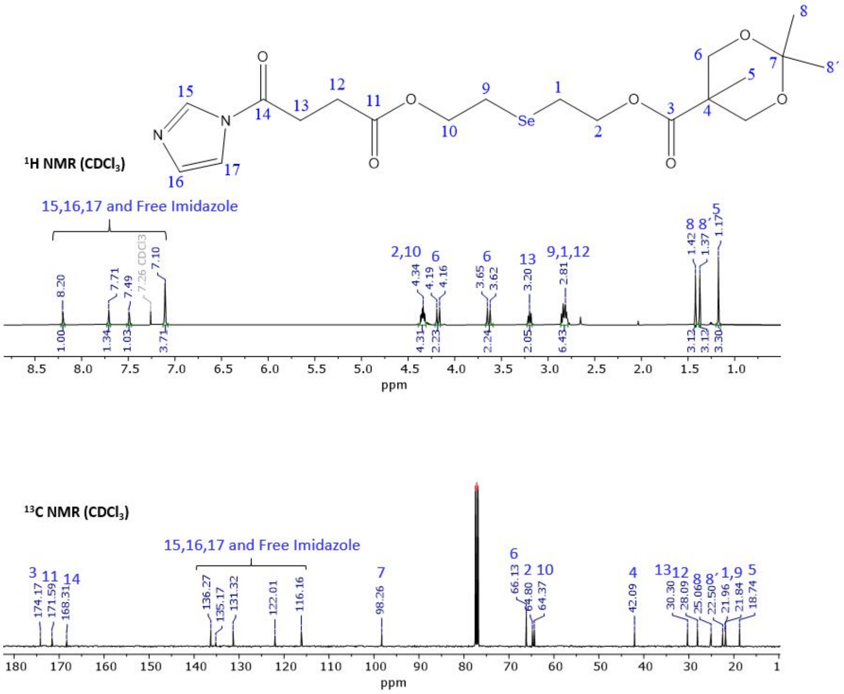

Figure S23. ^{1}H and ^{13}C NMR of imidazolide hydroxyethyl activated 4-oxo-4-(2-((2-((2,2,5-trimethyl-1,3-dioxane-5carbonyl) oxy)ethyl)selanyl) ethoxy) butanoic acid in $CDCl_3$.

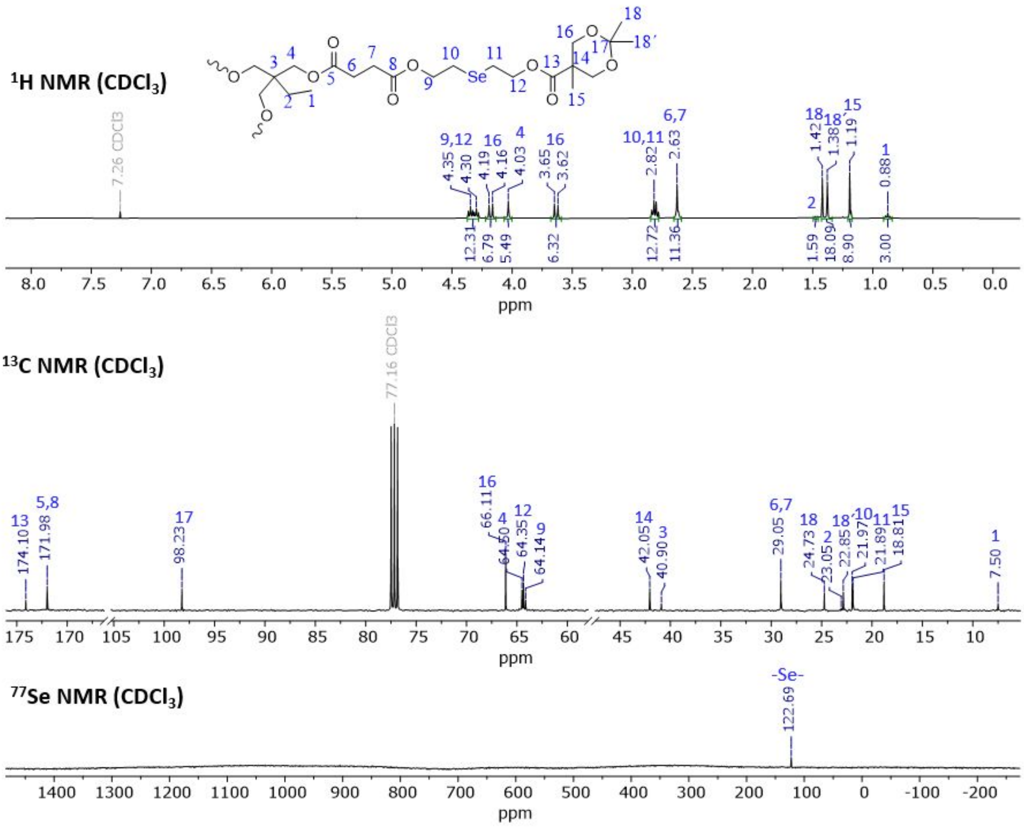

Figure S24. ^{1}H, ^{13}C and ^{77}Se NMR of TMP-G1-(Se)$_3$(Ac)$_3$ **(22)** in $CDCl_3$.

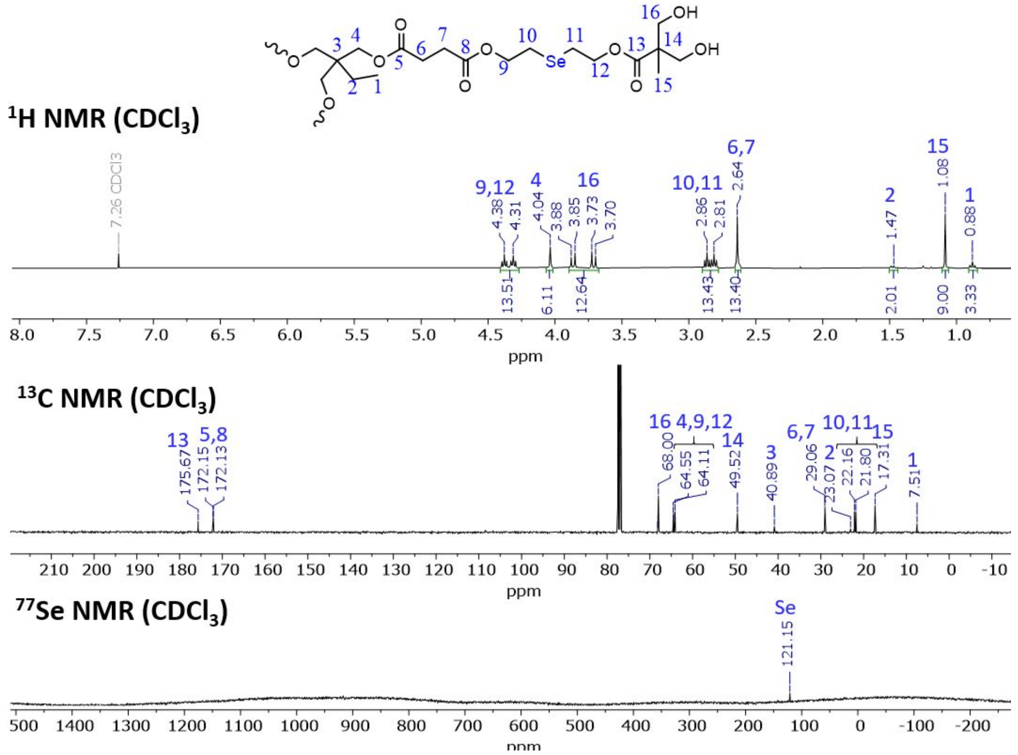

Figure S25. ^{1}H, ^{13}C and ^{77}Se NMR of TMP-G1-(Se)$_3$(OH)$_6$ **(23)** in $CDCl_3$.

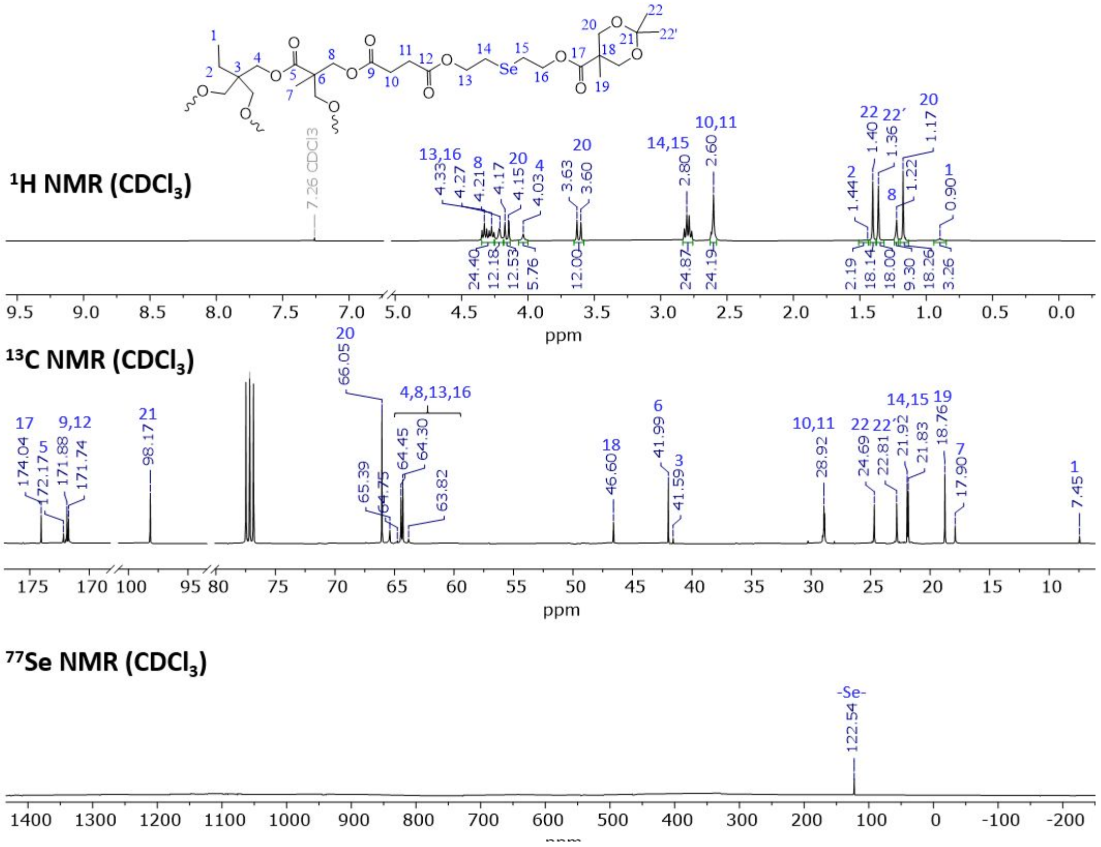

Figure S26. ^{1}H, ^{13}C and ^{77}Se NMR of TMP-G2-(Se)$_9$(Ac)$_6$ **(24)** in $CDCl_3$.

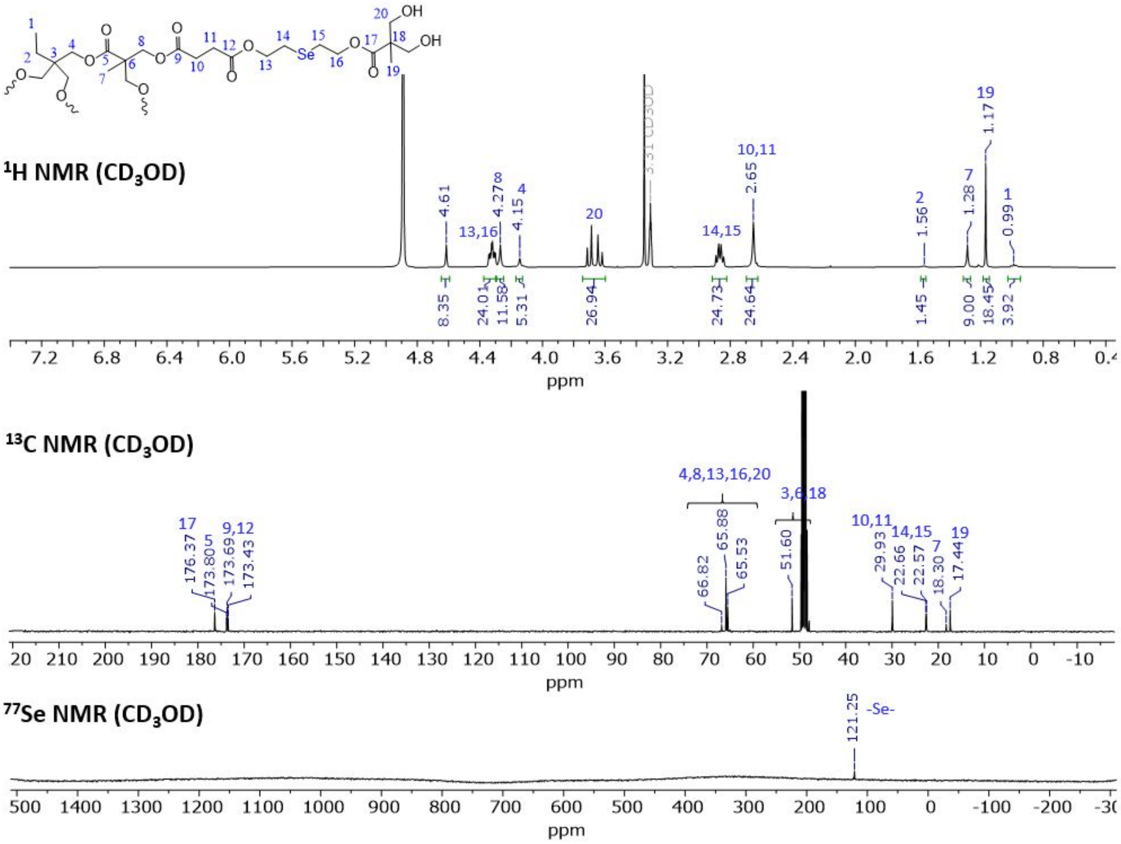

Figure S27.^{1}H, ^{13}C and ^{77}Se NMR of TMP-G2-(Se)$_9$(OH)$_{12}$ **(25)** in CD_3OD.

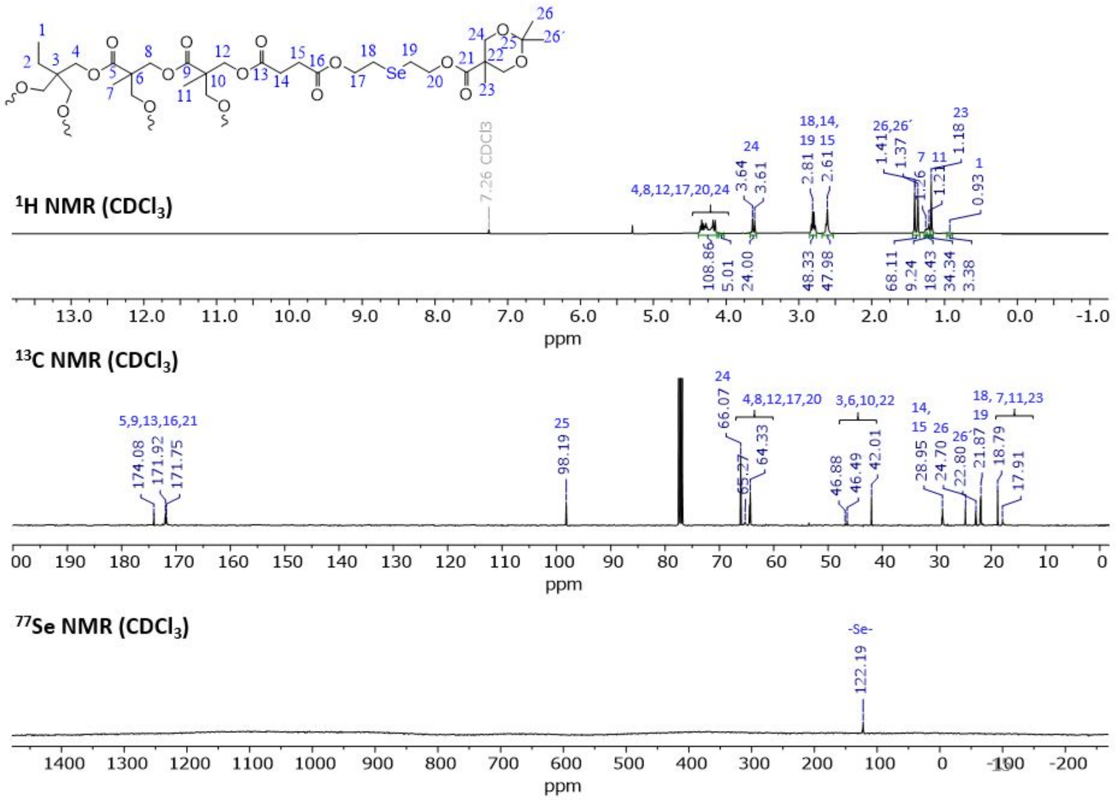

Figure S28. ^{1}H, ^{13}C and ^{77}Se NMR of TMP-G3-(Se)$_{12}$(Ac)$_{12}$ **(26)** in $CDCl_3$.

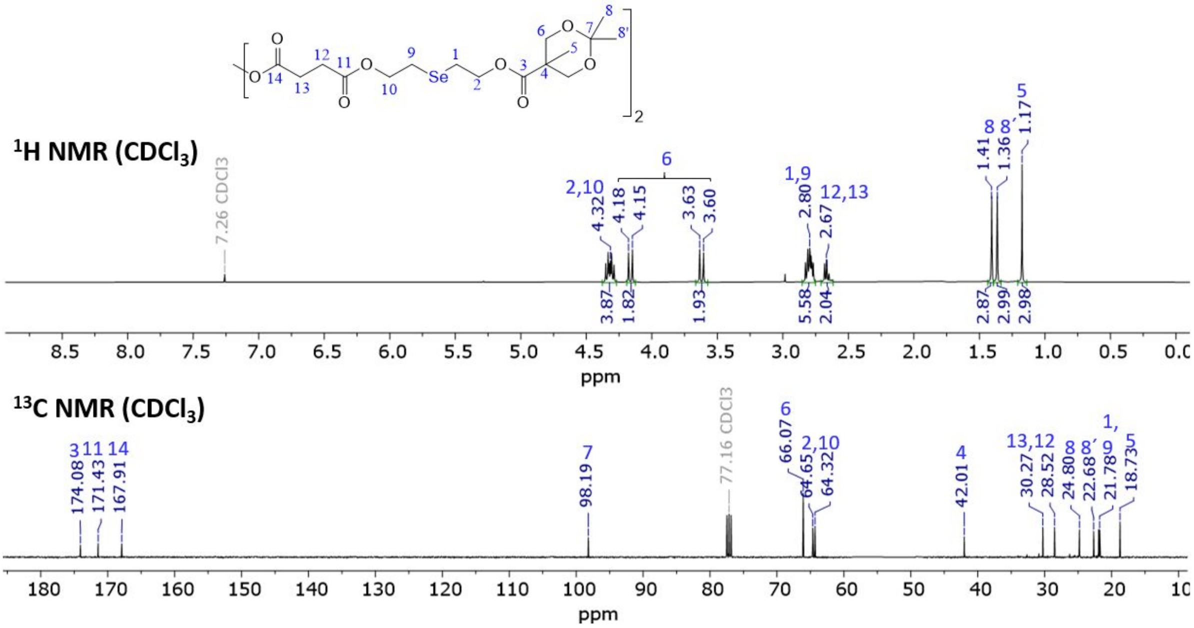

Figure S29. ^{1}H and ^{13}C NMR of the anhydride of the 4-oxo-4-(2-((2-((2,2,5-trimethyl-1,3-dioxane-5carbonyl) oxy)ethyl)selanyl) ethoxy) butanoic acid **(27)** in $CDCl_3$.

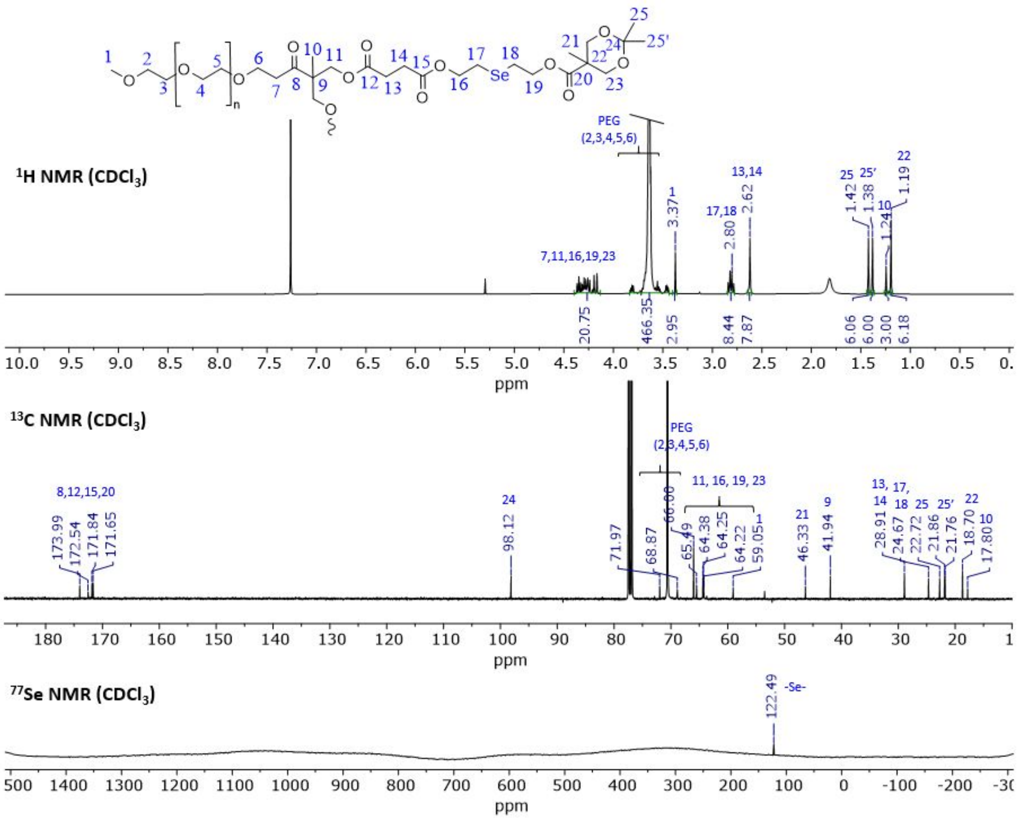

Figure S30. ^{1}H, ^{13}C and ^{77}Se NMR of mPEG(5k)-G2-$(Se)_2(Ac)_2$ **(28)** in $CDCl_3$.

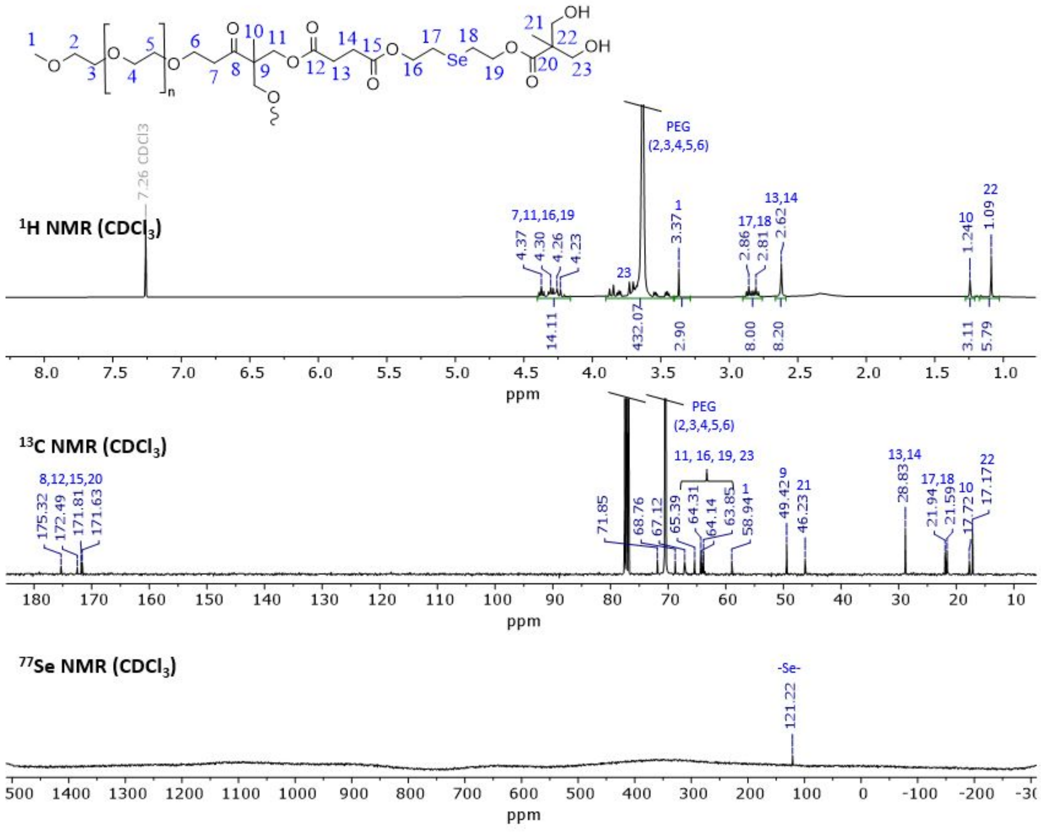

Figure S31. ^{1}H, ^{13}C and ^{77}Se NMR of mPEG(5k)-G2-$(Se)_2(OH)_4$ **(29)** in $CDCl_3$.

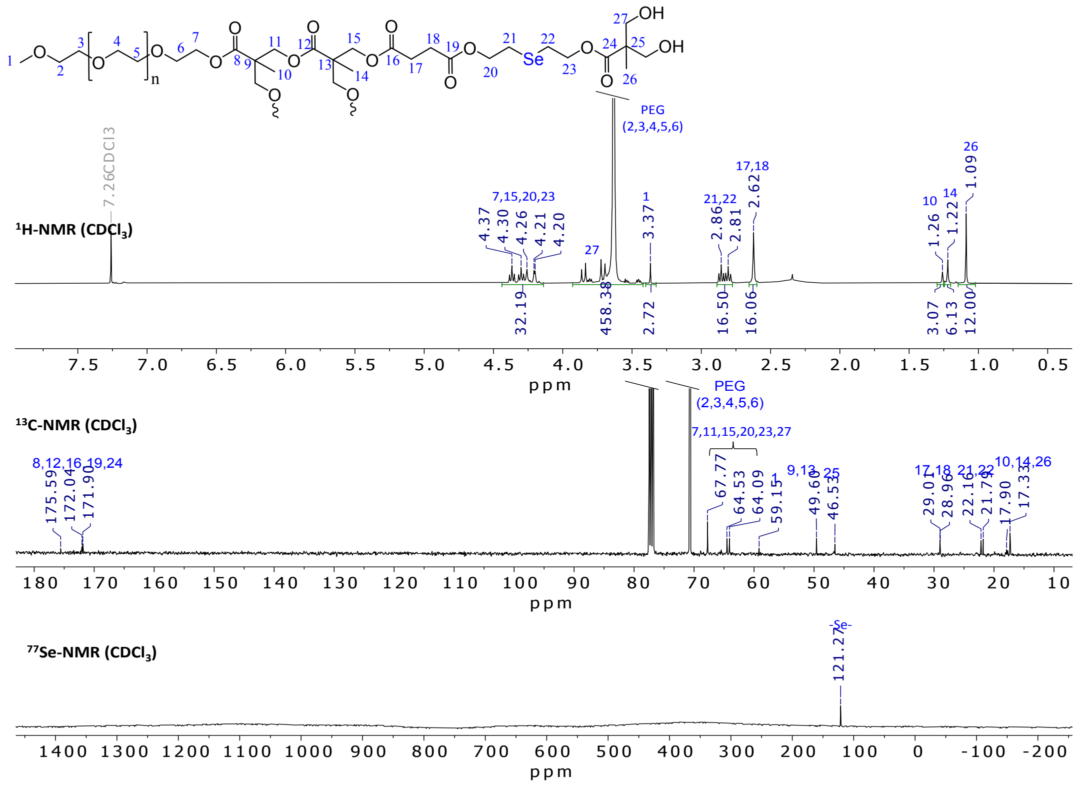

Figure S32. ^{1}H, ^{13}C and ^{77}Se NMR of mPEG(5k)-G3-$(Se)_4(OH)_8$ **(31)** in $CDCl_3$.

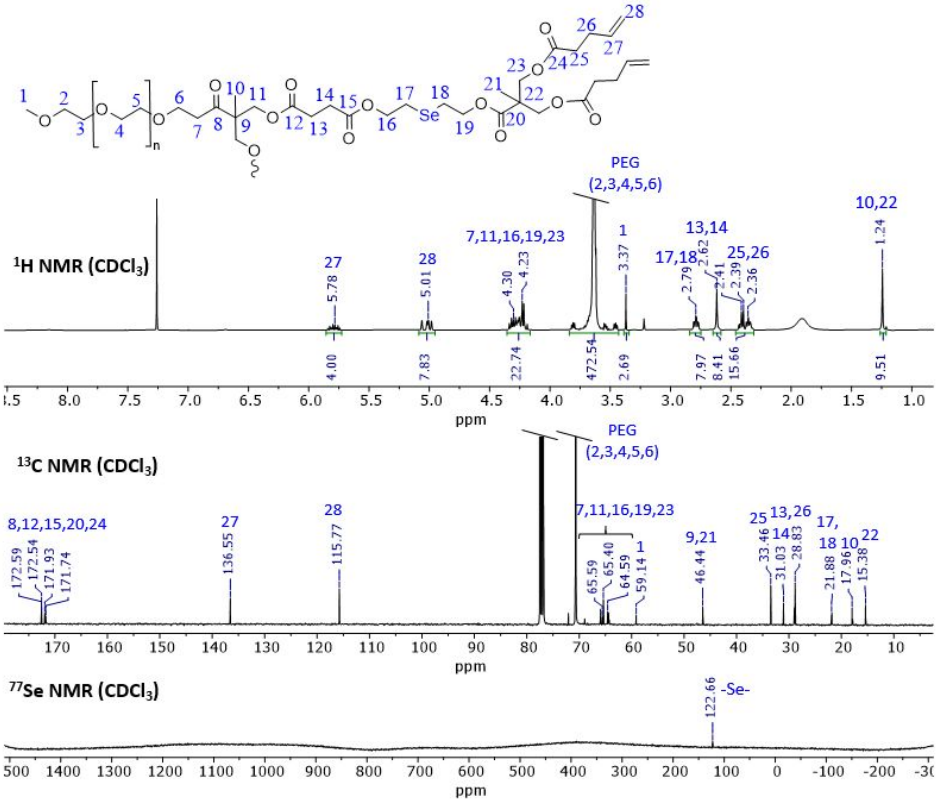

Figure S33. ^{1}H, ^{13}C and ^{77}Se NMR of mPEG(5k)-G2-$(Se)_2(Allyl)_4$ **(33)** in $CDCl_3$.

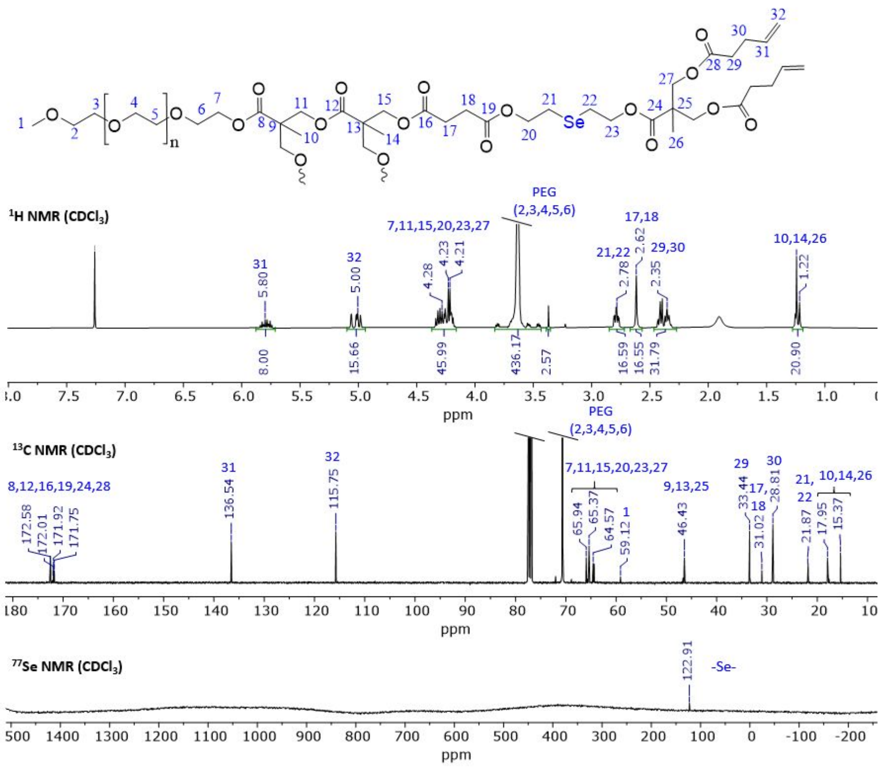

Figure S34. ^{1}H, ^{13}C and ^{77}Se NMR of mPEG(5k)-G3-(Se)$_4$(Allyl)$_8$ **(34)** in $CDCl_3$

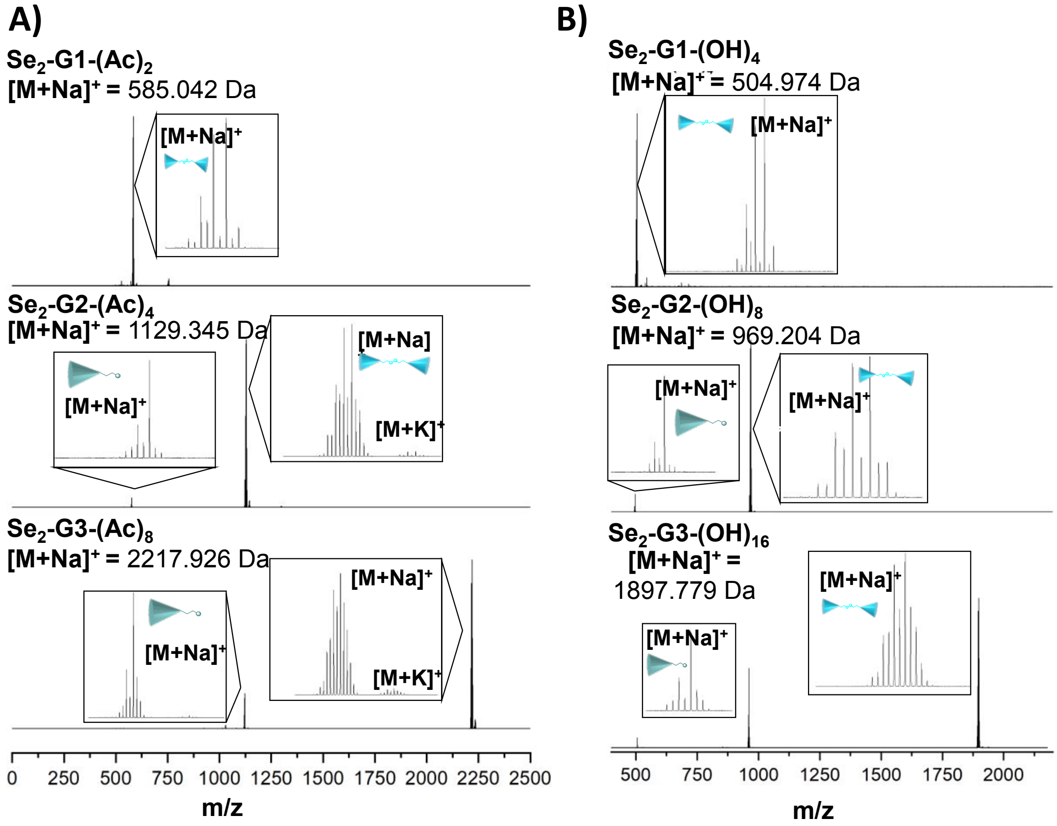

Figure S35. MALDI-ToF of the dendrimers A) Se_2-Gn-$(Ac)_m$ B) Se_2-Gn-$(OH)_m$.

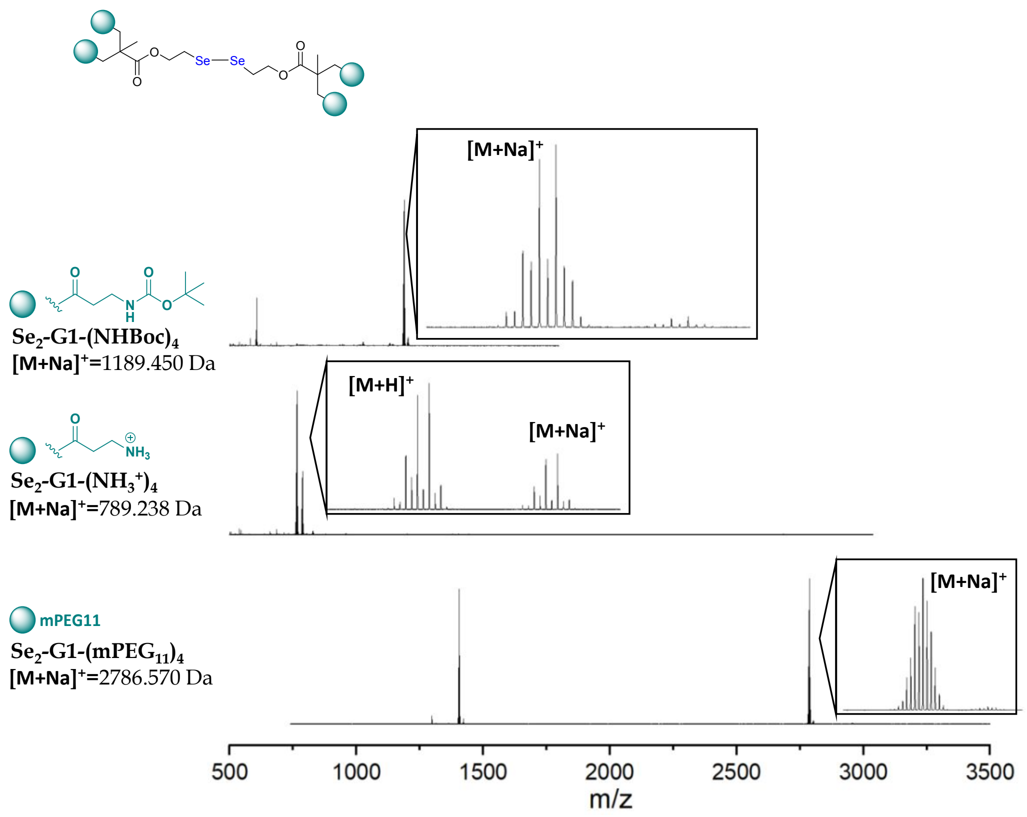

Figure S36. MALDI-ToF of the post functionalized dendrimers: Se_2-G1-$(NHBoc)_4$, Se_2-G1-$(NH_3^+)_4$ and Se_2-G1-$(mPEG_{11})_4$.

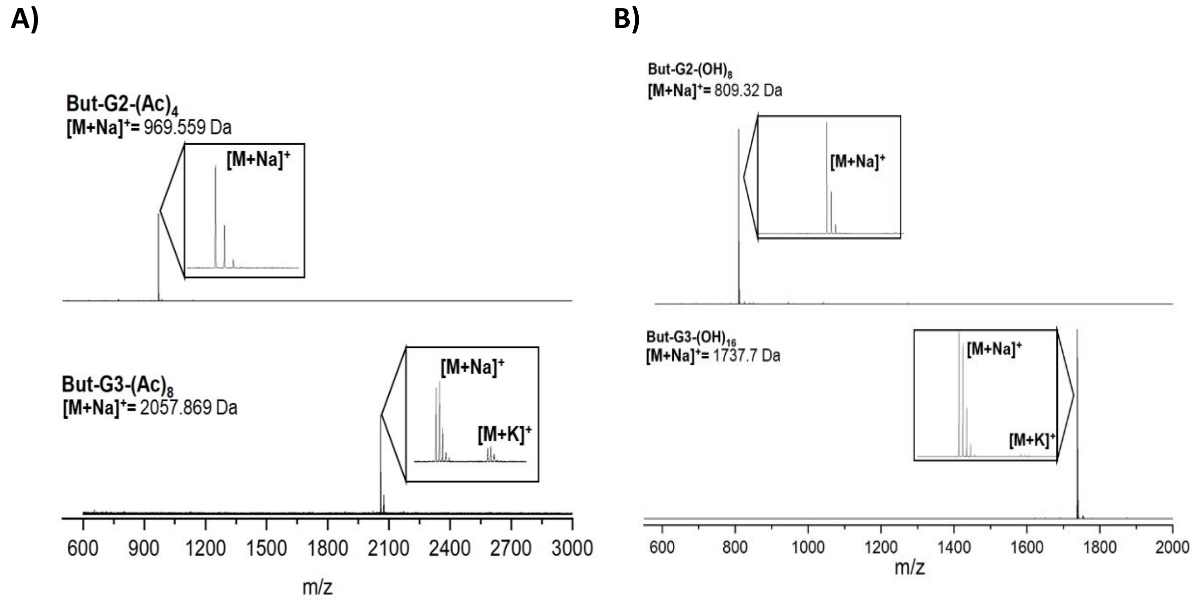

Figure S37. MALDI-ToF of the dendrimers A) But-Gn-$(Ac)_m$ and B) But-Gn-$(OH)_m$.

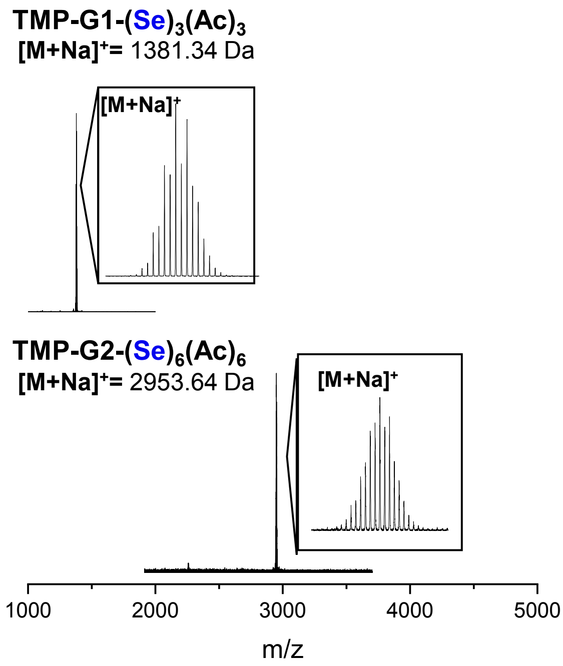

Figure S38. MALDI-ToF of the dendrimers TMP-Gn-(Se)$_m$(Ac)$_m$ where n=1-2 and m=3-6.

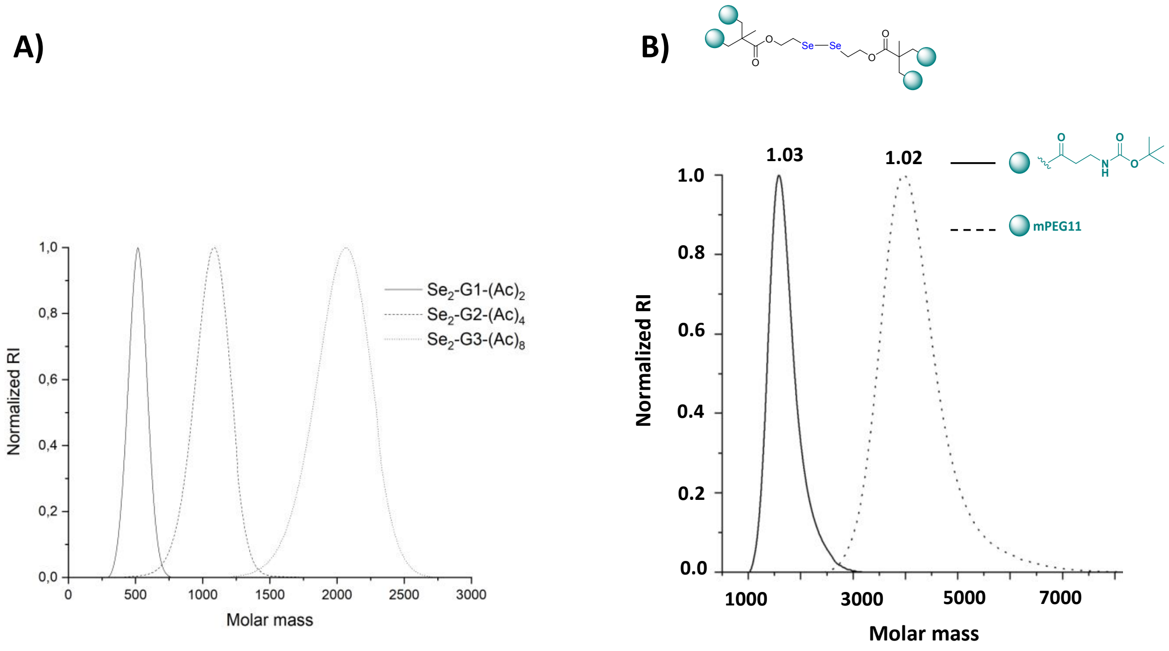

Figure S39. SEC analysis of A) Se$_2$-Gn-(Ac)$_m$ where n=1-3 and m=2-8 and B) Post functionalized first generation diselenide dendrimers.

A) B)

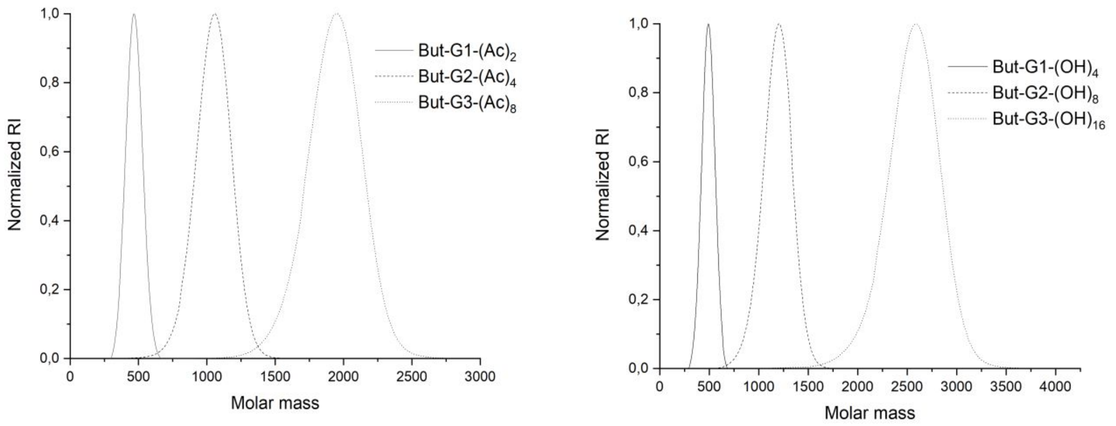

Figure S40. SEC analysis of A) But-Gn-$(Ac)_m$ where n=1-3 and m=2-8 and B) But-Gn-$(OH)_m$ where n=1-3 and m=4-16.

A) B)

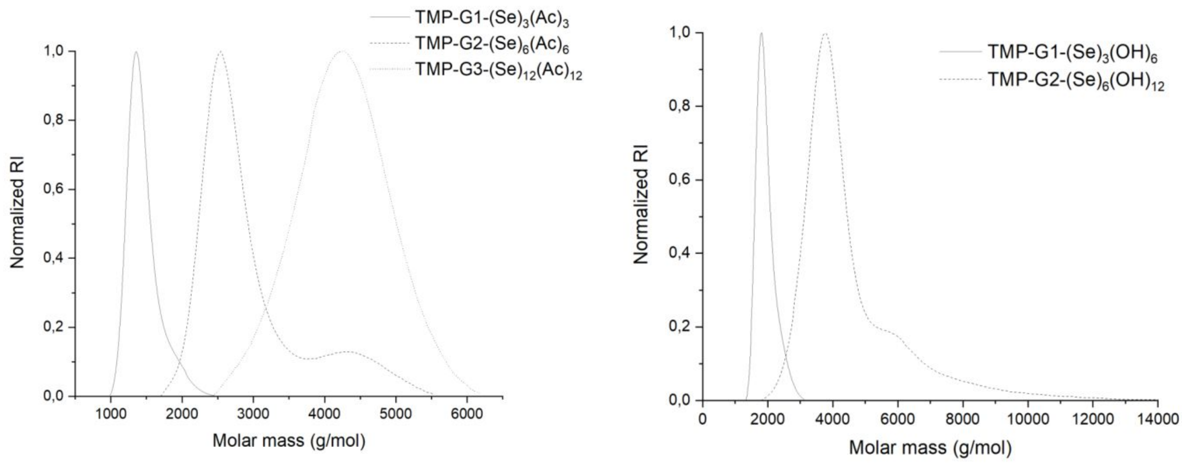

Figure S41. SEC analysis of A) TMP-Gn-$(Se)_m$ $(Ac)_m$ where n=1-3 ; m=3-12 and B) TMP-Gn-$(Se)_{m-3}(OH)_m$ where n=1-2 and m=6-12.

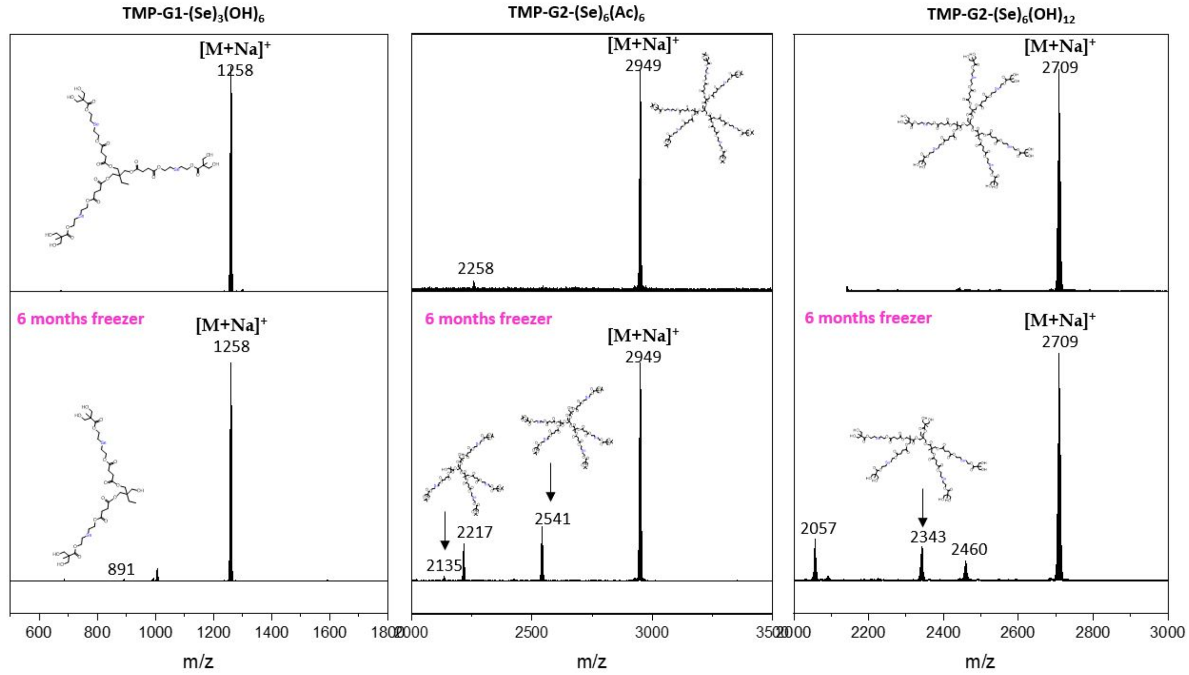

Figure S42. Stacked MALDI-ToF spectra of the first and second generation monoselenide dendrimers at time zero and after being stored in the freezer (-20 °C) for 6 months.

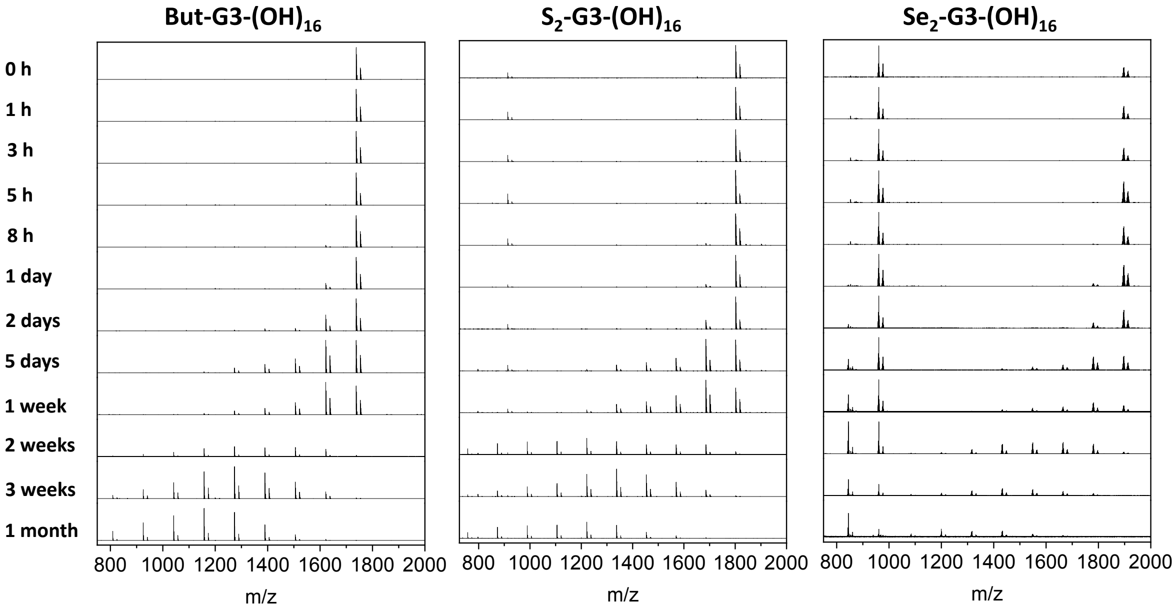

Figure S43. Degradation evaluation through MALDI-ToF of the dendrimers But-G3-(OH)$_{16}$, S$_2$-G3-(OH)$_{16}$ and Se$_2$-G3-(OH)$_{16}$ at pH 7.4 and different times.

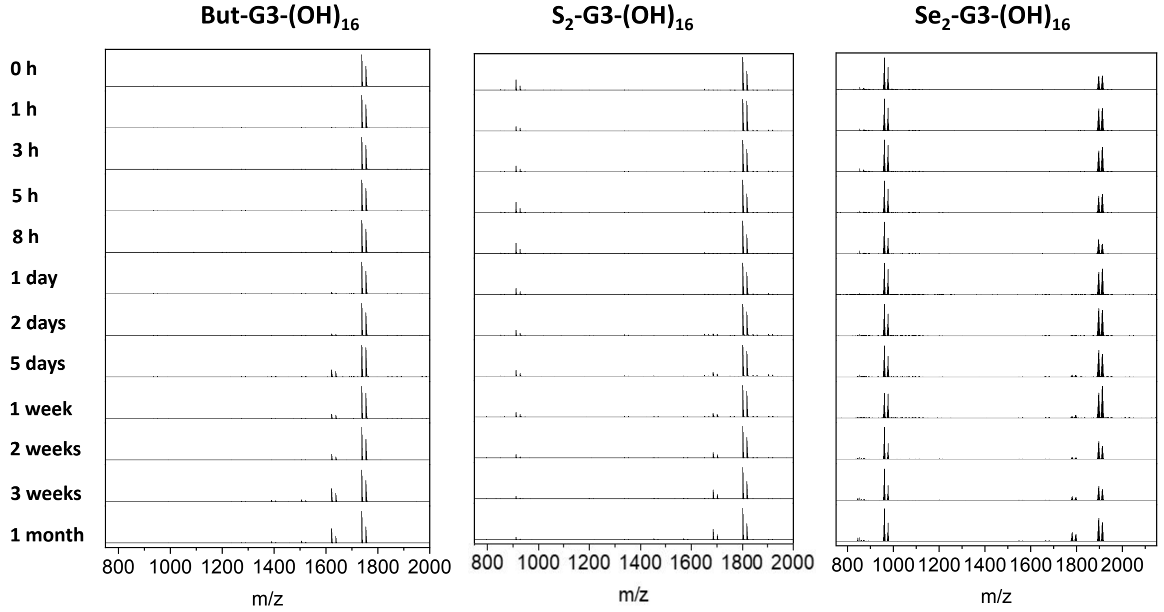

Figure S44. Degradation evaluation through MALDI-ToF of the dendrimers But-G3-$(OH)_{16}$, S_2-G3-$(OH)_{16}$ and Se_2-G3-$(OH)_{16}$ at pH 6.4 and different times.

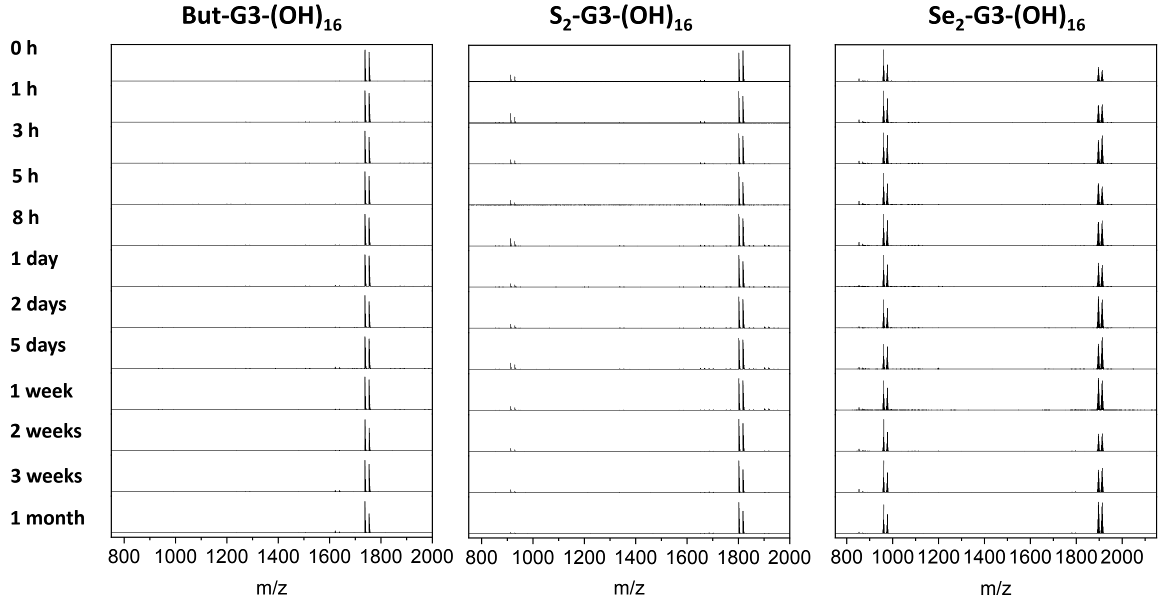

Figure S45. Degradation evaluation through MALDI-ToF of the dendrimers But-G3-$(OH)_{16}$, S_2-G3-$(OH)_{16}$ and Se_2-G3-$(OH)_{16}$ at pH 5.4 and different times.

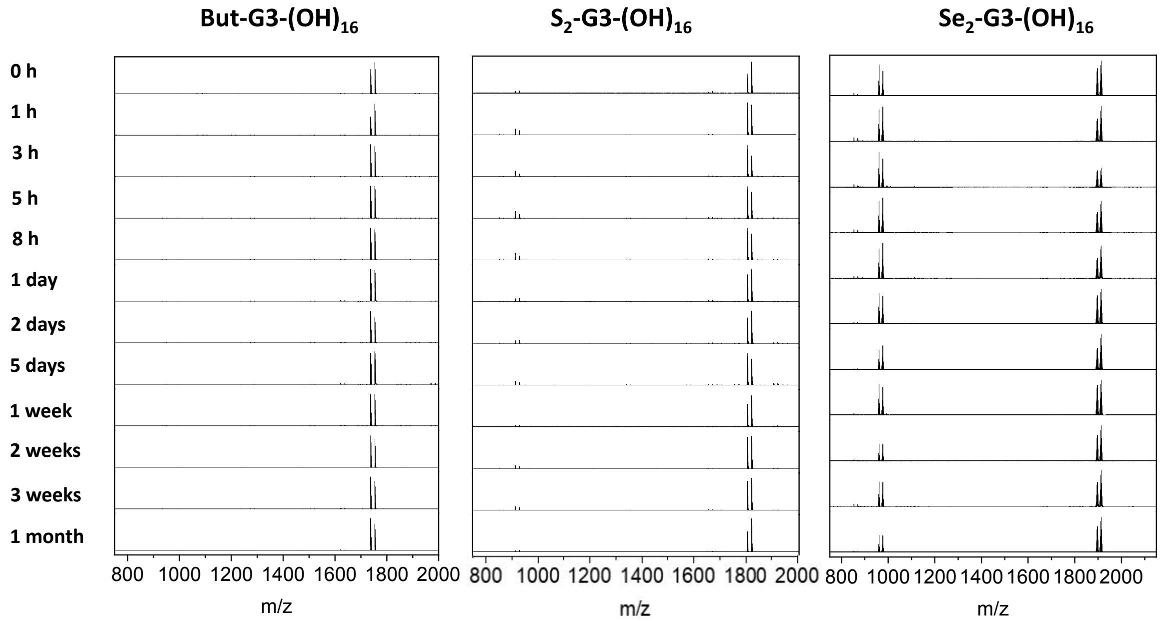

Figure S46. Degradation evaluation through MALDI-ToF of the dendrimers But-G3-$(OH)_{16}$, S_2-G3-$(OH)_{16}$ and Se_2-G3-$(OH)_{16}$ at pH 4.4 and different times.

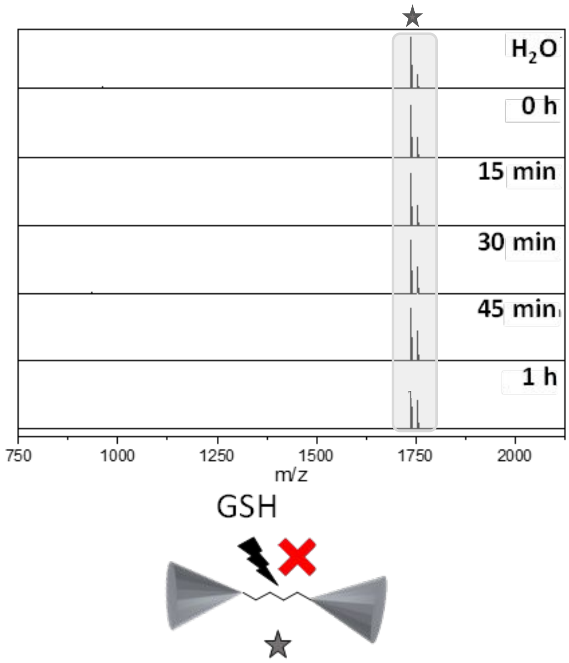

Figure S47. Degradation evaluation through MALDI-ToF of the dendrimers But-G3-$(OH)_{16}$, at pH 7.4 and 10 mM of Glutathione (GSH) at different times.

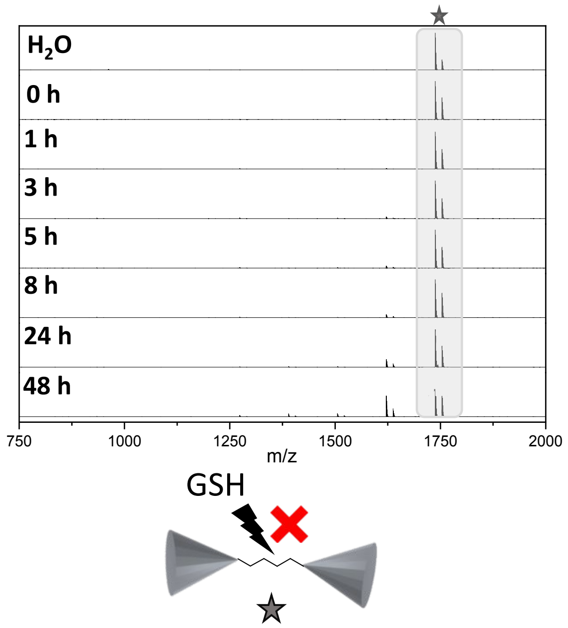

Figure S48. Degradation evaluation through MALDI-ToF of the dendrimers But-G3(OH)$_{16}$ at pH 7.4 and 10 µM of Glutathione (GSH) at different times.

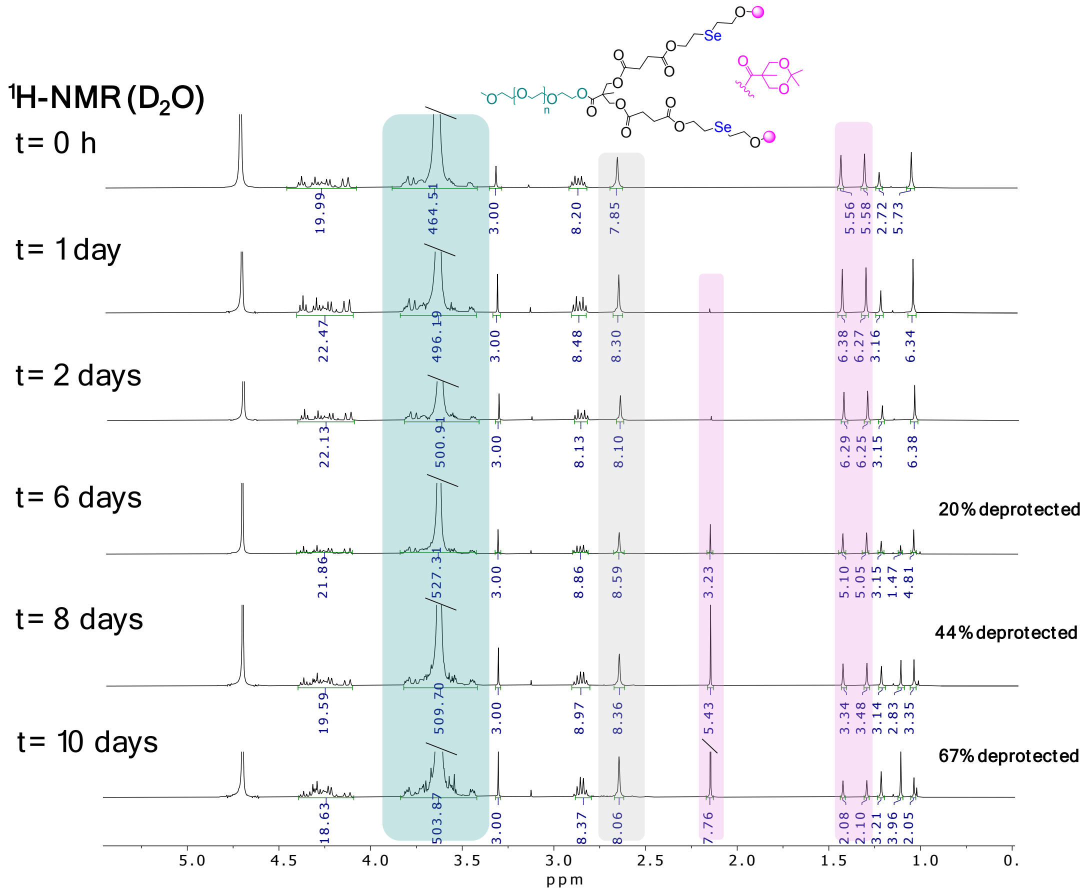

Figure S49. Stacked ^{1}H-NMR spectra of the second-generation LD acetonide protected (mPEG5k-G2-(Se)$_2$(Ac)$_2$) incubated in D_2O at 37°C over various time intervals.

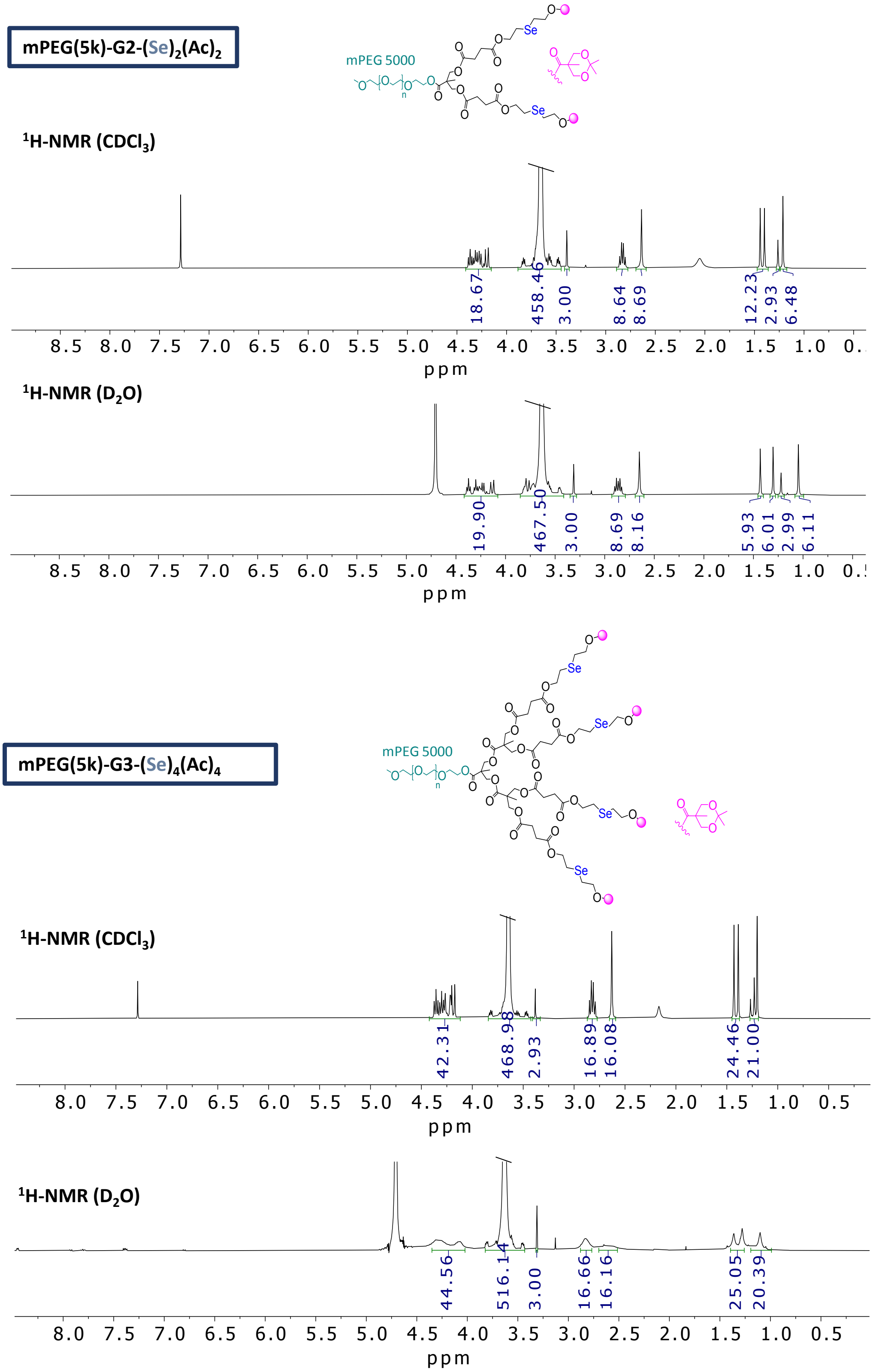

Figure S50. Stacked ^{1}H-NMR spectra of the second (mPEG5k-G2-(Se)$_2$(Ac)$_2$) and third generation (mPEG5k-G3-(Se)$_4$(Ac)$_4$) LDs in $CDCl_3$ and D_2O.

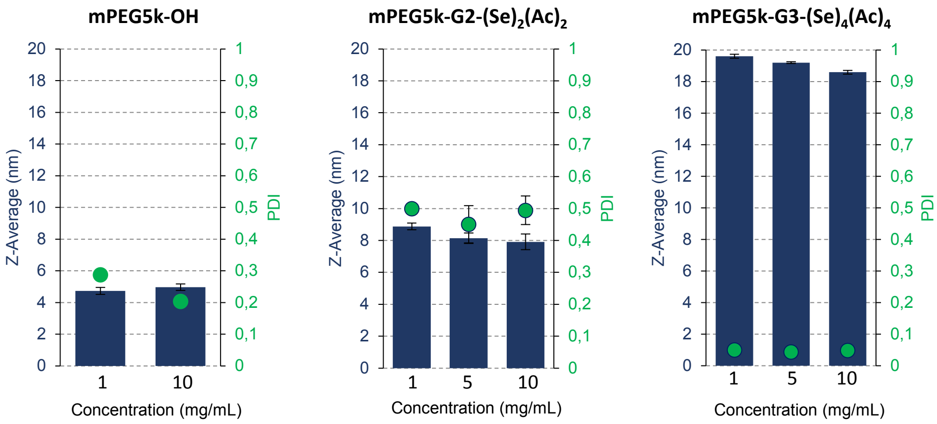

Compound (5 mg/mL)	D_i (nm)	D_v (nm)	D_n (nm)
mPEG5k-G2-$(Se)_2(Ac)_2$	5.5 ± 0.2*	4.2 ± 0.3	3.6 ± 0.3
mPEG5k-G3-$(Se)_4(Ac)_4$	20.4 ± 0.1	15.2 ± 0.0	13.9 ± 1.0

** Larger aggregates observed*

Figure S51. Hydrodynamic diameters and polydispersity index (PDI) obtained by DLS measurements of the commercially available mPEG5k-OH as well as LD polymers (mPEG5k-G2-$(Se)_2(Ac)_2$ and mPEG5k-G3-$(Se)_4(Ac)_4$) dissolved in PBS at 25 °C. D_i stands for Hydrodynamic diameter by intensity, D_v Hydrodynamic diameter by volume and D_n Hydrodynamic diameter by number.

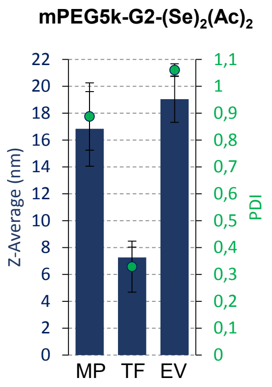

Compound	D_i (nm)	D_v (nm)	D_n (nm)
**mPEG5k-G2-$(Se)_2(Ac)_2$**			
Microprecipitation (MP)	4.8 ± 0.1*	4.4 ± 0.2	3.9 ± 0.0
Thin film method (TF)	5.3 ± 0.3*	3.8 ± 0.2	3.2 ± 0.4
Evaporation (EV)	5.1 ± 0.1*	4.2 ± 0.3	3.9 ± 0.0

** Larger aggregates observed*

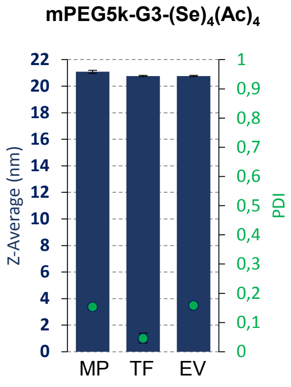

Compound	D_i (nm)	D_v (nm)	D_n (nm)
**mPEG5k-G3-$(Se)_4(Ac)_4$**			
Microprecipitation (MP)	22.9 ± 0.3	15.2 ± 0.0	13.5 ± 0.8
Thin film method (TF)	20.9 ± 0.2	15.2 ± 0.0	13.5 ± 0.8
Evaporation (EV)	22.5 ± 0.4	15.2 ± 0.0	12.3 ± 0.9

Figure S52. Hydrodynamic diameters and polydispersity index (PDI) obtained by DLS measurements of the LD polymers (mPEG5k-G2-$(Se)_2(Ac)_2$ and mPEG5k-G3-$(Se)_4(Ac)_4$)

formulated at 5 mg/mL under microprecipitation, thin-film and evaporation protocols in PBS at 25 °C. D_i stands for Hydrodynamic diameter by intensity, D_v Hydrodynamic diameter by volume and D_n Hydrodynamic diameter by number.

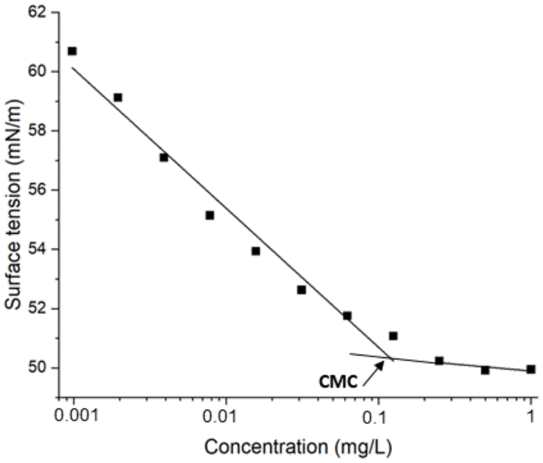

Figure S53. Surface tension measurements and linear approximation with 95 % confidence to attain the CMC of the third generation (mPEG5k-G3-$(Se)_4(Ac)_4$) at a range of concentrations between 0.001 and 1 mg/mL in PBS at 25 °C.

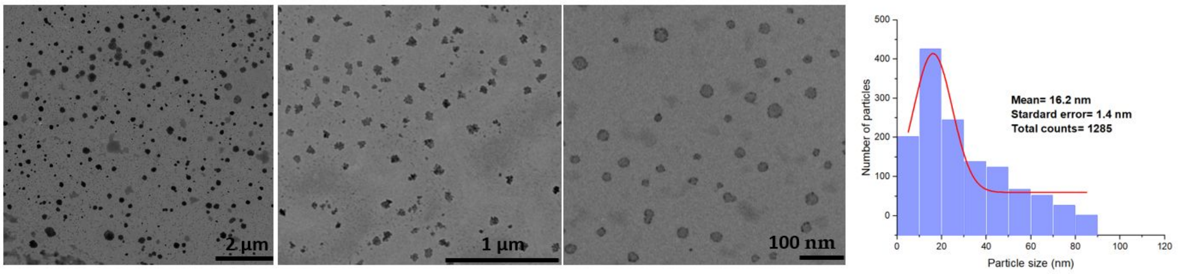

Figure S54. TEM pictures representative of micelles formed from mPEG5k-G3-$(Se)_4(Ac)_4$ at 0.5 mg/mL in MiliQ water as well as the histogram gathered from 1285 individual micelles from mPEG5k-G3-$(Se)_4(Ac)_4$ at 0.5 mg/mL with a distribution curve overlay and peak value.

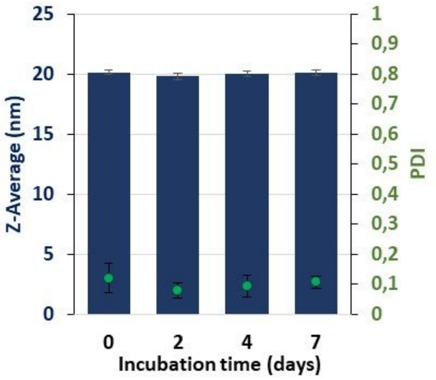

Figure S55. Stability study by DLS of the micelle formed from mPEG5k-G3-$(Se)_4(Ac)_4$ incubated at 37 °C in PBS (pH=7.4) at 1 mg/mL.

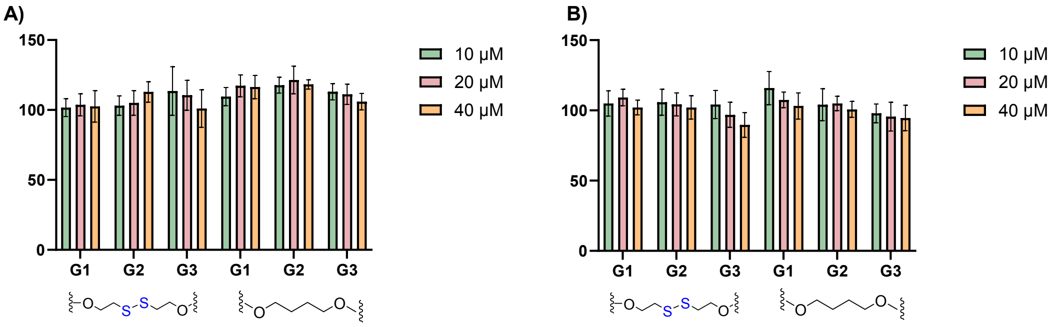

Figure S56. Cell viability percentages of 3T3 (a) and 4T1 (b) cells after 24h of treatment with a range of concentrations between 1 and 40 µM of CTRL dendrimers (S_2-Gn-$(OH)_m$ where n=1-3 and m=4-16 as well as But-Gn-$(OH)_m$ where n=1-3 and m=4-16). Mean values shown with error bars showing standard deviation, n = 2.

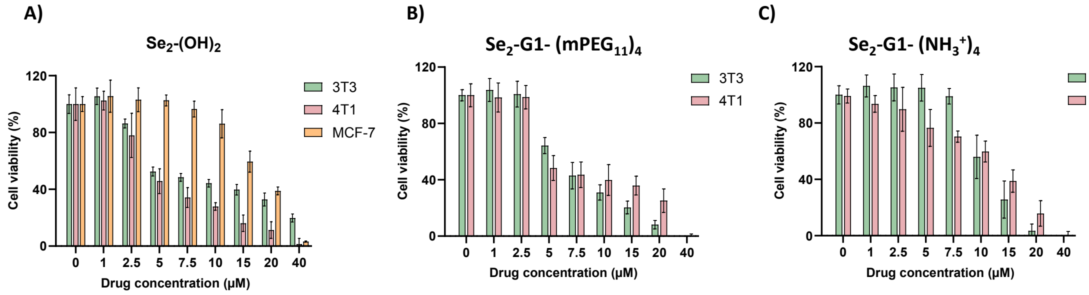

D)

	IC_{50} values in µM (mean ± SD)	
	**3T3**	**4T1**
Se_2-G1- $(mPEG_{11})_4$	7.07 ± 0.26	7.31 ± 1.22
Se_2-G1- $(NH_3^+)_4$	11.10 ± 1.20	10.77 ± 0.77

Figure S57. Cell viability percentages after 24h of treatment with a range of concentrations between 1 and 40 µM of a) the dendritic core (2-hydroxyethyl diselenide, Se_2-$(OH)_2$), b) first generation diselenide dendrimer post-functionalized with $mPEG_{11}$ (Se_2-G1-$(mPEG_{11})_4$) and c) first generation dendrimer functionalized with β-alanine (Se_2-G1-$(NH_3^+)_4$). d) IC_{50} values (in µM) for the post functionalized diselenide dendrimers. Mean values shown with error bars showing standard deviation, n ≥ 3.

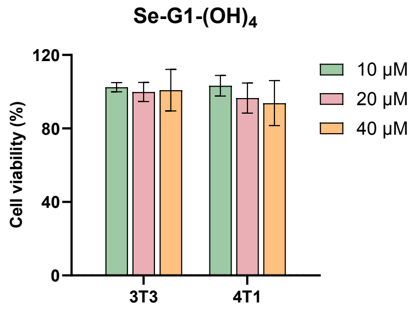

Figure S58. Cell viability percentages of 4T1 and 3T3 cells after 24h of treatment with 10, 20 and 40 µM of Se-G1-$(OH)_4$. Mean values shown with error bars showing standard deviation, n = 2.

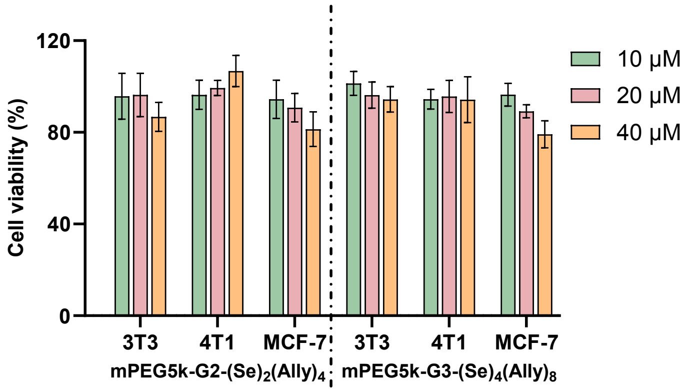

Figure S59. Cell viability percentages of 4T1 and 3T3 cells after 24h of treatment with 10, 20 and 40 µM of the allyl-functionalized linear dendritic (LD) polymers (mPEG5k-Gn-$(Se)_{m/2}(Allyl)_m$ where n=2 or 3 and m=2 or 4). Mean values shown with error bars showing standard deviation, n = 2.

References

1. Malkoch, M.; Malmström, E.; Hult, A., *Macromolecules* **2002,** *35* (22), 8307-8314.
2. Zhang, Y.; Mesa-Antunez, P.; Fortuin, L.; Andrén, O. C.; Malkoch, M., *Biomacromolecules* **2020,** *21* (10), 4294-4301.
3. Andrén, O. C.; Ingverud, T.; Hult, D.; Håkansson, J.; Bogestål, Y.; Caous, J. S.; Blom, K.; Zhang, Y.; Andersson, T.; Pedersen, E., *Advanced healthcare materials* **2019,** *8* (5), 1801619.
4. Walter, M. V.; Lundberg, P.; Hult, A.; Malkoch, M., *Journal of Polymer Science Part A: Polymer Chemistry* **2011,** *49* (13), 2990-2995.
5. García-Gallego, S.; Hult, D.; Olsson, J. V.; Malkoch, M., *Angewandte Chemie International Edition* **2015,** *54* (8), 2416-2419.
6. Andrén, O. C.; Zhang, Y.; Lundberg, P.; Hawker, C. J.; Nystrom, A. M.; Malkoch, M., *Chemistry of Materials* **2017,** *29* (9), 3891-3898.
